# Fan worms (Annelida: Sabellidae) from Indonesia collected by the Snellius II Expedition (1984) with descriptions of three new species and tube microstructure

**DOI:** 10.7717/peerj.9692

**Published:** 2020-08-18

**Authors:** María Ana Tovar-Hernández, Harry A. ten Hove, Olev Vinn, Michał Zatoń, Jesús Angel de León-González, María Elena García-Garza

**Affiliations:** 1 Universidad Autónoma de Nuevo León, Facultad de Ciencias Biológicas, Laboratorio de Biosistemática, San Nicolás de los Garza, Nuevo León, Mexico; 2 Naturalis Biodiversity Center, Leiden, The Netherlands; 3 Institute of Ecology and Earth Sciences, University of Tartu, Tartu, Estonia; 4 Institute of Earth Sciences, Faculty of Natural Sciences, University of Silesia in Katowice, Katowice, Poland

**Keywords:** Indonesian archipelago, Polychaeta, Feather duster worms, Tube microstructure

## Abstract

The Indonesian archipelago is one of the most diverse regions in the marine World. Many contributions on polychaete worms have been published since the Dutch Siboga Expedition to the Indonesian archipelago at the end of the 19th century. In this study, we examined specimens of Sabellidae *Latreille*, *1825* collected during the Snellius II Expedition (1984) to Indonesia, carried out by the Dutch Research Vessel (RV) “Tyro” and the Indonesian RV “Samudera”. The results include reports of *Acromegalomma acrophthalmos*, *A. interruptum*, *A*. sp., *Bispira manicata*, *B. porifera*, *B. secusoluta*, *Branchiomma boholense*, *Notaulax pyrrohogaster*, *N. tenuitorques*, *N*. sp. 3, *Parasabella crassichaetae*, *Perkinsiana anodina*, and *Sabellastarte spectabilis*. In addition, three new species are described: *Acromegalomma sumbense* sp. nov., *Claviramus olivager* sp. nov., and *Notaulax montiporicola* sp. nov., the latter in living coral (*Montipora nodosa*). Further, *Sabella* (*Potamilla*) *polyophthalmos* Grube is transferred to *Pseudopotamilla*. Additional histological accounts of *B. porifera* and tube microstructure of *A. acrophthalmos*, *B. porifera*, *P. anodina*, *Pseudopotamilla polyophthalmos* and *Sabellastarte spectabilis* are also included.

## Introduction

The Indonesian archipelago, the South China and the Philippine Seas are among the most diverse regions in the Western Pacific. Many contributions about polychaete worms have been published since the Dutch Siboga Expedition to the Indonesian archipelago at the end of the 19th century. A compilation of these Siboga reports can be found in Aguado, San Martín & ten Hove (2008) and Pamungkas & Glasby (2019).

Salazar-Vallejo et al. (2014) incorporated their findings in a checklist of the polychaete species originally described from China and Philippine Seas, including 26 species of sabellids described from the whole area. Ten of these from the Philippines where named by Adolph Eduard Grube, and three others from Singapore (Grube, 1878; Grube, 1881). Grube (1812–1880) was professor of Zoology in Breslau, nowadays Wrocław, and part of his collection still is present in the Museum of Natural History, Wrocław University (Wiktor, 1980). After his death, his private collection was bought by the Zoological Museum, Berlin (Hartwich, 1993). All sabellid species described by Grube are currently valid, except for *Sabella notata* Grube, 1878 (synonymized with *Sabellastarte spectabilis* Grube, 1878 by Knight-Jones & Mackie (2003)). Transfers of some species to other genera are proposed herein (Table 1).

**Table 1:** Sabellid species described by Grube (1878) and Grube (1881) from the Philippines and Singapore, including taxa not reported herein, with synonymies and current name.

Species name	Type locality	Synonymies and current name
*Myxicola ommatophora* Grube, 1878	Philippines	Original name currently valid
*Sabella acrophthalmos* Grube, 1878	Philippines	– *Megalomma acrophthalmos fide* Hartman, 1959; Knight-Jones, 1997; Tovar-Hernández & Carrera-Parra, 2011
		– *Acromegalomma acrophthalmos fide* Gil & Nishi, 2017
*Sabella* (*Dasychone*) *boholensis* Grube, 1878	Bohol, Philippines	– *Branchiomma boholense fide* Hartman, 1959; Knight-Jones, Knight-Jones & Ergen, 1991; del Pasqua et al., 2018
*Sabella* (*Dasychone*) *serratibranchis* Grube, 1878	Bohol, Philippines	– *Branchiomma serratibranchis fide* Hartman, 1959
		– *Pseudobranchiomma serratibranchis* fide Knight-Jones & Giangrande, 2003
*Sabella manicata* Grube, 1878	Bohol, Philippines	– *Bispira manicata fide* Knight-Jones & Perkins, 1998; Capa, 2008; Capa & Murray, 2015a
*Sabella notata* Grube, 1878	Bohol, Philippines	– *Sabellastarte indica fide* Fauvel, 1919
		– *Sabellastarte spectabilis fide* Knight-Jones & Mackie, 2003
*Sabella porifera* Grube, 1878	Bohol, Philippines	– *Bispira porifera fide* Knight-Jones & Perkins, 1998; Capa, 2008; Capa & Murray, 2015a
*Sabella* (*Potamilla*) *oligophthalmos* Grube, 1878	Singapore	– *Potamilla* (*Pseudopotamilla*) *oligophthalmos fide* Augener, 1914
		– *Potamilla oligophthalmos fide* Augener, 1926
		– *Pseudopotamilla oligophthalmos fide* Hartman, 1959
*Sabella* (*Potamilla*) *polyophthalmos* Grube, 1878	Philippines	– *Potamilla polyphthalmos fide* Hartman, 1959
		– *Pseudopotamilla polyphthalmos* (present study)
*Sabella* (*Potamilla*) *tenuitorques* Grube, 1878	Bohol, Philippines	– *Potamilla tenuitorques fide* Hartman, 1959
		– *Notaulax tenuitorques* (present study)
*Sabella pyrrhogaster* Grube, 1878	Bohol, Philippines	– *Notaulax pyrrhogaster fide* Perkins, 1984
*Sabella* (*Sabella*) *rufovittata* Grube, 1881	Singapore	– *Demonax rufovittata fide* Knight-Jones & Perkins, 1998
		– *Parasabella rufovittata fide* Tovar-Hernández & Harris, 2010
*Sabella spectabilis* Grube, 1878	Bohol, Masalac, Philippines and Singapore	– *Sabellastarte spectabilis fide* Knight-Jones & Mackie, 2003

An updated checklist of annelids from the South China Sea was provided by Glasby, Lee & Hsueh (2016). Their compilation includes both originally described species and also species occurring in the region but originally described from elsewhere: it contains 1257 species of Annelida, 37 of them corresponding to Sabellidae.

To Indonesia, Pamungkas & Glasby (2019) provided a detailed synthesis about the status of polychaete taxonomy and a checklist of 713 species, 23 of them belonging to Sabellidae. In addition, institutional repositories around the world housing Indonesian polychaete collections were indicated, and a list of authors who have formally described new Indonesian polychaete species.

During the 20th century, sabellid species from the Indonesian archipelago and the South China Sea have been described by Treadwell (1920), who reported *Sabellastarte spectabilis* (as *Sabella*) from Destacado Islands (Philippines). Augener (1933) reported four species of sabellids from Ambon, Banda and Biliton. Mesnil & Fauvel (1939) reported 11 species of sabellids collected by the Dutch Siboga Expedition, but they used some European, Mediterranean, Caribbean, Californian and South African species names, as usual at that time, when polychaetes were still assumed to have almost cosmopolitan distributions (but see Hutchings & Kupriyanova, 2018). Their material was catalogued for the Zoological Museum of Amsterdam (Bleeker & van der Spoel, 1992), presently in Naturalis Biodiversity Center (Leiden), but never has been restudied. Pillai (1965) reported three sabellid species from the Philippines; Gallardo (1968) included four species in his paper on polychaetes from Vietnam.

At the beginning of the 21st century, Fitzhugh (2002) reported 23 sabellin species from the West coast of Thailand in the Andaman Sea, including four new species: *Euchone cochranae* Fitzhugh, 2002, *Jasmineira labrofusca* Fitzhugh, 2002, *Laonome andamensis* Fitzhugh, 2002 and *Megalomma multioculatum* Fitzhugh, 2002 (the latter currently placed in *Acromegalomma*). Al-Hakim & Glasby (2004) reported seven species of sabellids off the Natuna Islands (South China Sea): *Bispira tricyclia* (Schmarda, 1861), *L. andamensis* and five undescribed species.

The series of papers on the Sabellidae from Australia by Capa (2007, 2008), Capa & Murray (2009), also includes some Indonesian material, either new species or new records such as *Acromegalomma interruptum* (Capa & Murray, 2009), *Bispira manicata* (Grube, 1878), *Pseudopotamilla monoculata* Capa, 2007, and *Stylomma palmatum* (de Quatrefages, 1866).

In a series of papers by Nishi and Nishi et al., four species were described from type localities within the South China Sea: *Acromegalomma miyukiae* (Nishi, 1998); *Jasmineira kikuchii* Nishi et al., 2009; *Notaulax yamasui* Nishi, Tanaka & Tovar-Hernández, 2019; and *Claviramus kyushuensis* Nishi, Tanaka & Tovar-Hernández, 2019; whereas *Paradialychone cincta* (Zachs, 1933) was reported from Kyushu (Nishi et al., 2009).

Hadiyanto (2018) reported fouling sabellids from Tanjung Priok, Port of Jakarta (Indonesia) as *Hypsicomus* sp. 1 and *Hypsicomus* sp. 2, which judging by his illustrations are species of the genera *Parasabella* Bush, 1905 for the former, and *Branchiomma* for the latter.

In this study, we examined specimens collected during the Snellius II Expedition to Indonesia (1984), carried out by the Dutch Research Vessel (RV) “Tyro” and the Indonesian RV “Samudera” (Van der Land & Sukarno, 1986).

## Materials and Methods

The material from the Snellius II Expedition is the main source of specimens in this study. Unless stated otherwise, it was collected by H.A. ten Hove, and is now deposited at the Naturalis Biodiversity Center. In addition, paratypes of *Claviramus kyushuensis* Nishi, Tanaka & Tovar-Hernández, 2019 from Japan, deposited at the Colección Poliquetológica from Universidad Autónoma de Nuevo León, México (UANL) were used for comparative purposes.

References for genera include only papers containing a generic diagnosis or relevant remarks for each genus. Identifications of specimens were based on original species descriptions and literature as referenced with each species. Information on localities of material examined is compiled from available labels, data bases and field notes.

Several measurements were taken: mid-thorax width, trunk length (from chaetiger 1 or collar to pygidium), radiolar crown length. Other features were counted such as numbers of radiolar pairs, number of thoracic and abdominal segments, and presence of gametes, or regenerations were noted. Descriptions contain standardized attributes for species according to a particular genus. However, as the distinctive features vary among genera, presentation and number of characters may not be the same along the manuscript. Full descriptions are provided only in the cases of new combinations and new species. Descriptions of new species were based on the holotypes; variation of paratype(s) as indicated between parentheses. Formulae describing frequency of unpaired compound eyes in different radioles on each side of the crown in *Pseudopotamilla* Bush, 1905 follow Knight-Jones et al. (2017): where R means right side of the crown in dorsal view, from dorsal-most radiole towards the ventral-most radiole and L refers to the left side of the crown in dorsal view, from dorsal-most radiole towards the ventral-most radiole. Numbers describe frequency of compound eyes and x = absent. For example: R x122211x; L xx111xx11x means that the right side of the crown presents 8 radioles: eyes absent in radiole 1 (dorsal-most radiole), one eye in second radiole, two eyes in radioles 3, 4 and 5, one eye in radioles 6 and 7, and radiole 8 without eyes. The left side of the crown contains ten radioles: eyes absent in radiole 1 (dorsal-most radiole) and radiole 2, one eye in radioles 3, 4 and 5, without eyes in radioles 6 and 7, one eye in radioles 8 and 9 and ventral-most radiole without eyes.

Diagnostic characters for some species or genera are tabulated; information is as complete as available in original descriptions and/or redescriptions as cited in Tables 2–7.

After embedding in paraplast, serial histological 7 μm sections were made from the thorax of *Bispira porifera* (Grube, 1878); sections were stained with haematoxylin-eosin and mounted permanently in synthetic resin (Sheehan & Hrapchak, 1980).

Observations were done with a Leica MZ75 stereomicroscope and an Olympus CH30 high power microscope. Photographs were taken with an attached Canon EOS Rebel T7i digital camera. Temporary Methyl green staining revealed thoracic glandular patterns in some species. Shirlastain A was helpful in analysis of the main morphological features in some species. The distribution map for *Acromegalomma* was produced using SimpleMappr (Shorthouse, 2010). Species properly illustrated, as indicated per species, were not figured again.

The tube microstructure of *Acromegalomma acrophthalmos* (Grube, 1878), *Bispira porifera* (Grube, 1878), *Perkinsiana anodina* Capa, 2007, *Pseudopotamilla polyophthalmos* (Grube, 1878) and *Sabellastarte spectabilis* (Grube, 1878) was studied using a scanning electron microscope. Ethanol preserved tubes were first air-dried and longitudinally as well as transversely cut with a razor blade. Samples were then coated with gold, and studied under high vacuum conditions with the environmental scanning electron microscope (ESEM) Philips XL30, at the Faculty of Earth Sciences, Sosnowiec, Poland. A single longitudinal and transverse section of each tube was studied. In addition the tube wall structure adjacent to the lumen of a single tube of each species was studied. The studied tubes were deposited at the Faculty of Earth Sciences in Sosnowiec, Poland.

### Nomenclatural acts

The electronic version of this article in Portable Document Format (PDF) will represent a published work according to the International Commission on Zoological Nomenclature (ICZN), and hence the new names contained in the electronic version are effectively published under that Code from the electronic edition alone. This published work and the nomenclatural acts it contains have been registered in ZooBank, the online registration system for the ICZN. The ZooBank LSIDs (Life Science Identifiers) can be resolved and the associated information viewed through any standard web browser by appending the LSID to the prefix http://zoobank.org/. The LSID for this publication is: [urn:lsid:zoobank.org:pub:382D313F-0138-4194-B3B2-5BA84817374A]. The online version of this work is archived and available from the following digital repositories: PeerJ, PubMed Central and CLOCKSS.

## Results

## Systematics

Order Sabellida Levinsen, 1883 (p. 180)
Family Sabellidae Latreille, 1825
Genus *Acromegalomma* Gil & Nishi, 2017 (pp 135–136; n.n. *pro Megalomma* Johansson, 1925).

*Megalomma* [junior homonym of the insect genus *Megalomma* Westwood, 1842].— Johansson, 1925: 9–10; Johansson, 1927: 130; Perkins, 1984: 351–352; Fitzhugh, 1989: 76; Knight-Jones, 1997: 314; Fitzhugh, 2003: 107; Tovar-Hernández & Salazar-Vallejo, 2008: 1953–1954; Giangrande & Licciano, 2008: 208; Capa & Murray, 2009: 204–205; Tovar-Hernández & Carrera-Parra, 2011: 14–15; Mikac, Giangrande & Licciano, 2013: 1514; Giangrande et al., 2015: 522–523; Giangrande et al., 2018: 57.

*Acromegalomma*.— Tovar-Hernández, de León-González & Bybee, 2017: 14.— Capa et al., 2019: 190–191.

**Type species**: *Branchiomma koellikeri* Claparède, 1869, a junior synonym of *Sabella lanigera* Grube, 1846, by monotypy of *Megalomma* Johansson (1925).

**Number of species**: 39, after Gil & Nishi (2017) and Tovar-Hernández, de León-González & Bybee (2017), including one new species described below.

**Remarks.** Diagnoses to genus level are available in Fitzhugh (1989), Tovar-Hernández & Salazar-Vallejo (2008), Capa & Murray (2009), Tovar-Hernández & Carrera-Parra (2011) and Capa et al. (2019). *Acromegalomma* was proposed by Gil & Nishi (2017) as a replacement name for *Megalomma* Johansson, 1925 (Annelida, Polychaeta, Sabellidae), preoccupied by *Megalomma* Westwood, 1842 (Insecta, Coleoptera, Carabidae). Gil & Nishi (2017: 135–136), gave 1926 as publication date of *Megalomma* Johansson, not 1925 or 1927 as used in some previous papers (e.g., Tovar-Hernández & Salazar-Vallejo, 2008). However, the correct date (see ICZN art. 21.8.1) is explicitly given at the end of his article (Johansson, 1925: 28 “tryckt den 5 November 1925”), the Arkiv för Zoologi printed every article separately. Nine species of *Acromegalomma* are distributed in the Indonesian archipelago, Australia and the South China and Philippine Seas, including a new species described below (Table 2).

**Table 2:** Species of *Acromegalomma* currently known from the Indo-Polynesian, Sino-Japanese, Southeastern Australian and Tasmanian provinces.

Species name	Occurrence of subterminal radiolar eyes	Dorsal collar margins	Caruncle	Keel	Dorsal lappets	Dorsal pockets	Anterior peristomial ring	Thoracic chaetae	Other relevant features	Type locality
*A. acrophthalmos* (Grube, 1878)	On most radioles	Fused to faecal groove	Present	Absent	Present	Present	Exposed dorsally between pockets	Type B	–	Singapore or Philippines
*A. cinctum* (Fitzhugh, 2003)	Dorsalmost, sometimes also in 2nd and 3rd pairs of radioles	Fused to faecal groove	Absent	Absent	Absent	Absent	Only partially exposed mid-dorsally	Type C	Glandular rings on chaetigers 2 and 3	Orchid Island, Taiwan
*A. inflatum* (Capa & Murray, 2009)	Dorsalmost, occasionally also in 2nd and 3rd following radioles	Fused to faecal groove	Absent	Present	Absent	Present	Well exposed	Type B	Inflated peristomium, protuding collar	NSW, Australia
*A. interruptum* (Capa & Murray, 2009)	Dorsalmost and lateral radioles	Not fused to faecal groove	Absent	Absent	Absent	Present	Well exposed	Type A	–	Queensland, Australia
*A. jubatum* (Capa & Murray, 2015a)	Dorsalmost and first 5 pairs of radioles	Fused to faecal groove	Present	Absent	Present	Present	Partially exposed	Type B	–	Lizard Island, Australia
*A. miyukiae* (Nishi, 1998)	First to 5th dorsalmost pairs of radioles	Not fused to faecal groove	Absent	Absent	Absent	Absent	Well exposed	Type A	–	Thailand, Andaman Sea
*A. multioculatum* (Fitzhugh, 2002)	On most radioles	Fused to faecal groove	Absent	?	Absent	Present	Well exposed	Type C	–	Thailand, Andaman Sea
*A. phyllisae* (Capa & Murray, 2009)	On most radioles, except ventralmost	Fused to faecal groove	Absent	Present	Present	Present	Partially exposed	Type B	–	Victoria, Australia
*A. sumbense* sp. nov.	Dorsalmost pair of radioles	Not fused to faecal groove	Present	Absent	Absent	Absent	Well exposed dorsally	Type B		Sumba, Indonesia

***Acromegalomma acrophthalmos* (Grube, 1878)**

(Figs. 1 and 2)

**Figure 1:**
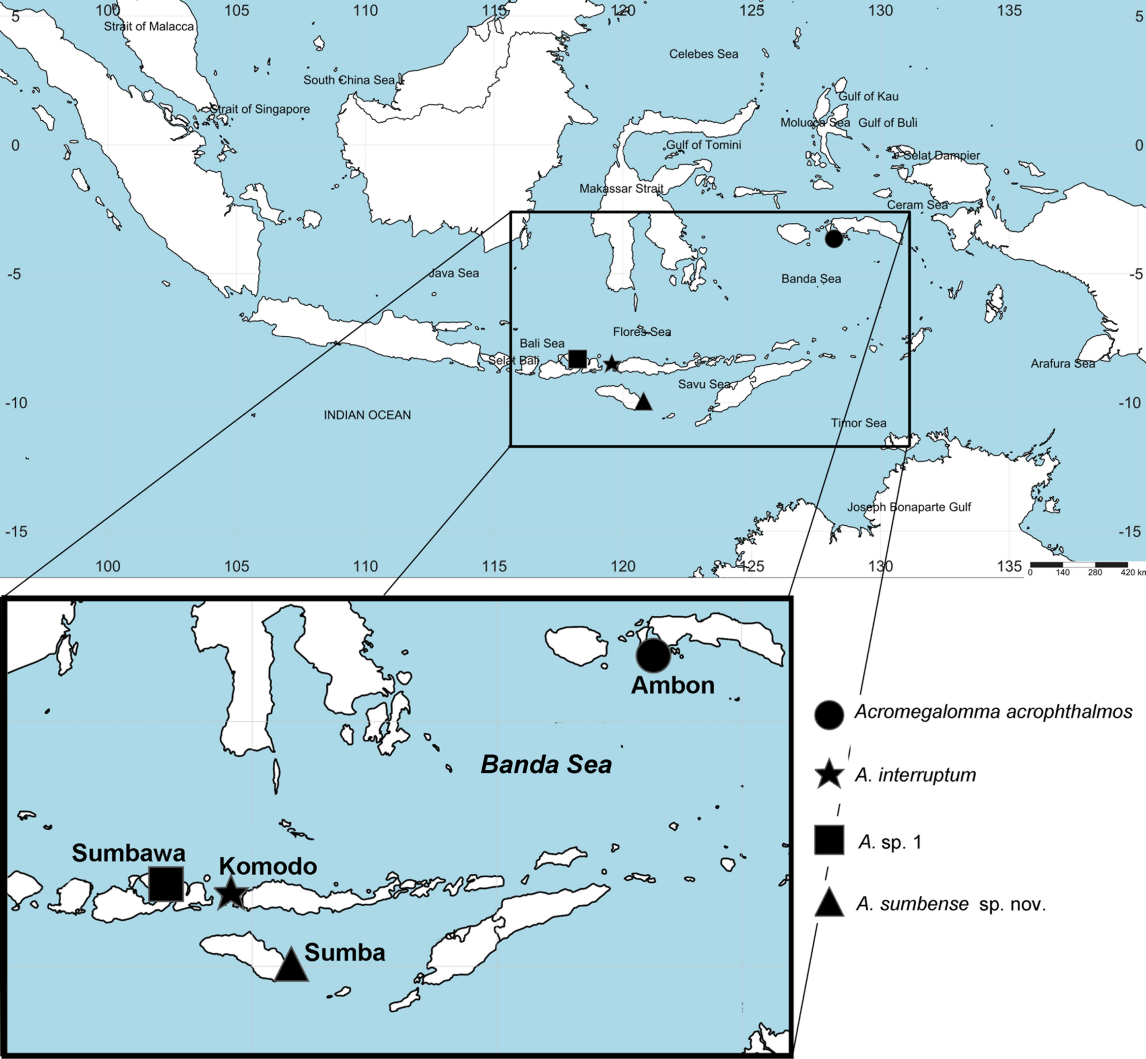
Distribution map of species of *Acromegalomma* from Indonesia.

**Figure 2:**
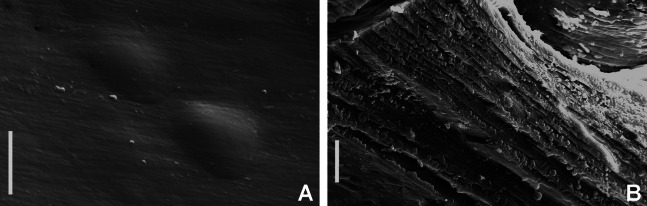
Tube microstructures of *Acromegalomma acrophthalmos*. (A) Surface of lumen showing fibers of single orientation, (B) transverse section showing laminar microstructure. Scale bars: 5 μm.

*Sabella acrophthalmos* Grube, 1878: 258–259; Wiktor, 1980: 280, holotype in Museum of Natural History, Wrocław University, MPW 364 (see remarks).

*Branchiomma acrophthalmum*.— Ehlers, 1920: 66.

*Megalomma acrophthalmos*.— Knight-Jones, 1997: 316, fig. 2A–L; Tovar-Hernández & Carrera-Parra, 2011: 15–17, fig. 2A–L.

*Acromegalomma acrophthalmos.—* Gil & Nishi, 2017: 136.

**Material examined.** Indonesian-Dutch Snellius II Expedition, Sta. 4.004B, Ambon Bay, inner bay near Poka, 03°39′S, 128°12′E (Fig. 1), mangroves and adjacent beach rock, scarce corals, coral rubble, seagrass, 2–3 m depth, September 4, 1984, 1 specimen [RMNH.VER. 19926].

**Description.** Large specimen, trunk ~60 mm long, 0.8 mm wide. Twenty two pairs of radioles. Subdistal eyes in most radioles (spherical and spiral). Anterior peristomial ring exposed between dorsal pockets. Posterior peristomial ring collar: dorsal margins fused to the faecal groove. Caruncle present. Dorsal lappets present. Inferior thoracic chaetae Type B (with progressively tapering distal tip). Interramal eyespots absent. Tube attached to large basal stone and composed of shell fragments, coralline sand and small stones. Maximum outer diameter of tube: 10 mm.

**Tube microstructure.** Tube’s lumen surface nearly smooth with few small bumps with sub-circular outline, without any regular pattern. Tube wall lamellar; lamellae thin, about 2–3 μm thick, straight in cross section. Lamellae composed of moderately developed, straight, long, thin and parallel fibers with relatively constant diameter about 0.12–0.20 μm. Fibers with interspaces as wide as fibers; interspaces filled with homogeneous smooth organics. The structure of lamellae is dense, solid, non-porous (Figs. 2A–2B).

**Remarks.** As usual in the 19th century, Grube did not specifically mark his specimens as types of any kind. On the basis of the fact that Grube (1878: 258) explicitly states that he had only one specimen, Wiktor (*loc. cit*.) identified it as the holotype.

Among the known species from the Indonesian archipelago, Australia and the South China and Philippine Seas (Table 2), only two have a caruncle: *A. jubatum* (Capa & Murray, 2015a) and *A*. *acrophthalmos*. The caruncle in some species of *Acromegalomma* was documented by Tovar-Hernández & Salazar-Vallejo (2008: figs 2A, E–F, 3–5). Externally, it resembles the caruncle in other polychaete families such as Amphinomidae and Spionidae. Internally, it is an organ innervated directly from the cerebral ganglion, supported with hyaline cartilage, homologous to the median organ of sabellariids (Tovar-Hernández & Salazar-Vallejo, 2008). Then, Capa & Murray (2009: fig. 10B) described a smooth structure –not homologous to the caruncle–, called keel. The keel is a smooth projection of the peristomium arising between the dorsal lips, forming a ventrally-directed ridge.

Major differences between *A*. *acrophthalmos* and *A. jubatum* are the following: eyes are present in most radioles in *A*. *acrophthalmos* (only in first six pairs of radioles in *A. jubatum*); long dorsal lappets in *A*. *acrophthalmos*, extending beyond collar margins (dorsal lappets shorter than collar margins in *A. jubatum*); and caruncle as long as collar length in *A*. *acrophthalmos* (only half as long as collar in *A. jubatum*).

Although *A. acrophthalmos* is one of the first sabellid species described from Indonesia, is about 50 mm long, and lives in the intertidal, there are scarce records of its presence in the region. *Acromegalomma acrophthalmos* is known from its type locality (Philippines); Ambon (Ehlers, 1920); Negros Island, Philippines (Tovar-Hernández & Carrera-Parra, 2011); and Ambon Bay, Maluku, Indonesia (present study).

Capa & Murray (2009) reported one specimen from Dampier Archipelago (Western Australia) as *Megalomma* cf. *acrophthalmos*, presenting a low, smooth keel. Later, Capa & Murray (2015a) reported another specimen from Lizard Island (Eastern Australia) (also as *Megalomma* cf. *acrophthalmos* but no specimen of *Megalomma cf. acrophthalmos* sensu Capa & Murray (2009)), having a caruncle, but the distal end of radioles were regenerating, eyes could thus not be studied. Type material of *Acromegalomma acrophthalmos* examined by Knight-Jones (1997: 316), and discussed in relation to their 20 specimens from the Philippines by Tovar-Hernández & Carrera-Parra (2011), as well as our specimen from Indonesia all have a caruncle. Detailed illustrations of morphological features of *A*. *acrophthalmos* can be found in Knight-Jones (1997) and Tovar-Hernández & Carrera-Parra (2011).

***Acromegalomma interruptum* (Capa & Murray, 2009)**

*Megalomma interrupta* Capa & Murray, 2009: 210–212, figs 2J–M, 4E–F, 5B, 7, 8; Capa & Murray, 2015a: 126–128, figs 11D–F.

*Acromegalomma interruptum*.— Gil & Nishi, 2017: 139.

**Material examined.** Indonesian-Dutch Snellius II Expedition, Sta. 4096A, Komodo, NE cape, 8°29′S, 119°34.1′E, reef patches in sand, 3 m depth, September 19–20, 1984, 1 specimen [RMNH.VER. 19927].

**Description.** Trunk 13 mm long, 2.2 mm wide. Sixteen pairs of radioles. Subdistal, spherical eyes in dorsalmost pair and lateral radioles. Anterior peristomial ring partially exposed dorsally. Posterior peristomial ring collar with dorsal margins not fused to faecal groove. Keel, caruncle, and dorsal lappets absent. Dorsal pockets shallow. Inferior thoracic chaetae Type A (distal end narrowing abruptly). Interramal eyespots absent. Tube not preserved.

**Remarks.** Among the species of *Acromegalomma* reported in the Indian Ocean, only two have dorsal collar margins not fused to faecal groove (Table 2): *A. interruptum* and *A. miyukiae*. Both species can be distinguished by the presence of shallow dorsal pockets in *A. interruptum* (absent in *A. miyukiae*) and eyes in dorsalmost and lateral radioles in *A. interruptum* (eyes in first dorsalmost pair of radioles in *A. miyukiae*).

*Acromegalomma interruptum* is known from One Tree Island (type locality) and Lizard Island, Australia (Capa & Murray, 2009; Capa & Murray, 2015a); Bay of Maumere, Pasir Sari, Indonesia (Capa & Murray, 2009) and Komodo, Indonesia (present study). Detailed illustrations of morphological features of *A*. *interruptum* can be found in Capa & Murray (2009) and Capa & Murray (2015a).

***Acromegalomma* sp. 1**

*Megalomma* sp. 1.— Capa & Murray, 2009: 218, 219, figs 2N–Q, 4I–J, 5E.

**Material examined.** Indonesian-Dutch Snellius II Expedition, Sta. 4.114, N of Sumbawa, Bay of Sanggar, 8°19.2′S, 118°14.4′E, lagoon side of reef barrier, September 21–22, 1984, 18–20 m, 1 specimen [RMNH.VER. 19928].

**Description.** Trunk 17.5 mm long, 3.5 mm wide. Radiolar crown 7.3 mm long. Fifteen pairs of radioles. Subdistal eyes present in dorsal and lateral radioles (large, spherical, surrounding the tip of dorsalmost pair of radioles; small, spherical, similar in size in lateral radioles). Anterior peristomial ring exposed partially on dorsal side. Posterior peristomial ring collar with dorsal margins fused to faecal groove; dorso-lateral margins with V-shaped notches. Dorsal pockets present, shallow. Keel present. Thoracic tori not contacting shields on anterior chaetigers. Ventral lappets rounded. Ventral sacs present. Inferior thoracic notochaetae Type B (with progressively tapering tips). Interramal eyespots absent. Tube not preserved. Body colour preserved only in dorsal thorax: brown coloured with residual dark spots located on the ventral margin of the thoracic tori.

**Remarks.** The presence of a keel has been reported in *A. inflatum*, *A. phyllisae* (Table 2) and *Acromegalomma* sp. 1 (as *Megalomma*) by Capa & Murray (2009). However, *A. inflatum* is easily discernible by the presence of a swollen peristomium, protruding from the collar. Eyes in *A. phyllisae* are present in all radioles, except in the ventralmost, whereas in *A*. sp. 1 from Queensland as well as from Indonesia, eyes are present in more than half of the radioles. Capa & Murray (2009) gave further differences of collar features between *A*. sp. 1 and *A. multioculatum* (Fitzhugh, 2002).

*Acromegalomma* sp. 1 was previously reported from Abbot Point, Queensland, Australia, at 7 m depth. Our specimen was collected in the Bay of Sanggar, Indonesia, at 18–20 m depth.

***Acromegalomma sumbense* sp. nov. Tovar-Hernández, ten Hove & de León-González**

(Figs. 3 and 4)

**Figure 3:**
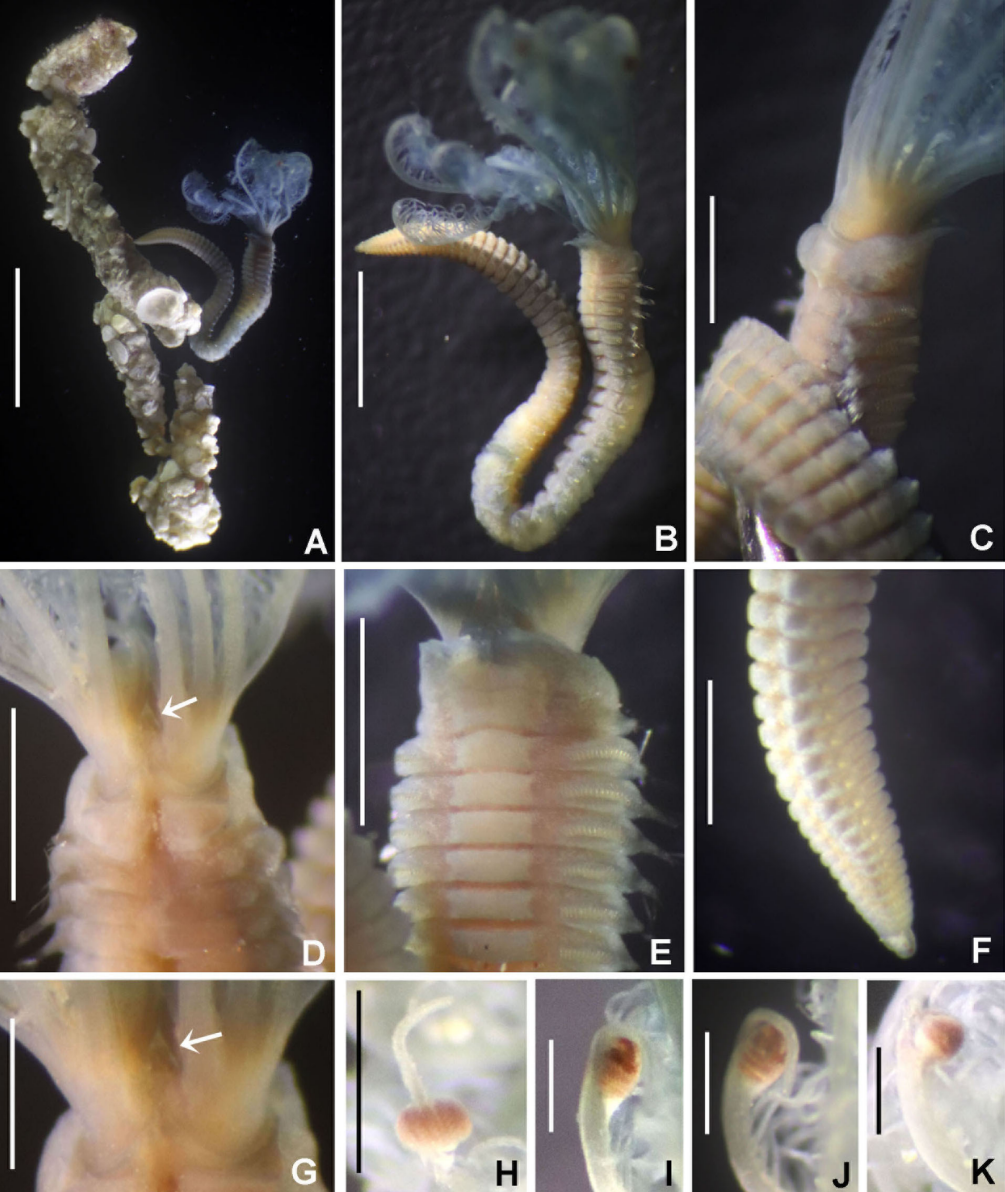
*Acromegalomma sumbense* sp. nov. (A) Worm and tube, (B) body, left lateral view, (C) thorax, right lateral view, (D) collar, dorsal view, caruncle indicated by arrow, (E) thorax, ventral view, (F) posterior abdomen, left lateral view, (G) detail of D, (H–K) radiolar eyes from dorsalmost pair, different views. Scale bars: (A) 3.5 mm, (B) 1.5 mm, (C, F–H) 0.6 mm, (D–E) 1 mm, (I–K) 0.2 mm. Holotype, RMNH.VER. 19929.

**Figure 4:**
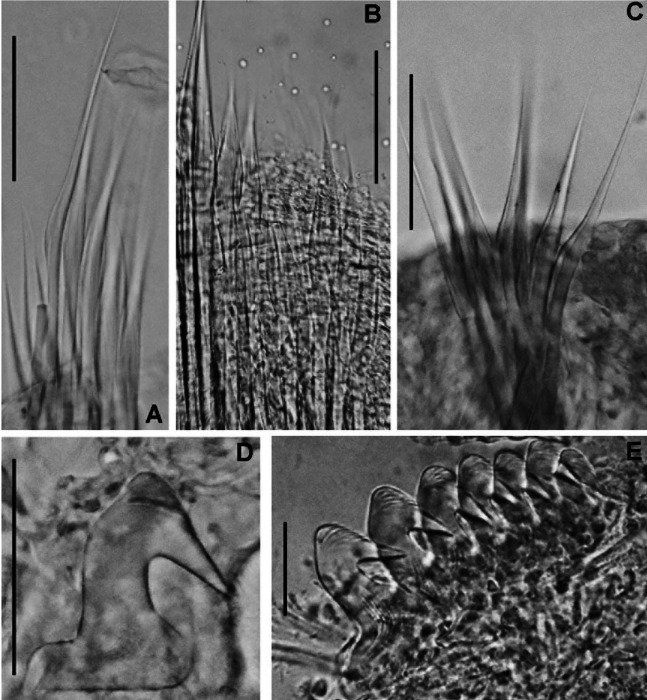
*Acromegalomma sumbense* sp. nov. (A) Elongate narrowly hooded notochaetae from collar, (B) thoracic notopodial fascicle with superior group of elongate, narrowly hooded chaetae, and inferior group of chaetae Type B (broadly hooded with progressively tapering distal tip), (C) abdominal fascicle with elongate narrowly hooded chaetae, (D) thoracic uncini, (E) abdominal uncini. Scale bars: (A–C) 60 μm, (D) 50 μm, (E) 30 μm. Holotype, RMNH.VER.19929.

LSID: urn:lsid:zoobank.org:act:A5F4957B-3DC0-49CA-B779-B10DCC80869B

**Material examined.** Holotype [RMNH.VER. 19929]: Indonesian-Dutch Snellius II Expedition, Sta. 4.068, NE coast of Sumba, 9°57′S, 120°48′E, 50 m, Agassiz trawl, sandy bottom with sponges and gorgonians, September 16, 1984. Paratype [RMNH.VER. 19930]: Sta. 4.051, NE coast of Sumba, E of Melolo, 9°53.5’S, 120°42.7′E, calcareous stones, rich epifauna dominated by soft corals, rectangular dredge, 75–90 m, September 13, 1984.

**Description.** *Colour, body shape, and size*. Body colour not preserved, except for yellow ventral shields. Holotype and paratype complete (Figs. 3A–3B), depressed. Trunk 8.4 mm long (12.5 mm), 1.1 mm wide (0.8 mm).

*Radiolar crown*. Length 3.2 mm (4.8 mm), twice longer than thorax. Radiolar lobes semi-circular. Eight pairs of radioles (10 pairs). Outer radiolar surface flattened. Subdistal compound eyes only in dorsalmost pair of radioles. Eyes large, oval in side view (Figs. 3I and 3J), rounded in frontal view (Figs. 3H and 3K). Radiolar tips as long as three times ocular diameter (Fig. 3H). Dorsal lips erect, triangular, about 1/4 as long as radiolar crown, with radiolar appendages (mid-rib). Two pinnular appendages. Ventral lips about 1/4 as long as dorsal lips, broadly rounded.

*Peristomium*. Anterior peristomial ring fully exposed dorsally, protruding, swollen (Fig. 3D). Caruncle present, short, triangular (Figs. 3D and 3G), 1/2 as long as second thoracic segment, rough surface formed by irregularly sinuous crests. Posterior peristomial ring collar with dorsal collar margins not fused to faecal groove (Fig. 3D). Dorsal lappets and dorsal pockets absent. Ventral lappets short, triangular, with a mid-ventral incision reaching anterior margin of ventral shield of collar (Fig. 3E). Lateral collar margin as oblique, not covering bases of radioles (Figs. 3C and 3D). Ventral sacs and ventral lateral lamellae present.

*Thorax*. Chaetiger 1: notochaetae only elongate narrowly hooded; superior row longer than inferior (Fig. 4A). Ventral shield of chaetiger 1 with rounded anterior margin and a short, anterior medial incision. Chaetigers 2–8: tori not contacting shields. Notopodial fascicles with superior group of elongate, narrowly hooded chaetae; inferior groups of chaetae Type B (with progressively tapering tips) (Fig. 4B). Uncini with main fang surmounted by several rows of numerous minute teeth; dentition covering half of main fang length (Fig. 4D), handles 1.5–2 times longer than main fang. Companion chaetae with teardrop-shaped membranes. Interramal eyespots absent.

*Abdomen*. Segments: 52 (52). Neurochaetae narrowly hooded (Fig. 4C); chaetae in posterior rows longer than in anterior rows. Uncini with main fang surmounted by several rows of teeth, dentition covering a half of main fang length, handles 1.5 times longer than main fang (Fig. 4E). Interramal eyespots absent. Pygidium broadly rounded (Fig. 3F) with two groups of red eyespots.

*Tubes*: composed of shell fragments and coralline sand (Fig. 3A).

*Sex and gametes*: Unknown.

**Remarks.** *Acromegalomma sumbense* sp. nov., resembles the specimens reported as *Megalomma* sp. 2 by Capa & Murray (2009) from Victoria (Australia) and referred herein to *Acromegalomma* sp. 2, *A. kaikourense* (Knight-Jones, 1997), described from New Zealand, and *A*. sp. cf. *kaikourense* (Capa & Murray, 2015a), from Lizard Island (Australia). All three taxa have dorsal collar margins not fused to the faecal groove, and eyes only in the dorsalmost radiolar pair. However, *A. sumbense* sp. nov., specimens differ from those taxa by having a caruncle (absent in the others) and radiolar tips as long as three times the diameter (radiolar tips not extending beyond distal margins of eyes in *Acromegalomma* sp. 2, and tip about as long as ocular diameter in *A. kaikourense* and *A*. sp. cf. *kaikourense*). In addition, *A. kaikourense* and *A*. sp. cf. *kaikourense* have vestigial dorsal pockets and dorsal lappets (both absent in *A. sumbense* sp. nov.).

Among the *Acromegalomma* species distributed in Indonesia, Australia, South of China and Philippines Sea, only three have caruncles: *A. acrophthalmos*, *A. jubatum* and *A. sumbense* sp. nov. *Acromegalomma acrophthalmos* has eyes on most radioles; eyes in *A. jubatum* are located in the dorsalmost and first five pairs of radioles; and *A. sumbense* sp. nov., has eyes only in dorsalmost pair (Table 2).

**Etymology.** The specific name is an adjective derived from Sumba, the type locality.

**Genus *Bispira*Krøyer, 1856** (p. 13)

*Bispira*.— Fitzhugh, 1989: 72; Knight-Jones & Perkins, 1998: 405–405; Capa, 2008: 306–307; Faasse & Giangrande, 2012: 592–593; Cepeda & Lattig, 2017: 5–6; Tovar-Hernández, de León-González & Bybee, 2017: 6; Capa et al., 2019: 192.

**Type species**: *Amphitrite volutacornis* Montagu, 1804, subsequently designated by Bush (1905).

**Number of species**: 23, after Cepeda & Lattig (2017) and Capa et al. (2019).

**Remarks.** Diagnoses to genus level are available in Fitzhugh (1989), Knight-Jones & Perkins (1998), Capa (2008), Cepeda & Lattig (2017) and Capa et al. (2019). Five species of *Bispira* have been recorded from the Indian Ocean. In Table 3 a comparison of these species is provided.

**Table 3:** Species of *Bispira* currently known from the Indo-Polynesian province.

Species name	Radiolar eyes	Radiolar flanges	Dorsal basal flanges	Ventral collar margins	Dorsal spongy cushions	Other relevant features	Type locality
*B. manicata* (Grube, 1878)	1–3 pairs	Absent, narrow or discontinuos	Absent	Inrolled	Absent	–	Bohol, Philippines
*B. porifera* (Grube, 1878)	Absent	Narrow	Absent	Smooth	Present	–	Bohol, Philippines
*B. secusoluta* (Hoagland, 1920)	Absent	Narrow, wider distally	Absent	Smooth	Absent	Paired patches of cilia in ventral shields	Sombrero Islands, Philippines
*B. serrata* Capa, 2008	Paired along radiolar length	Broad	Present	Smooth	Absent	Serrated radiolar flanges	Queensland, Australia
*B. tricyclia* (Schmarda, 1861)	1–2 pairs	Absent basally, vestigial distally	Absent	?	Absent	Unispiral crown	Sri Lanka
*B*. sp. A (as in Capa, 2008)	1–2 pairs	Increasing in length distally	Absent	Smooth	Absent	–	Victoria, Australia

***Bispira*** ***manicata* (Grube, 1878)**

(Figs. 5A–5C)

**Figure 5:**
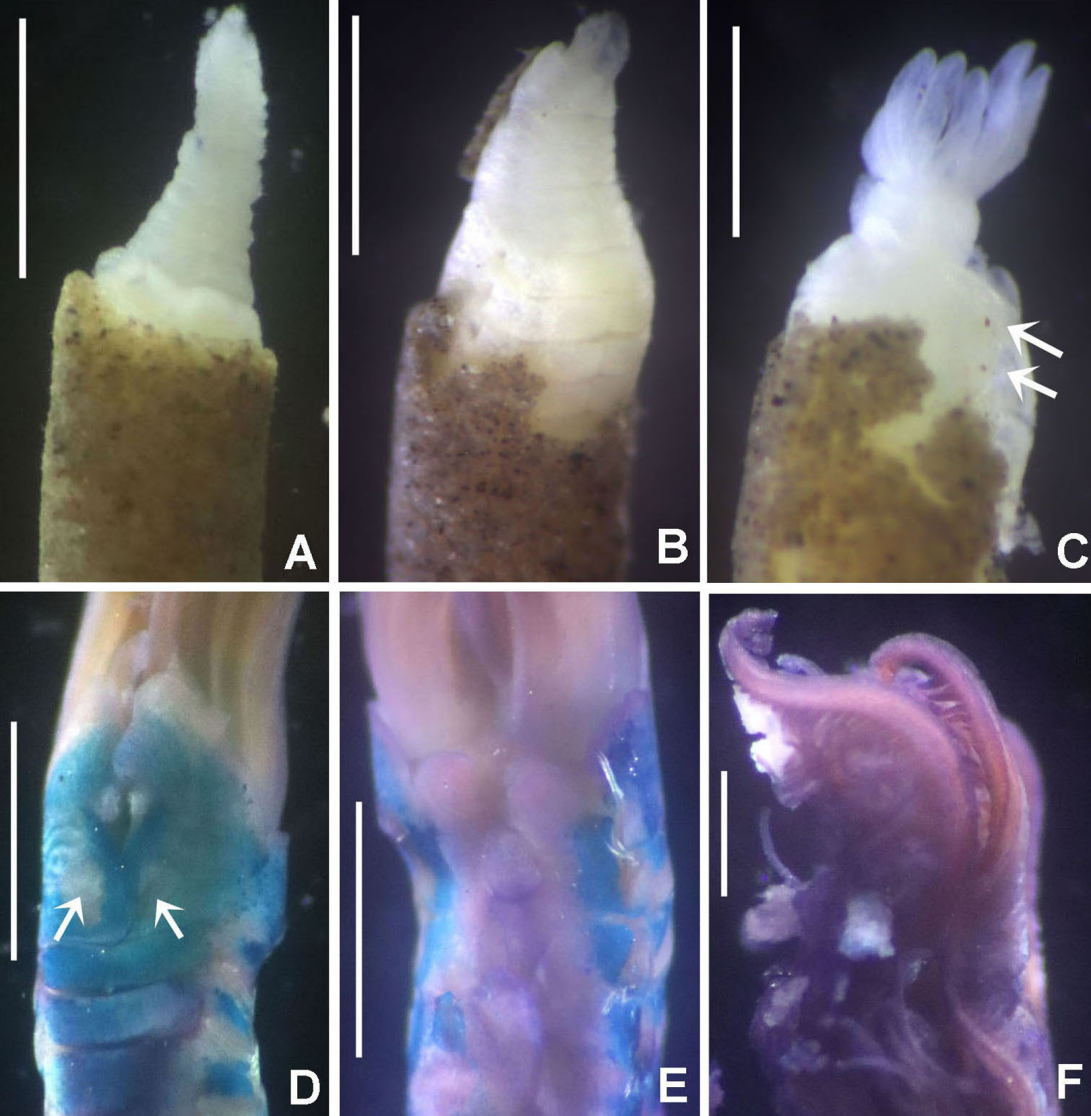
*Bispira manicata* and *Bispira secusoluta*. (A–C) Regenerating anterior buds of *B. manicata* from the least developed stage (A) to the most developed stage (C), arrows in C indicate interramal eyespots. (D–F) *Bispira secusoluta*. (D) collar, ventral view, (E) collar, dorsal view, (F) radioles. Arrows in D, indicate ventral, ciliary pads. Scale bars: (A–C, F) 0.5 mm, (D–E) 1 mm. Stain: (D–E) methyl green. Specimens: (A–C) RMNH.VER.19931, (D–F) RMNH.VER. 19933.

*Sabella manicata* Grube, 1878: 255–266, pl. 14, fig. 3; Wiktor, 1980: 280, holotype in Museum of Natural History, Wrocław University, MPW 366 (see Remarks under *Acromegalomma acrophthalmos*).

**Figure 6:**
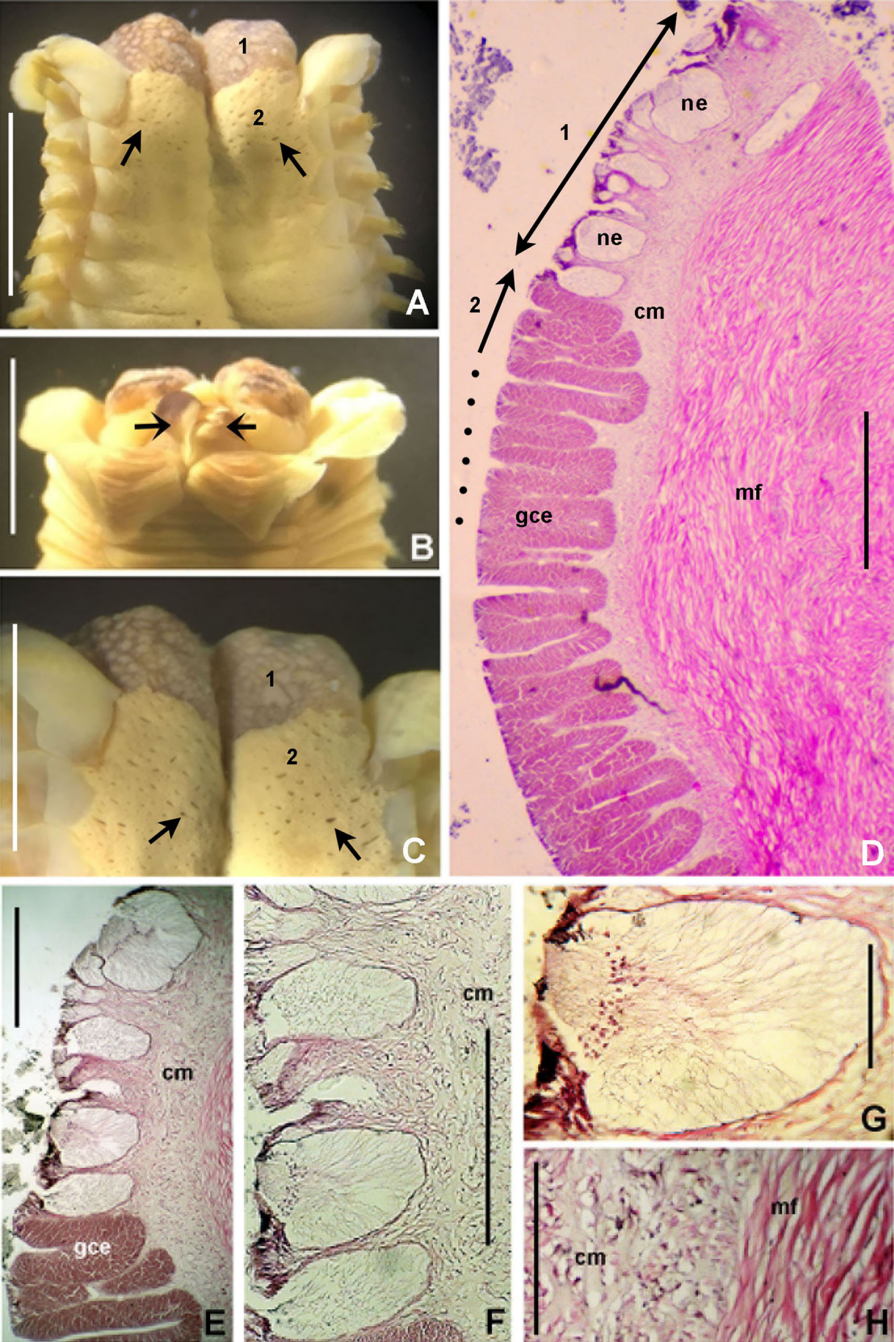
*Bispira porifera*. (A) Thorax, dorsal view, (B) collar, ventral view, (C) detail of A, peristomial (1) and thoracic (2) spongy cushions, dorsal view, (D) histological, longitudinal section of peristomial and thoracic cushions, (E–G) detail of nerves in peristomial spongy cushion, (H) detail of cartilaginous matrix and muscular fibers. Arrows in (A) and (C) indicate tissue openings, in (B) arrows indicate ventral sacs. Scale bars: (A–C) 3 mm, (D, F) 500 μm, (E) 250 μm, (G) 125 μm, (H) 50 μm. Numbers and letters stand for: (1) peristomial cushion, (2) thoracic cushion, cm, cartilage matrix; gce, glandular columnar epithelium; mf, muscular fibers; ne, nerves. Stain: (D–H) haematoxilin-eosin. Single specimen, RMNH.VER. 19932.

*Bispira manicata*.— Knight-Jones & Perkins, 1998: 424–426, fig, 15; Capa, 2008: 309, 311, 313–314, figs 4G–N, 5A–G, 6; Capa & Murray, 2015a: 104, fig. 2A–F.

**Material examined.** NNM Derawan Islands, Indonesia, legit Lisa Becking, September 20, 2008, BER, LE 341, 19 specimens [RMNH.VER. 19931].

**Description.** Trunk 6–12 mm long, 0.4–1 mm wide. Radiolar crown 3–5 mm long with 6–8 pairs of radioles. Two or three pairs of compound radiolar eyes, semi-spherical in shape, arranged on proximal half of radioles. Radiolar flanges narrow, continuous along radiolar length. Dorsal basal flanges absent. Dorsal lips deep purple, with radiolar appendages. Posterior peristomial ring collar with ventral margins forming rounded lappets. Thorax with 8–16, abdomen with 48–64 chaetigers.

**Remarks.** The material fits descriptions by Knight-Jones & Perkins (1998), Capa (2008) and Capa & Murray (2015a), from Bohol Island, Philippines (type locality) and Australia (Capa, 2008; Capa & Murray, 2015a). *Bispira manicata*, *B. porifera* (Grube, 1878) and *B. secusoluta* (Hoagland, 1920) were originally described from the Philippines (Table 3). *Bispira porifera* is remarkable in having notorious dorsal spongy cushions, whereas *B. manicata* has one to three pairs of radiolar eyes as opposed to *B. secusoluta* with none (Table 3).

Tubes of Indonesian specimens are composed from brown dark sand and architomy is present in some specimens. It includes worms undergoing pre-fission or post-fission. In reproducing worms prior to fission, the posterior abdomen was cream coloured, tapering abruptly towards the posterior end (Fig. 5A). Buds or fragments separated from a parental worm (post-fission) showed incomplete regeneration: rudimentary crowns and –as yet– only abdominal segments (Figs. 5A–5C). The architomy in *B. manicata* is similar to that described for the Caribbean *B. brunnea* (Dávila-Jiménez, Tovar-Hernández & Simões, 2017).

***Bispira secusoluta* (Hoagland, 1920)** (species name corrected for gender agreement)

(Figs. 5D–5F)

*Sabella secusolutus* Hoagland, 1920: 627, pl. 52, figs 7–13.

*Bispira secusolutus*.— Knight-Jones & Perkins, 1998: 437–439, fig. 22.

**Material examined.** Indonesian-Dutch Snellius II Expedition, Sta. 4.114, N of Sumbawa, Bay of Sanggar, 8°19.2′S, 118°14.4′E, lagoon side of reef barrier, September 21–22, 1984, 18–20 m depth, 1 specimen [RMNH.VER. 19933].

**Description.** Trunk 7 mm long (incomplete specimen), 0.8 mm wide. Radiolar crown 6 mm long (twice longer than thorax) with six pairs of radioles. Radiolar eyes absent. Narrow flanges along radiolar length (Fig. 5F). Palmate membrane 1/4 as long as radiolar crown, or as three thoracic segments. Dorsal lips as long as palmate membrane. Rounded lobes medial to dorsal lips. Posterior peristomial ring collar with dorsal margins not fused to faecal groove (Fig. 5E), lateral margins notched. Ventral lappets long, triangular, with small, triangular-like extensions overlapping at midline (Fig. 5D). Ventral shield of collar with two large, lung-shaped ciliated areas (Fig. 5D). Thorax with eight and abdomen with 21 chaetigers. Interramal eyespots present along thorax and abdomen. Thoracic and abdominal shields with a pair of oval to rounded patches of cilia per shield. Tube not preserved.

**Remarks.** This species was described from Sombrero Islands (Philippines) by Hoagland (1920) as *Sabella secusolutus*, then transferred to *Bispira* by Knight-Jones & Perkins (1998). The word *solutus* is a Latin adjective, *Sabella* is feminine (as is *Bispira*) and the species name should have been *secusoluta* –with feminine ending, ICZN, 1999, Art.34– and is corrected here. The record of *Bispira secusoluta* in Sanggar Bay is the first record of the species since its description in 1920.

*Bispira secusoluta* and four of its congeners lack radiolar eyes: *B. brunnea*, *B. porifera*, *B. wireni* (Johansson, 1922) and *B. oatesiana* (Benham, 1927). *Bispira brunnea* and *B. secusoluta* have ventral lappets with triangular extensions overlapping at midline (without such extensions in *B. porifera* and *B. oatesiana*). *Bispira secusoluta* differs from *B. brunnea* in having paired, large, lung-shaped ciliary pads on the collar segment and small, rounded patches of cilia in the shields of thoracic and abdominal segments (elliptic ciliary pads, and lacking patches of cilia in thoracic and abdominal segments in *B. brunnea*). The presence of paired patches of cilia in body segments has been only reported in *Pseudobranchiomma schizogenica* by Tovar-Hernández & Dean (2014), but probably these structures have been overlooked and are present in many species.

Among the currently known species of *Bispira* in the Indian Ocean, *B. secusoluta* and *B. porifera* have no radiolar eyes (Table 3). *Bispira porifera* can be distinguished by the presence of dorsal spongy cushions, absent in *B. secusoluta*.

***Bispira porifera* (Grube, 1878)**

(Figs. 6 and 7)

*Sabella porifera* Grube, 1878: 252, pl. 14, fig. 3; Ehlers, 1920: 69.

*Eurato porifera*.— Willey, 1905: 309, pl. 7, fig. 173.

*Bispira porifera*.— Knight-Jones & Perkins, 1998: 426–428, fig. 16; Capa, 2008: 307, 309, figs 2, 3, 4A–F; Capa & Murray, 2015a: 104, 106, fig. 2G–I.

**Material examined.** Indonesian-Dutch Snellius II Expedition, Sta.4.147A, NE Takabonerate (Tiger islands), western edge of reef Taka Garlarang, 06°27′S, 121°12.5′E, 1–2 m deep cave at 7 m depth, September 27, 1984, 1 specimen [RMNH.VER. 19932].

**Description.** Trunk 46 mm long, 8 mm wide. Radioles without eyes. Anterior peristomial ring purple, forming two rounded, prominent lobes (Figs. 6A–6C). Posterior peristomial ring collar with dorsal margins not fused to faecal groove. Two pairs of cushion-like masses dorsally, separated by mid-dorsal faecal groove: anterior pair purple, granular, extending around peristome (Figs. 6A and 6C); posterior pair pale, spongy, with many oval to circular-shaped pores, unequal in diameter (Figs. 6A and 6C), extended to third thoracic chaetiger. Ventral lappets of collar prominent, triangular, separated. Ventral sacs present outside radiolar crown, purple (Fig. 6B). Thorax with eight chaetigers. Narrow mat of yellow glandular tissue visible on dorsal side of posterior thoracic segments, and four anterior abdominal segments. Tori of chaetigers 1–5 in contact with ventral shields (tori occupy the entire distance between notopodia and ventral shield margin), tori in chaetigers 6–8 not contacting shields. Abdomen with 173 chaetigers. Interramal eyespots only visible in posterior abdomen, small, rounded spots. Tube amber, anteriorly covered by white sand.

**Histology.** The anterior pair of dorsal cushion-like masses, located on the anterior peristomial ring (Figs. 6A–6C), is a strongly innervated area (Figs. 6D–6G). Posterior pair of dorsal cushion-like masses is composed of a sinuous, wide glandular epithelium, 500 μm thick. Neural packages and glandular epithelium are surrounded by a dense cartilaginous matrix and muscular fibers, which run along thorax (Figs. 6D–6H).

**Figure 7:**
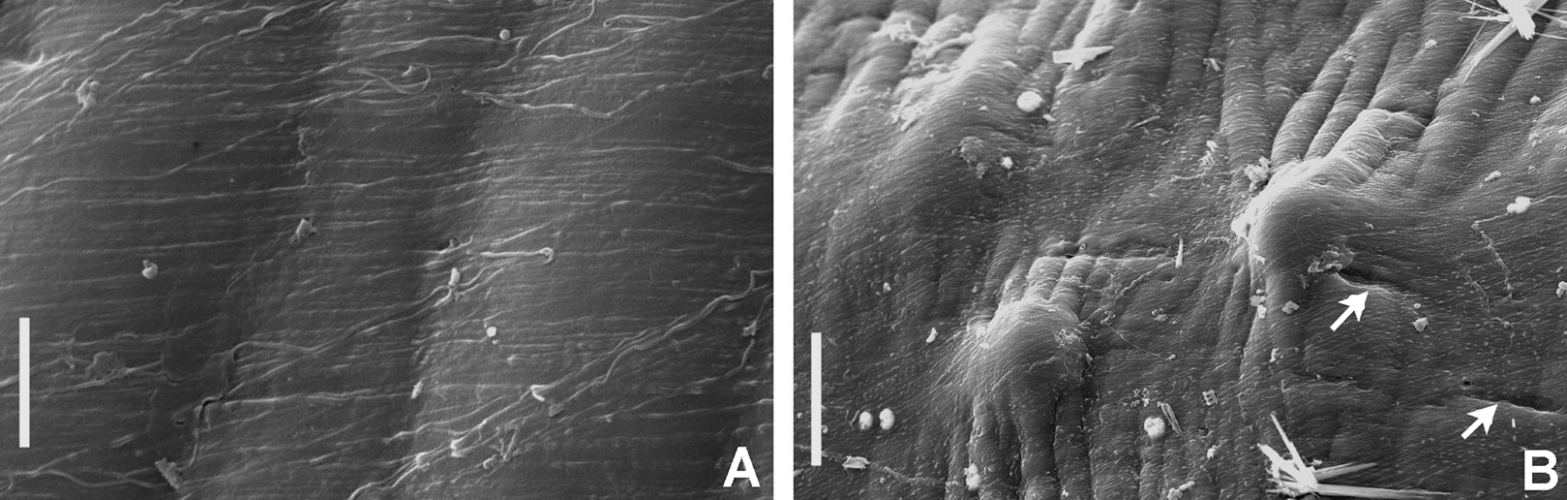
Tube microstructures of *Bispira porifera*. (A) Surface of lumen showing fibers with two alternate orientations, (B) microrelief on the surface of lumen showing small bumps and wrinkles. Scale bars: (A) 5 μm, (B) 20 μm. Pits are indicated with arrows.

**Tube microstructure.** The tube lumen relatively smooth, but in places with wrinkles, elongate pits and somewhat circular bumps. Two sets of fibers in alternate orientations at about 35–40° to each other. Fibers in single set moderately-developed, but long (>15 μm), usually straight and parallel to each other, but some fibers slightly undulating or curved. Some fibers with widened drop-like nodes at irregular intervals. Fibers 0.15–0.30 μm wide, interspaces usually wider than diameter of fibers. Interspaces of adjacent fibers filled with homogeneous organics with smooth surface. Tube wall dense, non-porous, except for rare elongate pits (1–4 μm long) oriented parallel to the fibers (Figs. 7A and 7B).

**Remarks.** The specimen here reported matches the descriptions by Willey (1905); Knight-Jones & Perkins (1998); Capa (2008) and Capa & Murray (2015a). *Bispira porifera* is known from Bohol, Philippines (type locality); India, Sri Lanka, Andaman Islands, Zanzibar, Madagascar (Knight-Jones & Perkins, 1998); Northern Territory and Western Australia (Capa, 2008) and Takabonerate, Indonesia (our study).

Spongy dorsal cushions have been reported only in two species of *Bispira*: *B. porifera* (Grube, 1878) and *B. paraporifera* Tovar-Hernández & Salazar Vallejo, 2006. Both species have been found associated with dead coral blocks, but there is no information about structure and function of this peculiar tissue. Willey (1905) correctly suggested that spongy cushions of *B. porifera* are glandular, but this only applies to the second pair of cushions, consisting of glandular columnar epithelium. The anterior pair of cushions, located above peristomium, is not glandular, but strongly innervated, not unexpectedly so since the brain ganglion is located in that area. Types of glandular cells were not determined, but their function might be associated with mucous secretion for tube construction or re-construction of damaged parts. Knight-Jones & Perkins (1998) suggested that this tissue might have a function in embryo incubation, but there is no evidence to support this hypothesis. Information of reproduction in *B. porifera* is null, but its congener *B. brunnea* (Treadwell, 1917), is a Caribbean broadcast species with sperm morphology adapted to external fertilization in the water column (Dávila-Jiménez, Tovar-Hernández & Simões, 2017).

**Genus *Branchiomma*Kölliker, 1858** (p. 537)

*Branchiomma*.— Johansson, 1927: 158; Day, 1967: 767; Fauchald, 1977a: 138; Fitzhugh, 1989: 73–74; Nogueira, Rossi & López, 2006: 597; Capa et al., 2019: 192–193.

*Dasychone* Sars, 1862: 118.— *fide* Rioja, 1923: 41; Johansson, 1927: 158; Hartman, 1959: 540; Fauchald, 1977a: 140.

*Dasychonopsis* Bush, 1905: 198.— *fide* Johansson, 1927: 158; Hartman, 1959: 541; Fauchald, 1977a: 140.

**Type species**: *Amphitrite bombyx* Dalyell, 1853, by monotypy.

**Number of species**: 29, after Keppel, Tovar-Hernández & Ruiz (2015) and Keppel, Tovar-Hernández & Ruiz (2018).

**Remarks.** The genus *Branchiomma* is large and taxonomically complex. Diagnoses to genus level are available in Fitzhugh (1989), Knight-Jones (1994), Nogueira, Rossi & López (2006), Tovar-Hernández & Knight-Jones (2006) and Capa et al. (2019). The World Register of Marine species (WoRMS) lists 31 species (Read & Fauchald, 2020a).

*Branchiomma* is unique by the presence of paired stylodes (epithelial flaps) along the outer surface of the radiolar axes, an autapomorphy (Capa et al., 2019). Proper identification is particularly challenging for this genus because there is morphological variation in taxonomically informative characters at species level (Keppel, Tovar-Hernández & Ruiz, 2018). As a result, the nomenclature of the genus is in a state of flux and it is currently under review using molecular identification techniques (del Pasqua et al., 2018; Belato et al., 2018). *Branchiomma* is among the most visible polychaetes of the hard substrate fouling communities, and several species have been reported outside their naturally expected distribution ranges (Tovar-Hernández, Méndez & Salgado-Barragán, 2009; Keppel, Tovar-Hernández & Ruiz, 2015). The most recent account of alien *Branchiomma* includes eight species (Keppel, Tovar-Hernández & Ruiz, 2015; Keppel, Tovar-Hernández & Ruiz, 2018). Cases of high phenotypic plasticity in taxa from Australia and Mediterranean Sea, and probably all around the world, high infraspecific genetic variability, cryptic species and unexpected translocations of species beyond original distributions were documented by Capa, Pons & Hutchings (2013) and del Pasqua et al. (2018).

***Branchiomma boholense* (Grube, 1878)**

*Sabella* (*Dasychone*) *boholensis* Grube, 1878: 261–262; Wiktor, 1980: 280, 3 syntypes in Museum of Natural History, Wrocław University, MPW 365 (Grube mentions (p. 262) 2 specimens, of which the second with tube. Possibly this led to the three syntypes –2 in formalin, 1 dry– mentioned by Wiktor).

*Branchiomma boholense*.— Knight-Jones, Knight-Jones & Ergen, 1991: 852–854, fig. 6P; Román, Pérez-Ruzafa & López, 2009: 244–248, figs 2–3 (*partim*); Cepeda & Rodríguez-Flores, 2017: 5–7, figs 1E–H, 3; del Pasqua et al., 2018: 12, fig. 10.

**Material examined.** Indonesian-Dutch Snellius II Expedition, Sta. 4016, Tukang Besi islands, Banda Sea, Kaledupa reef, E of entrance, 5°56′S, 123°48′E, gently sloping reef, 1–8 m depth, legit J.C. den Hartog, September 8, 1984, 1 specimen [RMNH.VER. 19934]. Sta. 4.051, NE coast of Sumba, E of Melolo, 9°53.5′S, 120°42.7′E, 75–90 m depth, calcareous stones, rich epifauna dominated by soft corals, rectangular dredge, September 13, 1984, 2 specimens [RMNH.VER. 19935]. Sta. 4096A, Komodo, NE cape, 8°29′S, 119°34.1′E, edge of narrow coastal reef, September 19–20, 1984, reef patches in sand, 3 m depth, 1 specimen [RMNH.VER. 19936]. Sta. 4097, Komodo, NE cape, 8°29′S, 119°34.1′E, littoral zone, rocks adjacent to sandy shore, September 19–20, 1984, 2 specimens [RMNH.VER. 19937]. Sta. 4.099, E of Komodo, 8°29′S, 119°38.2′E, rectangular dredge, 81 m depth, small calcareous nodules, echinoderms, sponges, September 19, 1984, 1 specimen [RMNH.VER. 19938].

**Description.** Trunk 10–14 mm long (adults), 2.5–3.3 mm (juveniles); width 3–4 mm (adults), 0.4–0.7 (juveniles). Length of radiolar crown 8 mm (adults), 1.2–2.5 mm (juveniles). Radioles: 17–20 pairs (adults), 6–7 pairs (juveniles). One to four pairs of flattened, tongue-like macrostylodes per radiole, 2–3 times longer than microstylodes, alternating randomly and mostly along distal crown half. Basal stylodes unpaired, on all radioles: located on left side of rachis on right branchial lobe (dorsal view), and on right side of rachis on left lobe (dorsal view). Radiolar tips filiform, long, about as long as section with 10–13 pinnules (adults), 5–6 (juveniles). Radiolar eyes mostly oval, some nearly circular. Thorax with eight chaetigers (adults), 4–5 (juveniles). Thoracic uncini with 2–3 rows of teeth. Abdomen with 49–71 segments (adults), 13–16 (juveniles). Abdominal neuropodia composed of C-shaped, compact tufts of spine-like chaetae surrounding a central bundle of modified elongate narrowly hooded chaetae. Simultaneous hermaphrodites, gametes present from posterior thoracic segments to end of abdomen.

**Remarks.** *Branchiomma boholense* was originally described from Bohol islands (Philippines). It has been reported from the Eastern Mediterranean (Malta, Atlit, Tel-Aviv and Alexandria) by Knight-Jones, Knight-Jones & Ergen (1991); Cyprus by Çinar (2005) but then corrected to *B. bairdi* (Çinar, 2009); from the Western Mediterranean (SE coast of Spain) by Román, Pérez-Ruzafa & López (2009); there are other records from Hong Kong and Sri Lanka by Knight-Jones, Knight-Jones & Ergen (1991) that should be checked against present knowledge.

Cepeda & Rodríguez-Flores (2017) redescribed *Branchiomma boholense*, based on the examination of type material. In their opinion the material reported by Knight-Jones, Knight-Jones & Ergen (1991) might belong to either *B. boholense* or *B. bairdi*, and should be re-identified; records of *B. boholense* by Román, Pérez-Ruzafa & López (2009) would belong to *B. bairdi*. However, Román, Pérez-Ruzafa & López (2009) examined a large number of specimens (over 2000), and after their illustrations and description, at least some of their specimens might be *B. bairdi*, some other *B. boholense*. The most distinctive feature in *B. boholense* is the presence of flattened, tongue-like macrostylodes. Román, Pérez-Ruzafa & López (2009) figure 2D shows a radiole with a strap-like macrostylode (as in *B. bairdi*), whereas their figure 3B gives a radiole with flattened, tongue-like macrostylodes (as in *B. boholense*). The presence of both species, *B. bairdi* and *B. boholense* in the Mediterranean Sea was confirmed recently by del Pasqua et al. (2018) using molecular and morphological evidence.

Specimens from Sumba here reported (RMNH.VER. 19935) were illustrated in del Pasqua et al. (2018): figure 10. These specimens agree with the description provided by Cepeda & Rodríguez-Flores (2017), except for the presence of paired basal stylodes and short, blunt radiolar tips, occupying the space of 3–5 pinnules. Specimens examined here, all juvenile and adult stages, present unpaired basal stylodes and long, filiform radiolar tips (occupying the space of approximately 10–13 pinnules in adults, 5–6 in juveniles).

Capa, Pons & Hutchings (2013) already emphasized the presence of basal stylodes being paired in some few taxa (*B. lucullanum*, *B. bombyx* and *B. galei*) and showing plasticity in the majority of the studied species: specimens within the same species were found with single or paired basal stylodes. However, a fixed relation of presence–absence of this feature with juvenile or adult stages, or regenerating worms has not been observed.

Female and male gametes were found together within the coelomic thoracic and abdominal cavity of *B. boholense* from Indonesia. As other invasive congeners such as *B. bairdi*, *B. coheni* and *B. luctuosum*, *B. boholense* is a simultaneous hermaphrodite (Licciano, Giangrande & Gambi, 2002; Tovar-Hernández, Méndez & Salgado-Barragán, 2009; Tovar-Hernández, Yáñez-Rivera & Bortolini-Rosales, 2011; Lezzi et al., 2016; del Pasqua et al., 2017).

Two species of *Branchiomma* were described by Grube from the Central Indo-Pacific: *B. cingulatum* (Grube, 1871) from Fiji and *B. boholense* (Grube, 1878) from Bohol Islands, Philippines. Both can be distinguished by the absence of macrostylodes in *B. cingulatum* and the presence of tongue-like macrostylodes in *B. boholense. Branchiomma cingulatum* was also reported from Ambon by Augener (1933, as *Dasychone*).

**Genus *Claviramus*Fitzhugh, 2002** (pp 412, 414–415), emendation

*Claviramus*.— Capa et al., 2019: 193–194.

**Type species**: *Sabella candela* Grube, 1863, by original designation.

**Number of species**: 5, including one new species described below.

Radioles in semi-circular radiolar lobes, each radiole with two rows of vacuolated cells. Palmate membrane absent; radiolar tips with foliaceous flanges, expanded or curled; basal flanges absent; radiolar eyes absent. Dorsal lips with radiolar appendages, pinnular appendages apparently absent; ventral radiolar appendages present, few to several pairs. Ventral lips present, ventral sacs and parallel lamellae absent. Anterior peristomial ring with broad, triangular, ventral lobe. Posterior peristomial ring collar with wide mid-dorsal gap, mid-ventral incision and ventral lappets. Peristomial vascular loops present in some species. Peristomial eyespots may be present. Glandular ridge on chaetiger 2 present or not. Ventral shields present on thorax; present or absent on abdomen. Interramal eyespots absent. Collar chaetae similar to superior notochaetae of following chaetigers, elongate, narrowly hooded; inferior thoracic notochaetae broadly hooded, or narrowly and broadly hooded. Thoracic uncini acicular, with short teeth above main fang arranged on transverse rows, hood present, handle long, main fang may be bifid. Neuropodial companion chaetae absent. Abdominal uncini avicular, with distinctly short handle, developed squared to rectangular breast and several transverse rows of short teeth above main fang. Abdominal neurochaetae in single group of narrowly hooded chaetae. Pygidium with eyespots present in at least some species. Anal cirrus absent.

**Remarks.** *Claviramus* Fitzhugh, 2002 currently includes five species worldwide. *Claviramus candelus* (Grube, 1863), the type species of the genus, was originally described as *Sabella candela* Grube, 1863, from the Northern Adriatic Sea. Langerhans (1884) transferred the species to *Jasmineira* Langerhans, 1880, and described a new species *J. oculata* Langerhans, 1884, from Madeira. Cochrane (2000) redescribed those species within *Jasmineira* in detail, based on type and additional specimens. Fitzhugh established the genus *Claviramus*, based on the presence of radiolar tips with foliaceous flanges (Fitzhugh, 2002: fig. 43) and transferred *J. candela* and *J. oculata* to *Claviramus*. The third species, *C. grubei* Fitzhugh, 2002, was described from Thailand, Andaman Sea. *Claviramus kyushuensis* Nishi, Tanaka & Tovar-Hernández, 2019 was described from Japan and in the present study, the fifth species is described below from Indonesia.

The generic diagnosis above primarily follows Fitzhugh (2002). The emendation provided here is to include the presence of (1) peristomial vascular loops present in *Claviramus kyushuensis* from Japan (Nishi, Tanaka & Tovar-Hernández, 2019) and the new species described below from Bay of Sanggar, (2) the presence of bifid main fangs of thoracic uncini reported in *C. kyushuensis*, and (3) presence of abdominal shields in *C. kyushuensis* as well as the new species described below.

***Claviramus*** ***olivager* sp. nov. Tovar-Hernández, ten Hove & García-Garza**

(Fig. 8)

**Figure 8:**
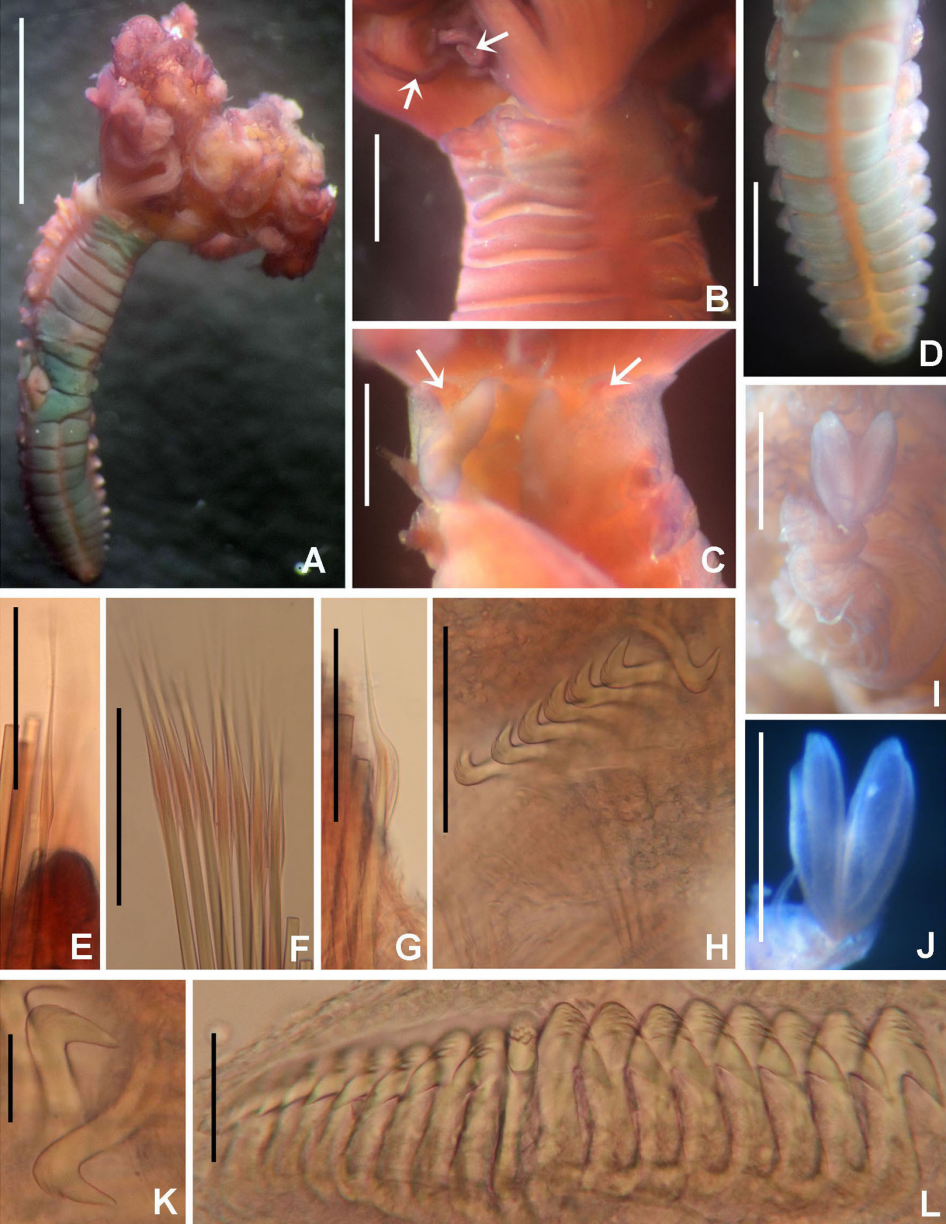
*Claviramus olivager* sp. nov. (A) Body, ventral view, (B) collar, ventral view, and ventral radiolar appendages, indicated by arrows, (C) collar, dorsal view, and vascular loops, indicated by arrows, (D) abdominal shields, (E) narrowly hooded chaeta from collar, (F) superior thoracic chaetae, narrowly hooded, (G) inferior thoracic chaetae, broadly hooded, (H) thoracic acicular uncini, (I–J) foliaceous, curled radiolar tips, (K) heads of thoracic uncini, (L) abdominal uncini. Scale bars: (A) 2 mm, (B–D, I–J) 0.5 mm, (E–H) 30 μm, (K) 10 μm, (L) 20 μm. Stain: (A–D) methyl green. Holotype, RMNH.VER. 19939.

LSID: urn:lsid:zoobank.org:act:F8BA6972-4240-4042-A7DF-FC089AC7A2D2

**Material examined.** Holotype [RMNH.VER.19939]: Indonesian-Dutch Snellius II Expedition, Sta. 4.114, N of Sumbawa, Bay of Sanggar, 8°19.2′S, 118°14.4′E, lagoon side of reef barrier, 18–20 m depth, September 21–22, 1984.

**Comparative material.** Paratypes [UANL 8130] *Claviramus kyushuensis* Nishi, Tanaka & Tovar-Hernández, 2019. Ariake Sound, Kyushu, Japan, Stn 20D, 20 m depth, sandy mud bottoms, collected by dredge by K. Mori, 17 September, 2005, 3 specimens.

**Description.** *Colour, body shape, and size*. Body cream coloured. Trunk cylindrical, posterior abdomen depressed (Fig. 8A). Trunk 3.8 mm long; 1.5 mm wide.

*Radiolar crown*. Length 2.4 mm. Radiolar lobes semi-circular. Eight pairs of radioles. Five pairs of ventral radiolar appendages (Fig. 8B), of different lengths (from 1/4 to 1/2 as long as radiolar crown). Palmate membrane absent. Radiolar flanges only present on radiolar tips. Radiolar tips with foliaceous curled flanges, with a mid-ventral incision for half their length (Figs. 8I and 8J). Pinnules absent between distal flanges. Radiolar eyes absent. Excessive handling of internal radiolar crown appendages was avoided; dorsal lips elongated, ventral lips shorter.

*Peristomium*. Anterior peristomial ring with ventral margin as broadly triangular lobe, not extending beyond collar margins. Posterior peristomial ring collar with ventral margin with a shallow mid-ventral incision forming two discrete rounded lappets (Fig. 8B). Dorso-lateral margins fused to faecal groove (Fig. 8C). Dorsal pockets present. Large vascular loops visible on dorsal pockets of collar (Fig. 8C). Lateral collar margins slightly oblique, with ventral margin slightly higher than dorsal.

*Thorax*. Chaetiger 1: with rows of narrowly hooded chaetae (Fig. 8E). Collar shield divided transversally into three nearly rectangular sections (Fig. 8B); ventral side of collar with one pair of white triangular glandular pads, lung-shaped. Chaetigers 2–8: tori not contacting ventral shields (Fig. 8B). Notochaetal superior group with narrowly hooded chaetae (Fig. 8F) and two inferior rows of broadly hooded chaetae (Fig. 8G). Neuropodial uncini acicular (Fig. 8H); main fang surmounted by several rows of very small teeth occupying half of main fang length (Fig. 8K), tip of main fang entire in frontal view, breast narrow swelling; handles very long. Ventral shields well developed (Figs. 8A and 8D). Shield of chaetiger 2 divided transversally into two nearly rectangular sections (Fig. 8B). Shields from chaetigers 3 to 8 rectangular, broad, entire (Figs. 8A and 8B). Glandular ridge on chaetiger 2 absent.

*Abdomen*. Segments: 10 (Fig. 8A). Neuropodial fascicles with 1–2 transverse rows of narrowly hooded chaetae. Notopodia with avicular uncini (Fig. 8L); main fang surmounted by 3–4 rows of small teeth equal sized, occupying half of main fang length; breast well developed; handles very short. Shields forming two squares divided by faecal groove (Fig. 8D). Pygidium rounded, without eyespots or cirrus (Fig. 8D).

*Tubes*. Not preserved.

*Sex and gametes*: Holotype female with asynchronous oocytes floating free in coelom of thorax and abdomen.

**Remarks.** *Claviramus olivager* sp. nov., is a new species based on a set of unique, distinctive features: absence of glandular ridge on chaetiger 2, distal radiolar flanges without filament, and a mid-ventral incision extending for half flange length, abdominal shields well developed, and ventral lobe of anterior peristomial ring not extending beyond collar margins (Table 4).

**Table 4:** Species of *Claviramus* from the world (after Nishi, Tanaka & Tovar-Hernández, 2019).

Species name	Glandular ridge on chaetiger 2	Abdominal glandular shields	Mid-ventral incision of distal radiolar flanges	Ventral margin of collar	Ventral shield of collar	Main fang of thoracic uncini	Pygidial eyes	Type locality
*Claviramus candelus* (Grube, 1863)	Absent	Present	? (Short, less than 1/4 of the flange length, *fide* Langerhans)	Even in height	Rectangular, entire	?	Present	Adriatic Sea
*Claviramus grubei* Fitzhugh, 2002	Present	Absent	Short, less than 1/4 of flange length	With shallow mid-ventral incision	?	?	Absent	Thailand, Phuket Island
*Claviramus oculatus* (Langerhans, 1884)	Absent	Absent	Short, less than 1/4 of flange length	With shallow mid-ventral incision	Rectangular, divided transversally into 2 areas (superior wider than inferior one)	?	Present	Madeira
*Claviramus kyushuensis* Nishi, Tanaka & Tovar-Hernández, 2019	Present	Present	Medium, 1/2 of flange length	With shallow mid-ventral incision	Rectangular, divided transversally into 3 nearly equal sized sections with lateral margins indented	Bifid in frontal view	Absent	Ariake Sound, Kyushu, Japan
*Claviramus olivager* sp. nov.	Absent	Present	Medium, 1/2 of flange length	With shallow mid-ventral incision	Rectangular, divided transversally into two nearly rectangular sections	Entire	Absent	Bay of Sanggar, Indonesia

*Claviramus candelus* and *C*. *olivager* sp. nov., have glandular abdominal shields and both lack a glandular ridge on chaetiger 2, but ventral margin of collar is entire, of a constant height in *C. candelus*, whereas it is incised, forming two slightly elevated lappets in *C*. *olivager* sp. nov. *Claviramus grubei* and *C. oculatus* have a distal filament (cirrus) on their radiolar tips (absent in *C*. *olivager* sp. nov.), and the ventral lobe of anterior peristomial ring is broadly triangular, extending slightly beyond collar margin (not exposed in *C*. *olivager* sp. nov.). Both taxa have a short mid-ventral incision of distal radiolar flanges, less than 1/4 as long as flange length while it is 1/2 of flange length in *C. olivager* sp. nov. (Table 4).

Nishi, Tanaka & Tovar-Hernández (2019) described a new species of *Claviramus* from Ariake sound (Japan): *C. kyushuensis*. In our study, we had opportunity to examine three of their paratypes. These specimens are hermaphrodites with oocytes and sperm in thoracic and abdominal segments. The holotype of *Claviramus olivager* sp. nov., is a female with oocytes in thorax and abdomen, but an exhaustive search of spermatozoa was not assessed, so we cannot exclude simultaneous hermaphroditism. *Claviramus olivager* sp. nov., differs from *C. kyushuensis* from Japan in lacking a glandular ridge on chaetiger 2 (present in *C. kyushuensis*) and having tips of main fang of thoracic uncini entire (bifid in *C. kyushuensis*).

Distal flanges are very fragile and easily broken off from radioles during manipulation. Cochrane (2000) also showed broken radioles in some specimens belonging to *C. candelus* (as *Jasmineira*). Under this scenario, it is feasible that more specimens still erroneously can be found under *Jasmineira*. However, *Jasmineira* and *Claviramus* might also be distinguished on the basis of the presence of inferior thoracic bayonet notochaetae (absent in *Claviramus*), uncinial morphology and presence of a breaking plane *sensu* Cochrane (2003) or abscission zone *sensu* Tovar-Hernández (2008).

**Etymology.** The specific name refers to the shape of radiolar tips, that resembles the elongate oval-shaped shells of the mollusk genus *Oliva* Bruguière, 1789.

**Genus *Notaulax*Tauber, 1879** (p. 136), emendation

*Notaulax*.— Perkins, 1984: 327, 329; Fitzhugh, 1989: 75; Tovar-Hernández, de León-González & Bybee, 2017: 21; Capa et al., 2019: 197–198.

**Type species**: *Notaulax rectangulata* Levinsen, 1884, by subsequent designation (ICZN, 1999, Art. 69.1).

**Number of species**: 24, after Nishi et al. (2017), Tovar-Hernández, de León-González & Bybee (2017), and including one new species described below.

**Description.** Radioles in semi-circular radiolar lobes, each radiole with at least four rows of vacuolated cells. Radiolar crown with elongate basal lobes; palmate membrane, radiolar flanges, and dorsal and ventral basal flanges present. Numerous ocelli arranged in longitudinal rows on lateral sides of radioles. Dorsal lips with radiolar appendages, pinnular appendages absent; ventral radiolar appendages absent. Ventral lips and parallel lamellae present, ventral sacs inside radiolar crown. Anterior peristomial ring low, of even height, or high and rounded. Posterior peristomial ring collar with narrow mid-dorsal gap, dorsal margins laterally fused or not to the faecal groove, ventrally entire or with mid-ventral incision and short ventral lappets. Peristomial vascular loops absent. Peristomial eyespots absent. Thorax and abdomen with variable number of segments. Glandular ridge on chaetiger 2 absent. Ventral shields present. Interramal eyespots may be present. Collar chaetae spine-like, shaped like oblique longitudinal rows, diagonal, J or C; superior thoracic notochaetae short spine-like, inferior thoracic notochaetae paleate. Thoracic uncini avicular, with several rows of minute and similar in size teeth above main fang, developed breast and medium-sized handle; neuropodial companion chaetae with strongly asymmetrical hood stouter on one margin and thin, elongate tip. Abdominal uncini similar to the thoracic ones. Anterior abdomen with a superior group of elongate, narrowly hooded chaetae and an inferior group of paleate chaetae with mucros. Posterior abdomen with modified, elongate, narrowly hooded chaetae, and paleate chaetae (spherical or oval) with long mucros. Pygidial eyespots may be present. Anal cirrus absent.

**Remarks.** Perkins (1984) revised the genus, described new species, provided several synonyms, and proposed new combinations of species within *Notaulax*. Later, a revisory contribution of members of the genus from Australia has been conducted by Capa & Murray (2015a); a second, from Mexico is in process by Tovar-Hernández. Diagnoses to genus level are available in Perkins (1984), Fitzhugh (1989) and Capa et al. (2019). The generic diagnosis was emended in order to include: (1) the presence of abdominal interramal eyespots (as reported in Tovar-Hernández, de León-González & Bybee (2017)); (2) the presence of a high, peristomial ring as seen in *N. pyrrohogaster* and *N. tenuitorques* (see below) and also in *N. bahamensis* Perkins, 1984 and *N. nudicollis* (Krøyer, 1856) (Perkins, 1984: figs 25E–F, 35E–F). (3) The difference between chaetae from anterior and posterior abdominal segments.

Seven species of *Notaulax* are known to occur embedded in coral masses (Nishi et al., 2017), one was found associated with a fossil reef; other species have been found fouling in marinas and ports (Tovar-Hernández, de León-González & Bybee, 2017), for others there is no information on the substrates from which they were collected (Nishi et al., 2017).

Two species of *Notaulax* from Australia went unnoticed as such in previous papers: *N. velata* (Haswell, 1885, as *Sabella*) from Port Jackson (Sydney) and *N. longithoracalis* (Hartmann-Schröder, 1980, as *Hypsicomus*) from Port Samson (Western Australia). Based on their original descriptions and drawings, both species have chaetae, collar shape and radiolar ocelli typical of *Notaulax*. Thus, *N. longithoracalis* was included in Table 5, whereas *N. velata* was left out because its original description is incomplete in critical features for species comparisons.

**Table 5:** Species of *Notaulax* from the Australia, Indonesia, Japan and Philippines.

Species name	Collar chaetiger	Radiolar ocelli	Collar	Ventral margin of collar	Dorsal margin of collar	Base of radiolar crown (lateral view)	Radiolar tips	Type locality
*N. longithoracalis* (Hartmann-Schröder, 1980)	Straight, oblique	4–6 ocelli in single row	Notched	Low, rounded, notched (but it may be folded, not real incision *fide* Hartmann-Schröder (1980)	Notched	As long as first 3 thoracic segments	Short (4–5 pinnules)	Port Samson, Western Australia
*N. pyrrhogaster* (Grube, 1878)	Slightly curved	28–30 ocelli in single row, from the mid-radiole length to distal pinnules. Basal ocelli well separated; distal eyes mutually close each from other	Entire all around	Long, triangular	Entire, not fused to faecal groove	As long as first 3 thoracic segments	Long (10–15 pinnules), flanged	Bohol, Phillipines
*N. tenuitorques* (Grube, 1878)	Straight, oblique	Groups of 20–26 ocelli at 3/4 of radiole length, then one row of 8–12 ocelli distally	Entire all around	Low, rounded	Entire, not fused to faecal groove	As long as first 3 thoracic segments	Long (15–18 pinnules), flanged	Bohol, Phillipines
*N. yamasui* Nishi et al., 2017	L-shaped orientation	8–12 ocelli in single row on each side, at lateral margin of central region of radioles	Incised	Slightly higher, triangular, with a short incision or notch	Entire, not fused to faecal groove	As long as first 3 thoracic segments	Short (5–6 pinnules), flanged with sub-distal swelling tips	Okinawa and Owasagara, Japan
*N*. sp. 1 (as in Capa & Murray, 2015a)	Strongly curved	15–20 ocelli in a single row, sometimes in a double row proximally	Entire or notched	Slightly higher, rounded, with a short incision or notch	Entire or notched	As long as thorax length or as long as 8 thoracic segments	Short (number of pinnulae cannot be inferred from paper), unflanged	Lizard Island, Australia
*N*. sp. 2 (as in Capa & Murray, 2015a)	J- or C-shaped	30 ocelli in a single row along radiole	Entire or notched	Slightly higher, triangular, entire or with a small mid-ventral incision	Entire or notched	As long as first 3 thoracic segments	Medium length (number of pinnulae cannot be inferred from paper), flanged	Lizard Island, Australia
*N*. sp. 3 (as in Capa & Murray, 2015a)	Straight, oblique	20 ocelli in teardrop-shaped groups on lateral margins of radioles	Incised	Long, triangular, with a short mid-ventral incision	Notched	As long as first 3 thoracic segments	Medium length (number of pinnulae cannot be inferred from paper), flanged	Lizard Island, Australia
*Notaulax montiporicola* sp. nov.	Straight, longitudinal	Absent	Incised	Long, with a long mid-ventral incision	Entire	As long as first 2 thoracic segments	Long (10–15 pinnules), unflanged	Tukang Besi Islands, Banda Sea

Capa & Murray (2015a) give the length of radiolar tips as short (*Notaulax* sp. 1) or medium (as long as a pinnule: *Notaulax* sp. 2, sp. 3). However, it is unknown if the pinnules considered by Capa & Murray (2015a) were distal, median or basal. As the length of pinnules in *Notaulax* increases from the radiolar base to the median radiolar zone, and then decreases gradually towards the tip, in our study the length of bare radiolar tips is expressed as the corresponding space of a given number of pinnules.

***Notaulax*** ***pyrrhogaster* (Grube, 1878)**

(Figs. 9 and 10)

**Figure 9:**
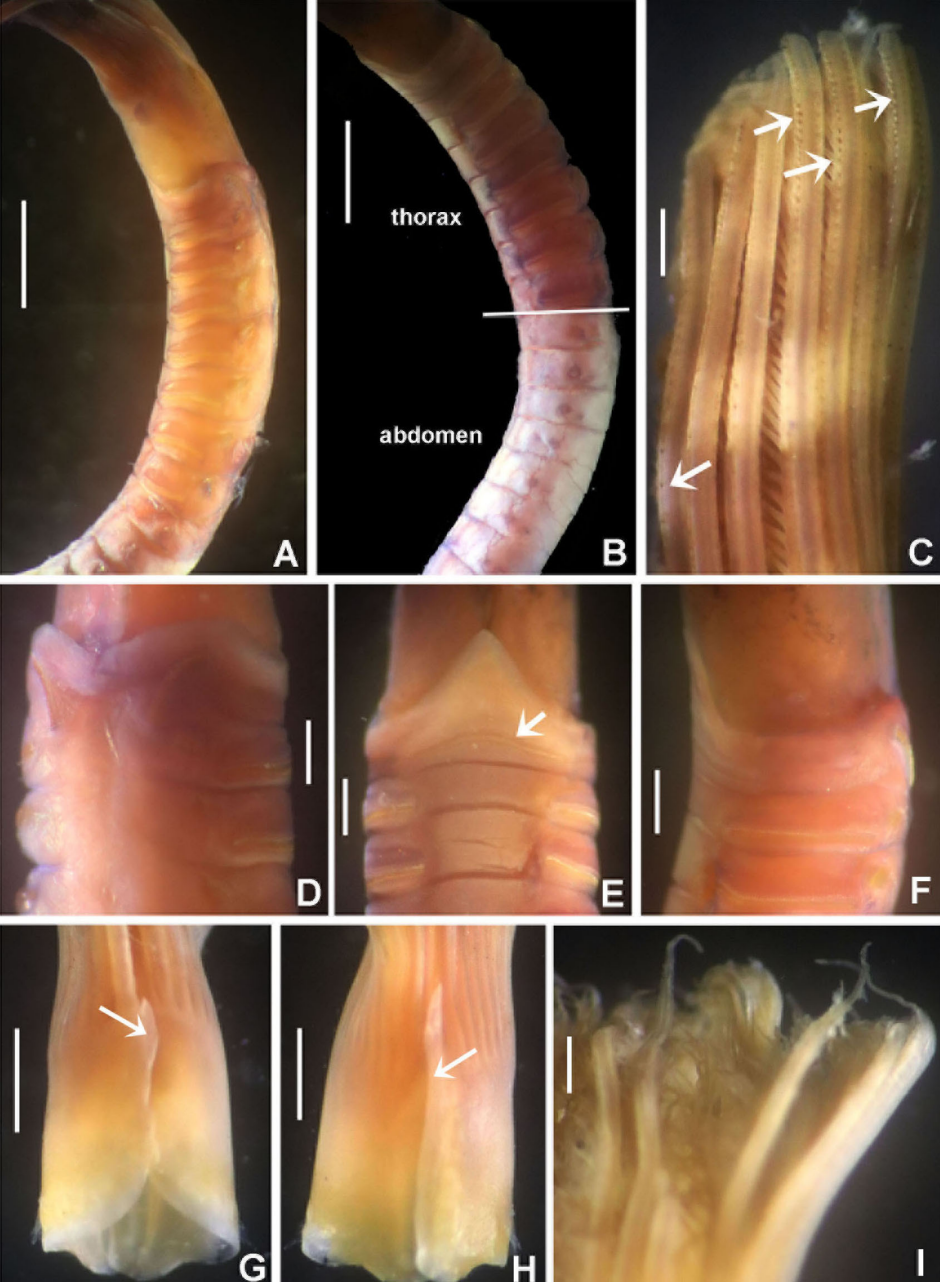
*Notaulax pyrrhogaster*. (A) Base of radiolar crown and thorax, lateral view, (B) chaetal inversion, (C) radiolar ocelli as pointed with arrows, (D) collar, dorsal view, (E) collar, ventral view, arrow indicating transversal line marking anterior margin of ventral shield, (F) collar, lateral view, (G) elongate base of radiolar crown, ventral side, arrow indicating the ventral flange, (H) same, dorsal side, arrow indicating the dorsal flange, (I) radiolar tips. Scale bars: (A and B) 1 mm, (C and I) 0.2 mm, (D–F) 0.4 mm, (G and H) 0.8 mm. Single specimen, RMNH.VER. 19940.

**Figure 10:**
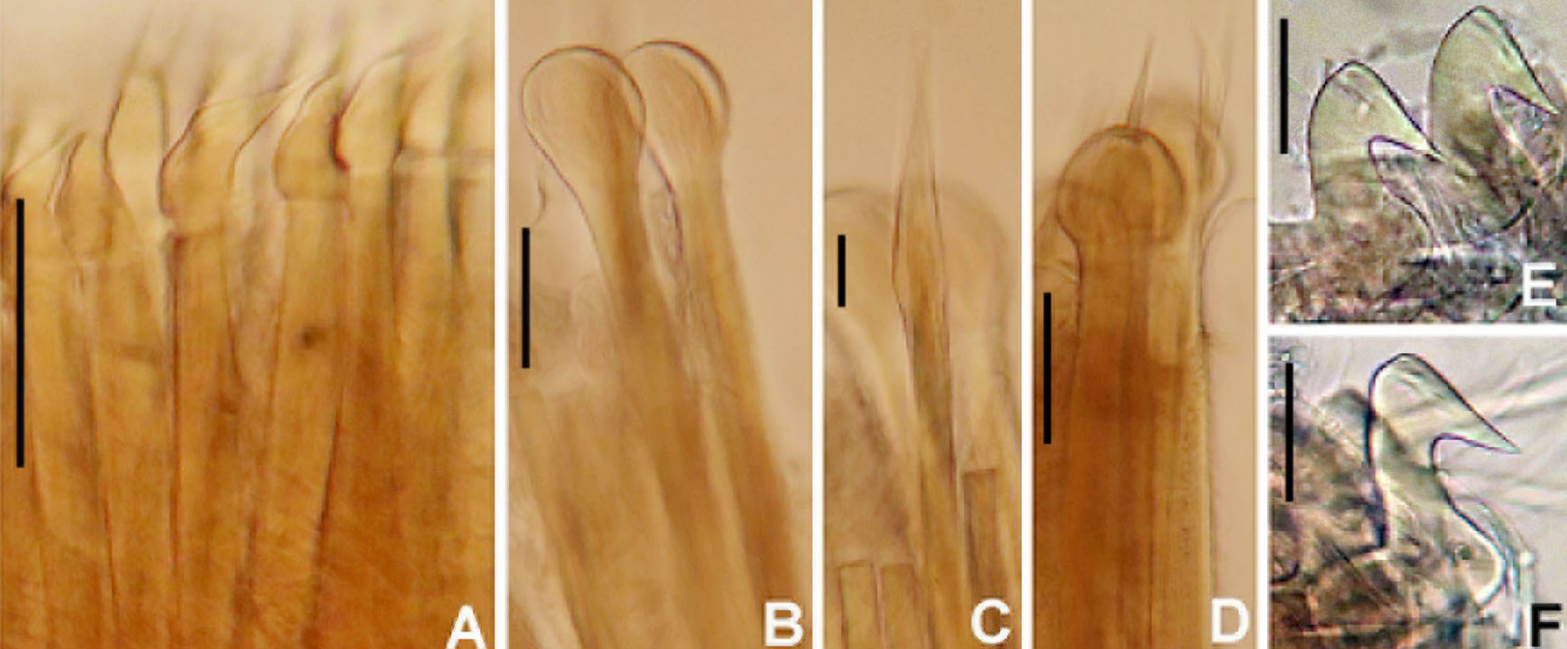
*Notaulax pyrrhogaster*: chaetae and uncini. (A) Spine-like chaetae from collar, (B) paleate chaetae from thorax, (C) broadly hooded chaeta from thorax, (D) paleate and narrowly hooded chaetae from abdomen, (E) thoracic uncini, (F) abdominal uncinus. Scale bars: (A) 130 μm, (B and D) 50 μm, (C) 20 um, (E and F) 60 μm. (A–F) RMNH.VER. 19940.

*Sabella pyrrhogaster* Grube, 1878: 250–252, pl. 15, fig. 1; Wiktor, 1980: 280, syntype in the Museum of Natural History, Wrocław University, MPW 370 (Grube (p. 250) mentions 2 specimens, the second specimen is either lost or overlooked by Wiktor).

*Eurato Pyrrhogaster.—* de Saint-Joseph, 1894: 249.

*Notaulax pyrrhogaster*.— Perkins, 1984: 328.

**Material examined.** Indonesian-Dutch Snellius II Expedition, Sta. 4.120B, Indonesia, N of Sumbawa, Bay of Sanggar, 08°20.5′S 118°15.7′E, nearly horizontal coastal reef, near seagrass, 1–3 m depth, September 23, 1984, 2 specimens [RMNH.VER. 19940].

**Description.** *Colour, body shape, and size*. Body cream coloured (Fig. 9A). All ventral shields from collar to posterior abdomen whitish (Fig. 9B). Trunk cylindrical, posterior abdomen depressed. Trunk 28 mm long, 1.4 mm wide.

*Radiolar crown*. Seven mm long. Eleven pairs of radioles arranged in two semi-circular lobes. Radiolar crown base 1.5 mm long, as long as first three thoracic segments in lateral view (Fig. 9A), with dorsal and ventral flanges (Figs. 9G and 9H). Radioles not inrolled mid-ventrally. Palmate membrane longer than base of radiolar crown. Outer margins of radioles flat narrow flanges (Figs. 9C and 9I). Radiolar tips flanged, long, filiform, 1 mm in length, equivalent space of 10–15 pinnules (Fig. 9I). Longest pinnules at 3/4 of radiolar length. Up to 28–30 ocelli arranged in a single row on each radiolar side (Fig. 9C). Basal ocelli widely spaced out; distal ocelli closer to each other, near the end of the radioles. Dorsal lips long, extending to end of palmate membrane, triangular with radiolar appendage. Ventral lips short, rounded lobes. Ventral and dorsal radiolar appendages absent.

*Peristomium*. Anterior peristomial ring not exposed beyond collar (not visible), high, rounded, slightly longer ventrally. Posterior peristomial ring collar entire all around (Figs. 9D–9F); ventral margin as long as 1/2 radiolar crown base, triangular, whitish (Fig. 9E); dorsal margin slightly convex, not fused to faecal groove (Fig. 9D).

*Thorax*. Chaetiger 1: with slightly curved rows of spine-like notochaetae (Figs. 9D and 10A); ventral shield narrow, rectangular with a brownish line on its anterior margin (Fig. 9E). Chaetigers 2–8: notopodia with superior broadly hooded chaetae (Fig. 10C), inferior paleate chaetae without mucros (Fig. 10B). Neurochaetae as avicular uncini (Fig. 10E), with medium-sized handles, developed breast and several rows of minute, similarly sized teeth occupying half of main fang; neuropodial companion chaetae with rounded denticulate head and long, gently tapering asymmetrical membrane. Ventral shields broad, quadrangular, nearly trapezoidal, laterally indented by neuropodial tori (Fig. 9E).

*Abdomen*. Segments: 137. Avicular abdominal uncini similar to thoracic ones, but handles shorter (Fig. 10F) and dentition covering 3/4 of main fang length; neuropodial tori with abdominal paleate neurochaetae with acicular mucros as long as paleal length (not including shaft) (Fig. 10D) and five needle-like chaetae, posterior to paleae (Fig. 10D), 1.5 times longer than paleae. Pygidium rounded with two black, large, reniform eyespots.

*Tubes*. Not preserved.

*Sex and gametes*. Unknown.

**Remarks.** de Saint-Joseph (1894) included *Sabella pyrrhogaster* in his new genus *Eurato*, and Bush (1905) subsequently designated it as the type species of the genus. According to Hartman (1959: 546) *Eurato* is a subjective synonym of *Hypsicomus* Grube, 1870. *Hypsicomus* has two pairs of accessory lamellae in the posterior peristomial ring, between the dorsal collar margins (absent in *Notaulax*), and collar chaetae in a typical short bundle (collar fascicles longitudinal to oblique in *Notaulax*). Based on these main differences, Perkins (1984) attributed *S. pyrrhogaster* to the genus *Notaulax*.

Grube (1878) stated specifically that ocelli were absent in *Notaulax pyrrhogaster* (as *Sabella*). His description indicates the largest specimen had damaged radiolar lobes. According to Perkins (1984) it is likely that radiolar ocelli were sloughed off with epidermal tissue, or faded. Our specimens have 28–30 radiolar ocelli per row, first appearing above mid-radiole length and continuing to distal pinnules. Basal radiolar ocelli are widely spaced out, whereas distal ocelli are very close to each other. The ventral and dorsal margins of the collar are as illustrated by Grube (1878: pl. XV, figs 1, 1a).

*Notaulax pyrrhogaster* and *N. tenuitorques* were both described from Bohol, Philippines. These species differ by the shape of the ventral margin of the collar, the arrangement of collar chaetae, and distribution of radiolar ocelli. The ventral margin of the collar is long, triangular in *N. pyrrhogaster* (low, rounded in *N. tenuitorques*). Collar chaetae are arranged in slightly curved rows in *N. pyrrhogaster* (in straight oblique rows in *N. tenuitorques*). *Notaulax pyrrhogaster* has radiolar ocelli in single rows from mid-radiole length to the distal pinnules (ocelli in groups of 15–17 ocelli at three quarters of radiole length, then one row of ocelli distally in *N. tenuitorques*).

*Notaulax pyrrhogaster* differs from other species from Australia and Japan in having a single row of 28–30 ocelli (from the mid-radiole length to distal pinnules), and a long, entire, triangular ventral collar margin (Table 5).

***Notaulax tenuitorques* (Grube, 1878), new combination**

(Figs. 11 and 12)

**Figure 11:**
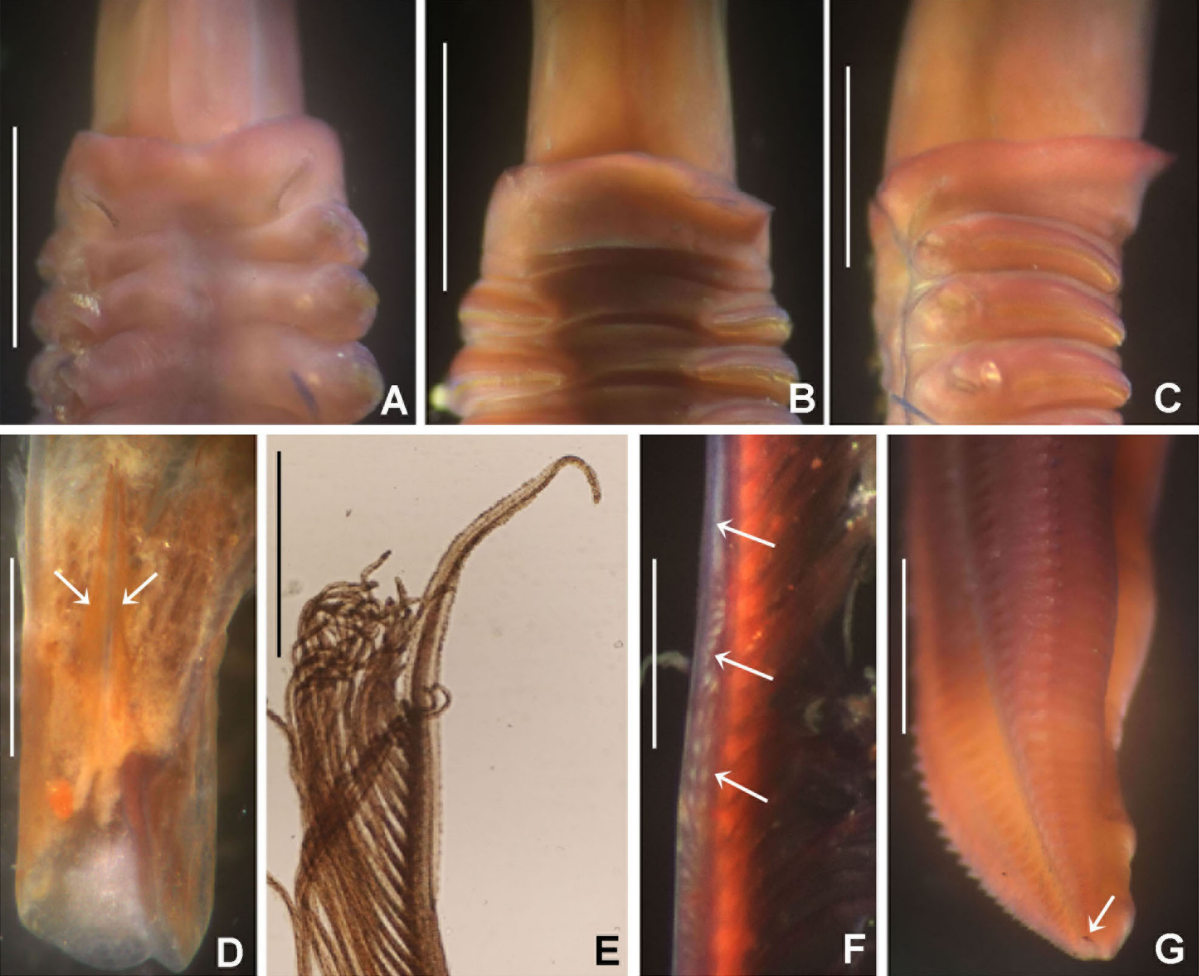
*Notaulax tenuitorques*. (A) Collar and first thoracic chaetigers, dorsal view, (B) same, ventral view, (C) same, lateral view, (D) dorsal lips indicated by arrows, (E) radiolar tip, (F) radiolar ocelli indicated by arrows, (G) posterior abdomen and pygidial eye indicated by arrow. Scale bars: (A–C) 1.5 mm, (D–E, G) 1 mm, (F) 0.3 mm. Stain: (A–C, F–G) shirla. Single specimens, RMNH.VER. 19941.

**Figure 12:**
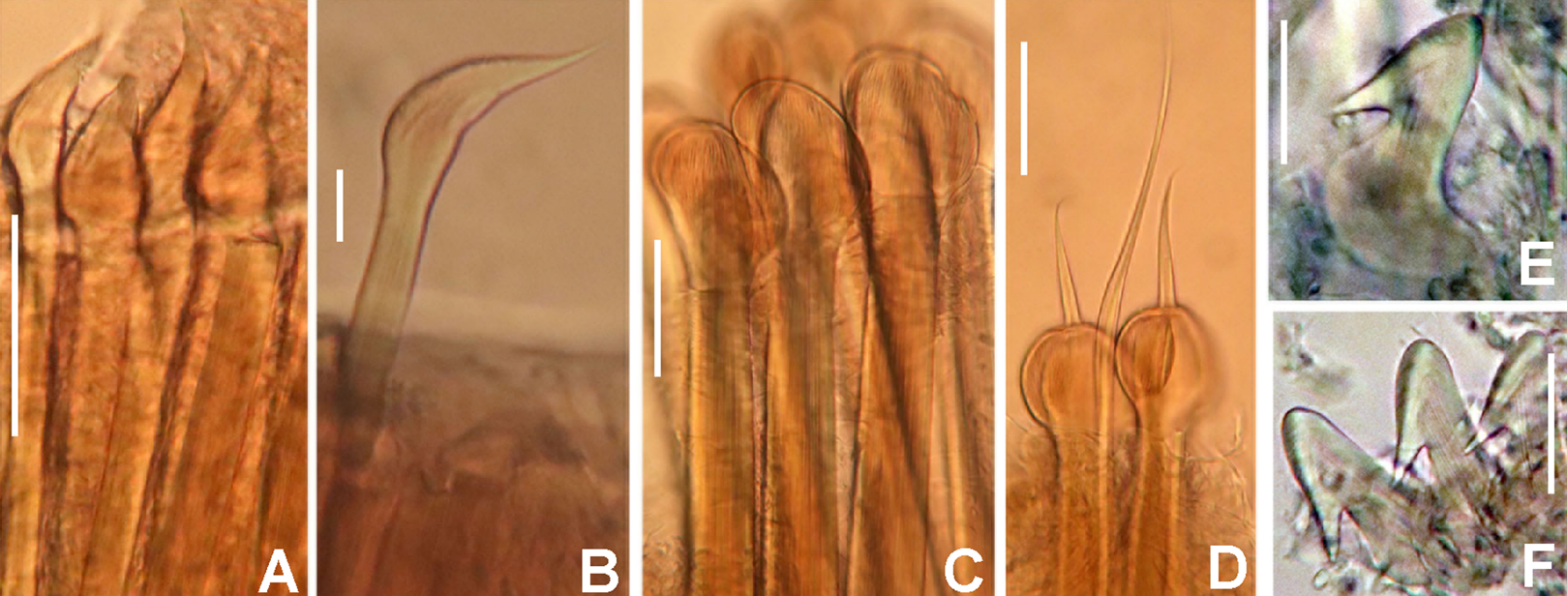
*Notaulax tenuitorques*: chaetae and uncini. (A and B) spine-like chaetae from collar, (C) paleate chaetae from thorax, (D) paleate and elongate, narrowly hooded chaetae from abdomen, (E) thoracic uncini, (F) abdominal uncinus. Scale bars: (A) 130 μm, (C and D) 50 μm, (B) 20 um, (E and F) 60 μm. (A–F) RMNH.VER. 19941.

*Sabella* (*Potamilla*) *tenuitorques* Grube, 1878: 246–247, pl. 14, fig. 2; Wiktor, 1980: 281, holotype in Museum of Natural History, Wrocław University, MPW 378; Hartwich, 1993: 142, 1 syntype? in Berlin, ZMB 1547 (see “Remarks”),

*Potamilla tenuitorques*.— Ehlers, 1920: 70; Hartman, 1959: 556.

**Material examined.** Indonesian-Dutch Snellius II Expedition, Sta. 4.120B, Indonesia, N of Sumbawa, Bay of Sangaar, 08°20.5′S 118°15.7′E, nearly horizontal coastal reef, near seagrass, 1–3 m depth, September 23, 1984, 2 specimens [RMNH.VER. 19941].

**Description.** *Colour, body shape, and size*. Body cream coloured. All ventral shields from collar to posterior abdomen brownish. Trunk cylindrical, posterior abdomen depressed. Trunk 38 mm long, wide 2 mm.

*Radiolar crown*. Nine mm long. Fourteen pairs of radioles. Radiolar lobes semi-circular. Elongate base of radiolar crown, 2 mm long, as long as first three thoracic segments in lateral view, with flanges: dorsal ones prominent (triangular in side view), ventral flanges reduced. Radioles not inrolled mid-ventrally. Palmate membrane as long as base of radiolar crown. Radioles with narrow flanges. Radiolar tips flanged, filiform, 0.8 mm long, or as long as 15–18 pinnules (Fig. 11E). Longest pinnules located at 3/4 of radiolar length. Radiolar ocelli located at 3/4 of the radiole length: basal ocelli arranged in groups of 20–26 ocelli, then one row of 8–12 ocelli distally (Fig. 11F). Dorsal lips long (Fig. 11D), extending to end of palmate membrane, triangular, with radiolar appendage. Ventral lips short, rounded lobes. Ventral and dorsal radiolar appendages absent.

*Peristomium*. Anterior peristomial ring not exposed beyond collar (not visible), high, rounded, slightly longer ventrally. Posterior peristomial ring collar entire all around; ventral margin low, rounded (Fig. 11B); dorsal margin slightly convex, not fused to faecal groove (Fig. 11A); lateral margin slightly higher ventrally (Fig. 11C).

*Thorax*. Chaetiger 1: with straight oblique rows of spine-like notochaetae (Figs. 11A, 12A, and 12B), ventral shield narrow, rectangular. Chaetigers 2–8: notochaetae with superior broadly hooded chaetae, inferior paleate chaetae without mucros (Fig. 12C). Neurochaetae avicular uncini (Fig. 12E), with medium-sized handles, breast developed and several rows of minute, similarly sized teeth occupying half of main fang; neuropodial companion chaetae with rounded denticulate head and long, gently tapering asymmetrical membrane. Ventral shields broad, quadrangular, nearly trapezoidal, laterally indented by neuropodial tori.

*Abdomen*. Segments: 127. Avicular abdominal uncini similar to thoracic ones but handles shorter (Fig. 12F), dentition covering 3/4 of main fang length; neuropodial tori with abdominal paleate neurochaetae with acicular mucros as long as paleal length (not including shaft) (Fig. 12D), and elongate, narrowly hooded chaetae, posterior to paleae (Fig. 12D), 1.5 times longer than paleae. Pygidium rounded with two black, large, reniform eyespots (Fig. 11G).

*Tubes*: Organic tube of nearly constant diameter, embedded in coral (see remarks).

*Sex and gametes*. Unknown.

**Remarks.** As explained above, Grube did not specifically mark his specimens as types of any kind. Grube (1878: 258) explicitly states that he had only one specimen. Wiktor (*loc. cit*.) marked the specimen known to her as holotype. However, 13 years later Hartwich (1993: 142) found a second specimen in the private collection of Grube, bought in 1881 by the Zoological Museum Berlin, and consequently labelled by Hartwich as ? syntype. A full evaluation of the material, syntype, holotype, or whatever, only can be given on the basis of the real material, in the context of a full taxonomic revision, not the intention of the present paper.

The original description by Grube (1878) emphasizes the remarkable long radiolar lobes, the low collar and the presence of radiolar ocelli in *S. tenuitorques*, as compared with two other species of *Potamilla*. Hartman (1959) placed *S*. (*Potamilla*) *tenuitorques* in *Potamilla*. However, Grube illustrated (Pl. 14: fig. 2) the typical crown, collar and chaetae of what nowadays is regarded as present in *Notaulax*. Our study corroborates the transfer of *Sabella* (*Potamilla*) *tenuitorques* to *Notaulax*, by the presence of long radiolar lobes, with dorsal and ventral flanges; and collar chaetae fascicles as single, elongate, oblique row of spine-like chaetae. Our description permits to distinguish *N. tenuitorques* from Australian, Philippine and Japanese congeners (Table 5). Among these, only *N. pyrrhogaster* and *N*. sp. 3 Capa & Murray (2015a) have radiolar ocelli distributed in groups, but in the first species the distal ocelli are distributed in a single row. In *N*. *pyrrhogaster, N. longithoracalis* and *N*. sp. 3 the collar fascicle is straight, oblique. However, the second species has only 4–6 radiolar ocelli in a single row, *N. pyrrhogaster* have 28–30 ocelli in groups basally and then distributed in a single row distally, whereas *N*. sp. 3 have 20 ocelli in groups at the middle of radiole only (Table 5). *Notaulax tenuitorques* and *N. pyrrhogaster* were found within the same locality. A detailed comparison between the latter species is given in the remarks for *N. pyrrhogaster*.

*Notaulax tenuitorques* was reported as *Potamilla* by Ehlers (1920) from Amboina (from the context of his introduction this can be narrowed down to present day Ambon). The tube microstructure of *N. tenuitorques* was described by Vinn, Zatoń & Tovar-Hernández (2018), and consists of an irregular mesh of thin, long and curved fibers with a chaotic orientation.

***Notaulax* sp. 3**

*Notaulax* sp. 3.— Capa & Murray, 2015a: 139–140, fig. 17.

**Material examined.** Indonesian-Dutch Snellius II Expedition, Indonesia, Sta. 4.114, N of Sumbawa, Bay of Sanggar, 08°19.2′S 118°14.4′E, lagoon side of reef barrier, 20 m depth, September 21–22, 1984, 1 anterior fragment [RMNH.VER. 19942].

**Description.** Trunk 10.5 mm long (lacking posterior abdomen), wide 1.1 mm. Radiolar crown 6.1 mm long with 10 pairs of radioles, with six pairs of ocelli in distal radiolar half. Elongate base of radiolar crown, as long as first three thoracic segments in lateral view: dorsal flanges prominent, triangular in side view; ventral flanges reduced. Palmate membrane as long as the length of the elongate basal radiolar lobes (compare Capa & Murray, 2015a: fig. 16A). Outer margins of radioles flat, with narrow flanges. Radiolar tips filiform with narrow flanges. Radiolar ocelli located at 1/2 of the radiole length, arranged in teardrop-shaped groups of 18–20 ocelli, occupying the length of 5–6 pinnular bases. Anterior peristomial ring lobe long, projecting anteriorly to collar margins, exposing mouth opening. Posterior peristomial ring collar incised: ventral margin long, triangular, with a short mid-ventral incision forming two discrete lappets; dorsal margin notched, slightly convex, not fused to the faecal groove. Collar chaetae arranged in straight oblique rows. Ventral shield of collar narrow, rectangular, other thoracic shields broad, quadrangular, laterally indented by neuropodial tori. Thorax with eight segments. Abdomen with more than 63 segments (posterior end missing).

**Remarks.** This anterior fragment fits the description by Capa & Murray (2015a) for *Notaulax* sp. 3.

***Notaulax montiporicola* sp. nov. Tovar-Hernández & ten Hove**

(Figs. 13–16)

**Figure 13:**
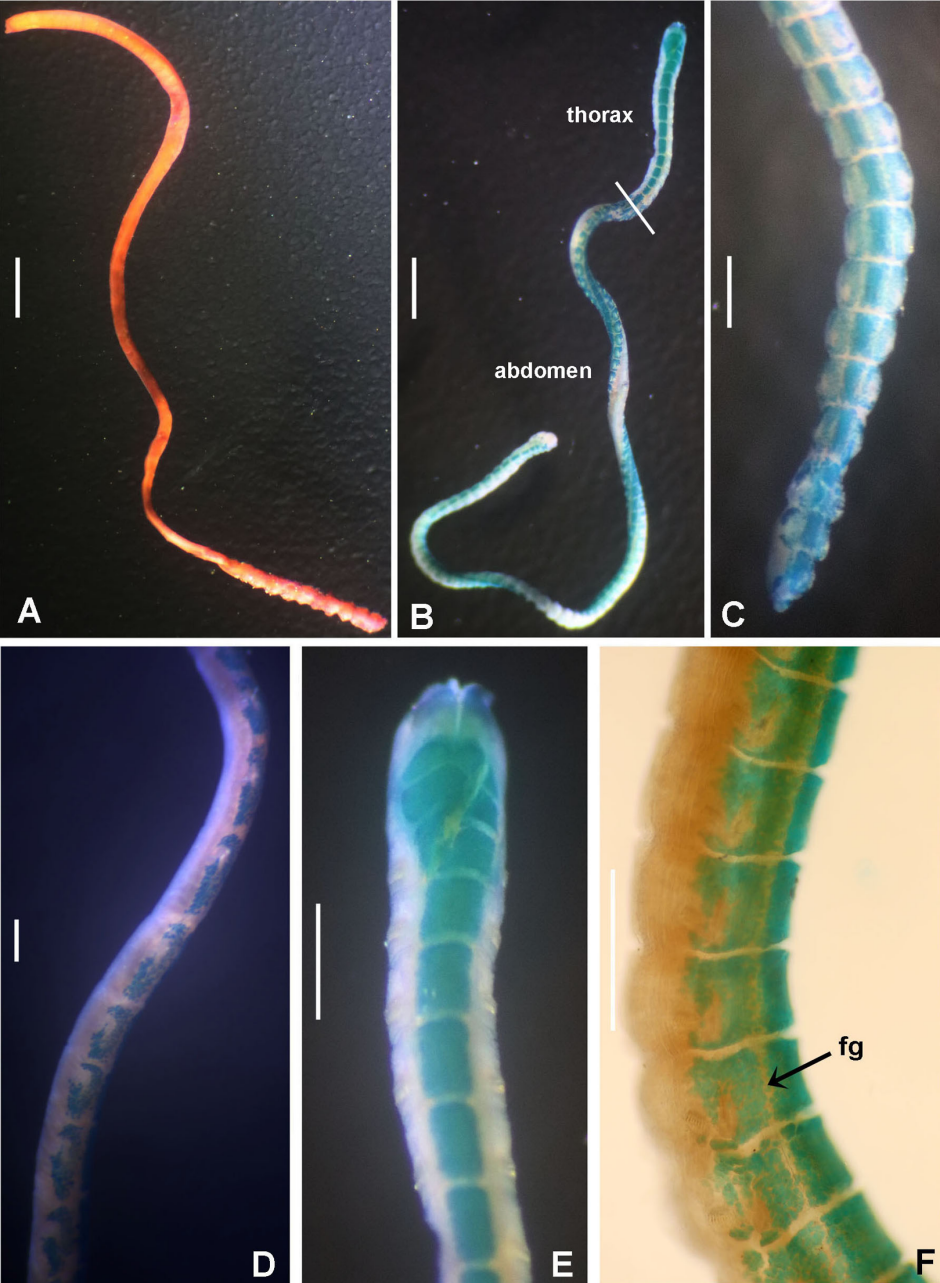
*Notaulax montiporicola* sp. nov. (A and B) Bodies, crown detached, (C) posterior end and pygidium, (D) anterior abdominal segments, right lateral view, (E) collar and thorax, ventral view, (F) posterior abdominal segments, right lateral view. Scale bars: (A and B) 1.2 mm, (C–F) 0.4 mm. Abbreviation: fg, faecal groove. Stain: (A) shirla, (B) methyl green. Specimens: (A) paratype RMNH.VER.19944, (B–F) holotype RMNH.VER.19943.

**Figure 14:**
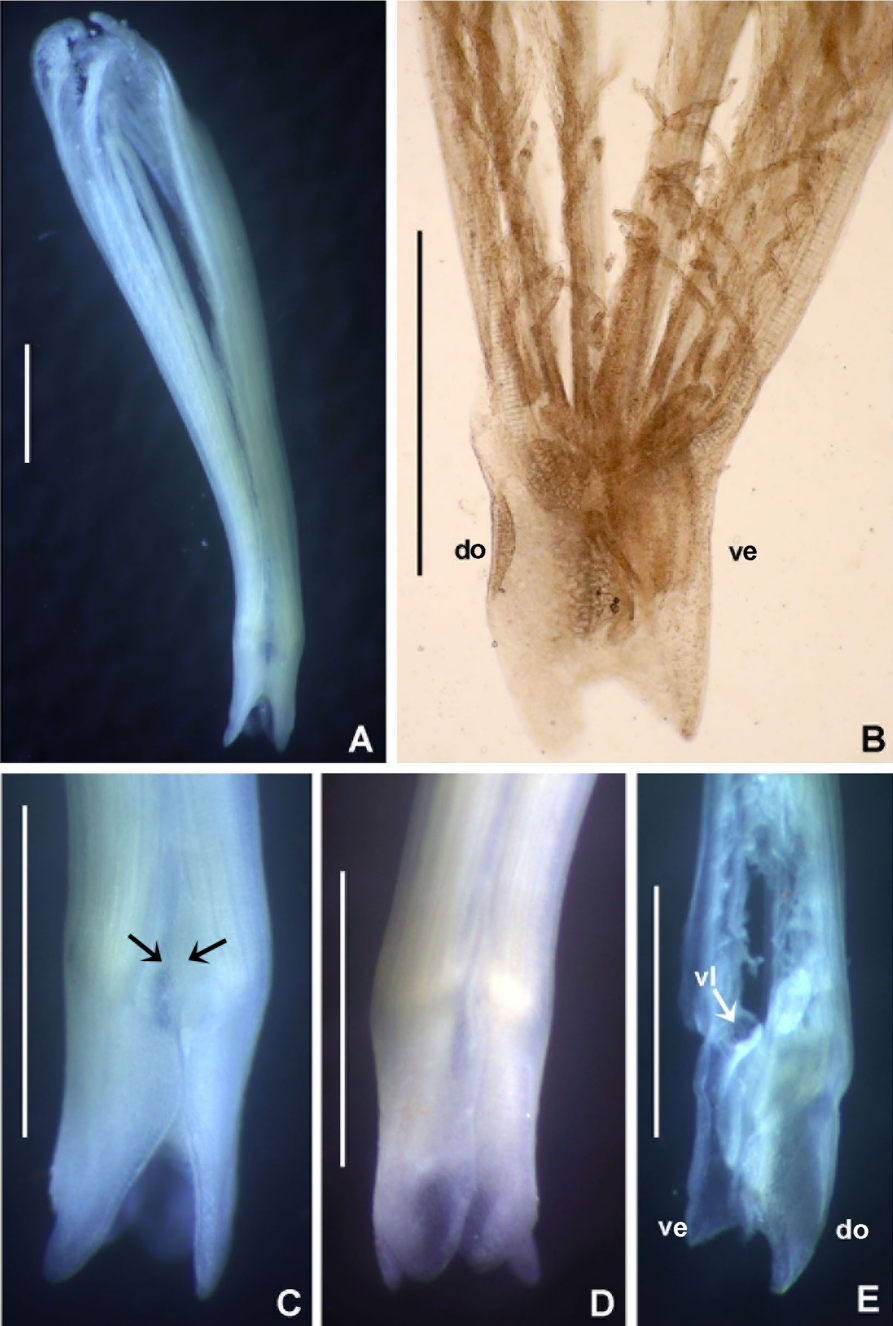
*Notaulax montiporicola* sp. nov. (A) Radiolar crown, dorsal view, (B) left radiolar lobe, (C) radiolar lobes, showing dorsal flanges and membrane indicated by arrows, (D) radiolar lobes, showing ventral, overlapping flanges, (E) right radiolar lobe. Scale bars: (A–E) 0.5 mm. Abbreviations: do, dorsal; ve, ventral; vl, ventral lip. (A, C–D) holotype RMNH.VER.19943; (B and E) paratype RMNH.VER.19944.

**Figure 15:**
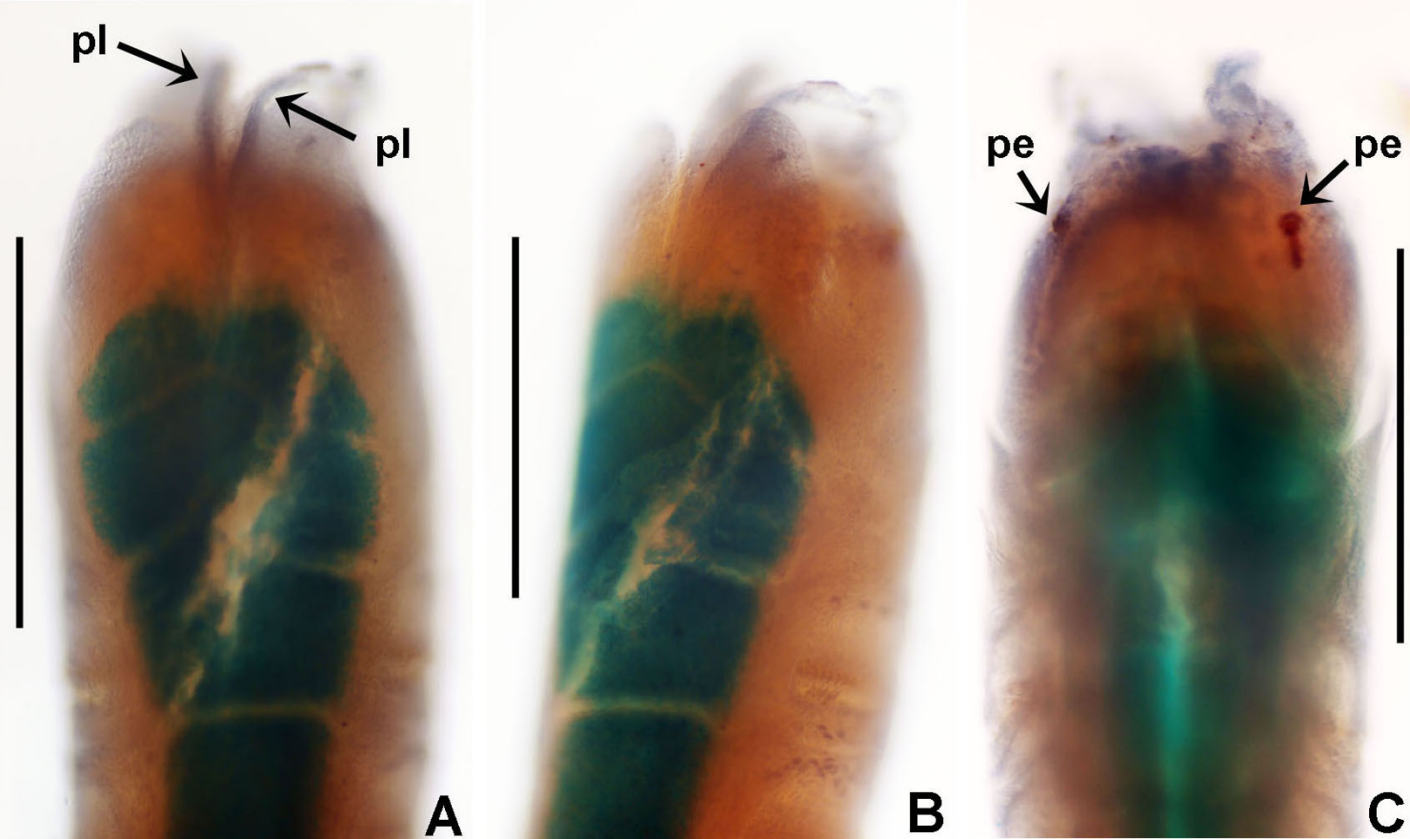
*Notaulax montiporicola* sp. nov. (A and B) Collar, ventral and ventro-left lateral views, (C) anterior peristomial ring and collar, dorsal view. (A–C) Holotype. Scale bars: (A–C) 0.4 mm. Abbreviations: pe, peristomial eyes; pl, parallel lamellae. Stain: (A–C) methyl green. (A–C) holotype RMNH.VER.19943.

**Figure 16:**
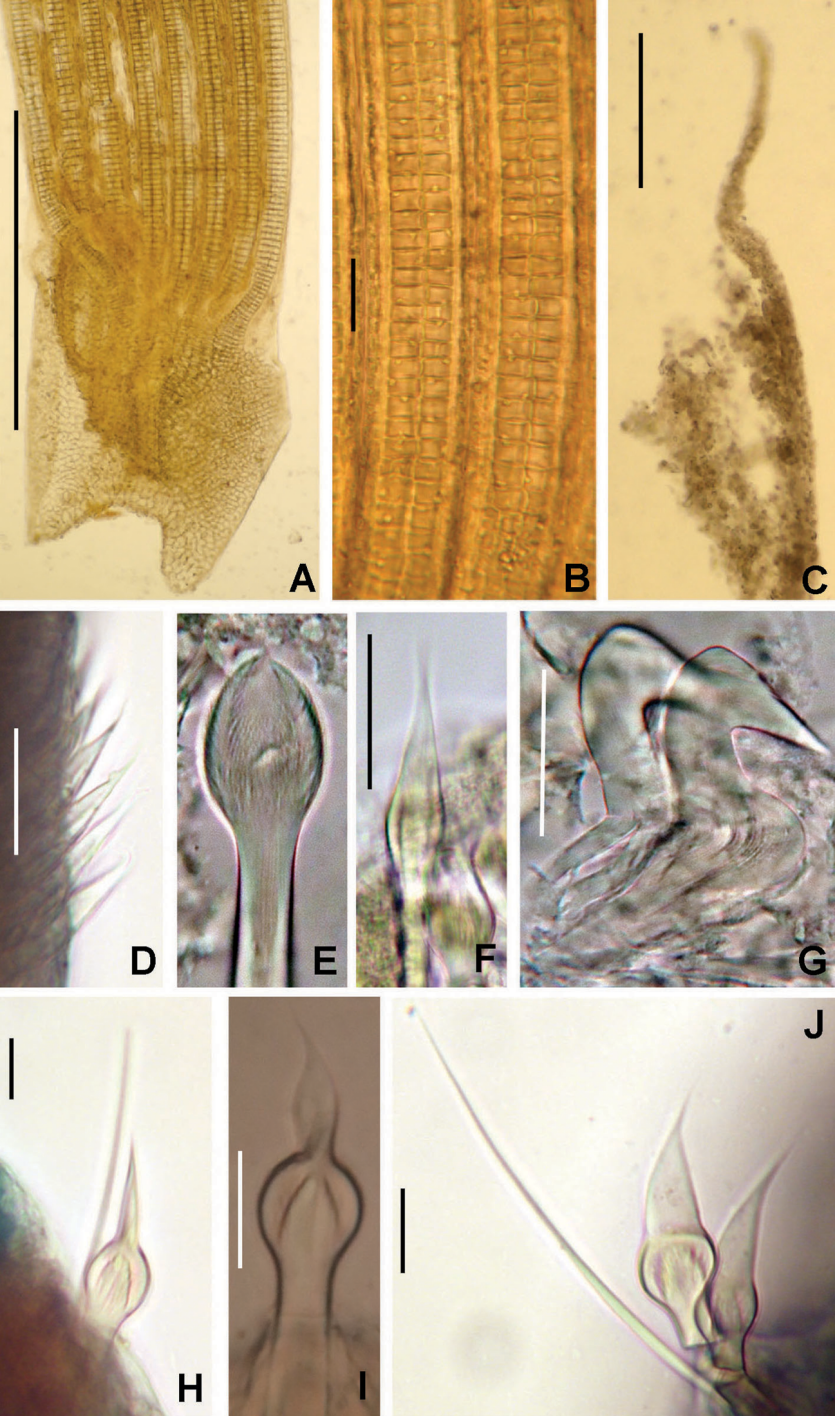
*Notaulax montiporicola* sp. nov. (A) Radiolar lobe, right lateral view, (B) radiolar cells, (C) radiolar tip, (D) spine-like chaetae from collar, (E) inferior thoracic paleate notochaeta, with reduced mucro, (F) superior thoracic spine-like notochaeta, (G) thoracic uncini, (H) superior abdominal paleate chaeta with long mucro and inferior narrowly hooded chaeta, (I) paleate, candle flame shaped chaeta from mid-abdomen, (J) paleate, sail-shaped chaetae from posterior abdomen and inferior narrowly-hooded chaeta. Scale bars: (A) 0.5 mm, (B) 10 μm, (C) 0.2 mm, (D) 50 μm, (E, G–J) 10 μm, F 30 μm. Specimens: (A–C) paratype RMNH.VER.19944, (D–K) holotype RMNH.VER.19943.

LSID: urn:lsid:zoobank.org:act:E2790CCF-B27C-44BF-BB96-A0F4D378317B

**Material examined.** Holotype [RMNH.VER. 19943] and 2 paratypes [RMNH.VER. 19944]: Indonesian-Dutch Snellius II Expedition, Sta. 4.030A, Tukang Besi Islands, Banda Sea, W coast of Binongko, 5°55′S, 123°59′E, coastal reef, in living coral *Montipora nodosa* (Dana, 1846), 3–6 m depth, September 10, 1984.

**Description.** *Colour, body shape, and size*. Body cream coloured, slim, thread-like (Figs. 13A and 13B). Thoracic, anterior and medium abdominal segments rectangular (Figs. 13A, 13B, and 13D–13F), posterior segments nearly rounded (Figs. 13A–13C). Trunk 18 mm long (17 mm), 0.4 mm wide (0.3 mm).

*Radiolar crown*. Length 4.7 mm (3.9 mm). Nine pairs of radioles (8–9). Radiolar lobes short (Fig. 14A), as long as two first chaetigerous segments, with dorsal and ventral basal flanges. Dorsal flanges well developed, triangular, not overlapping, with an anterior, short, triangular translucent membrane anterior to dorsal flanges, fused to dorsalmost radioles only for a short area (Figs. 14A–14C and 14E). Ventral flanges poorly developed (Fig. 14B), slightly overlapping (Fig. 14D). Base of dorsal flanges longer than base of ventral flanges (Figs. 14B and 14E). Palmate membrane, radiolar flanges and radiolar ocelli absent. Radioles with two rows of skeletal cells in side view; longest pinnules at mid-length. Radiolar tips long, filiform (Fig. 14A). Dorsal lips long, triangular, with a blood vessel visible in lip only. Ventral lips short, rounded lobes (Fig. 14E). Dorsal and ventral pinnular appendages absent.

*Peristomium*. Anterior peristomial ring exposed partially beyond collar. Parallel lamellae present (Fig. 15A). Dorsal margins of collar convex, entire (Figs. 15A and 15B). Two pairs of red, reniform, peristomial eyes (Fig. 15C). Posterior peristomial ring collar as long as 1.5 thoracic segments. Ventral margin of collar with a long mid-ventral incision, reaching anterior margin of collar ventral shield, forming two rounded lappets (Figs. 15A and 15B). Lateral collar margins convex, U-shaped, exposing anterior peristomial ring (Figs. 15A–15C).

*Thorax*. Chaetiger 1: chaetal fascicles with a straight, longitudinal row of short, spine-like chaetae (Fig. 16D); ventral shield divided in two transverse areas: basal area almost pentagonal, distal one dome-shaped (Fig. 15D). Chaetigers 2–12 (13): superior notochaetae spine-like (Fig. 16F). Inferior notochaetae paleate with reduced or vestigial mucros (Fig. 15E). Uncini avicular with main fang surmounted by several rows of small teeth, equal in size, covering half of main fang length; breast well developed; handles twice longer than main fang (Fig. 16G). Companion chaetae with distal ends teardrop-shaped membranes. Glandular ridge on chaetiger 2 absent. Ventral shields from chaetigers 2–13 rectangular (Fig. 13E). Tori separated from margins of ventral shields (Fig. 15E).

*Abdomen*. Segments: 68 (40). Rectangular glandular shields (Fig. 13F). Anterior abdominal fascicles with superior group of paleate chaetae with long, acicular mucros (longer than paleal length) (Fig. 16H) and 1–2 inferior elongate, narrowly hooded chaetae. Neuropodial uncini similar to thoracic ones, teeth covering more than 3/4 of main fang, 4–5 uncini per torus, short handles. Middle abdomen with transitional paleae: broad mucros candle flame-shaped, and posterior row elongate, narrowly hooded chaetae (Fig. 16I). Posterior abdomen with modified paleae: mucros expanded, sail-shaped, and posterior row elongate, narrowly hooded chaetae (Fig. 16J). Pygidium triangular, without anal cirrus or pygidial eyespots (Fig. 13C).

*Tubes*: Not preserved.

*Sex and gametes*. Unknown.

*Methyl green staining pattern*. Ventral thoracic shields stain uniformly (Figs. 13B–13F). Abdominal shields divided into two areas by faecal grove, which does not take stain (Fig. 13F). Entire dorsum and lateral sides of body pale, unstained (Figs. 13D–13F).

**Remarks.** This peculiar new species found in the living coral *Montipora nodosa*, at 30 m depth on the Western coast of Binongko Island, in the Banda Sea, is the first record of a sabellid polychaete inhabiting a living *Montipora* de Blainville, 1830. Nishi et al. (2017) provide a good synthesis of sabellids inhabiting corals. The specimens here reviewed were without the coral from which they were extracted, but labeled as “found in living coral”. The relation between corals and sabellids remains unknown, but this kind of interaction corresponds to bioclaustration. Species of *Anamobaea*, *Hypsicomus*, *Notaulax*, *Perkinsiana* and *Pseudopotamilla* have been reported in coral masses either, but if these were alive or dead, or if there was a real symbiosis is unclear (Perkins, 1984; Tovar-Hernández & Salazar Vallejo, 2006; Capa, 2007; Nishi et al., 2017). A case of a sabellid modifying a coral surface is reported below for *Perkinsiana anodina* (see below).

*Notaulax montiporicola* sp. nov., is similar to most species in the genus in that the base of the radiolar crown is long, equipped with dorsal and ventral flanges, and chaetal fascicles have a straight, longitudinal row of spine-like chaetae. However, *N. montiporicola* sp. nov., does not have radiolar ocelli (present in *Anamobaea* Krøyer, 1856 and *Notaulax*); it is unknown if such ocelli may fade off after years of preservation, although the peristomial eyes (not reported in *Notaulax* neither in *Anamobaea*) have not faded over the years. In addition, *Notaulax montiporicola* sp. nov., is unique by the presence of two remarkable types of mucros in the abdominal paleate chaetae: with candle flame-shaped mucros in anterior abdominal segments, sail-shaped mucros posteriorly. Unfortunately, only three specimens were collected and the use of scanning electron microscopy for a better documentation of these changing chaetal forms was not possible. In addition, the new taxon show characters that do not match entirely with *Anamobaea* or *Notaulax* such as the absence of radiolar eyes, palmate membrane, radiolar flanges, and ventral sacs. A full phylogenetic analysis, not possible in the context of the present paper, is needed to decide whether or not these characters merit a genetic distinction.

**Etymology.** The specific name refers to the fact that the species was found in a living coral (*Montipora*, combined with the Latin -cola = ‘dweller’). It should be regarded as invariant compound noun in apposition (compare Read et al., 2017: 19).

**Genus *Parasabella*Bush, 1905** (pp 191, 199–200)

*Demonax* Kinberg, 1867: 354 (not Thomson, 1860).— Kinberg, 1910: 72; Johansson, 1925: 26–27; Johansson, 1927: 136; Knight-Jones, 1983: 254; Perkins, 1984: 292–293; Knight-Jones & Walker, 1985: 605; Fitzhugh, 1989: 75–76; Giangrande, 1994: 229–230.

*Parasabella*.— *fide* Johansson, 1927: 136; Tovar-Hernández & Harris, 2010:14; Capa & Murray, 2015b: 773, 775–776; Tovar-Hernández, de León-González & Bybee, 2017: 27–28; Capa et al., 2019: 199.

*Distylidia* Hartman, 1961: 129.— *fide* Fauchald, 1977b: 138; Banse, 1979: 870.

**Type species**: *Parasabella media* Bush, 1905, by original designation.

**Number of species**: 27, after Tovar-Hernández, de León-González & Bybee (2017).

**Remarks.** Diagnoses to genus level are available in Perkins (1984), Fitzhugh (1989) and Capa & Murray (2015b). Species of *Parasabella* from Australia were studied by Capa & Murray (2015b) based on the comparison of morphological data, nuclear and mitochondrial DNA sequence data. They found seven distinct genetic lineages of *Parasabella* in Australia: *P. aberrans* (Augener, 1926), *P*. sp. cf. *P. aulaconota* (von Marenzeller, 1884 (1885)), *P. bioculata* Capa & Murray, 2015b, *P. crassichaetae* complex Capa & Murray, 2015b, *P*. sp. cf. *P. japonica* (Moore & Bush, 1904), *P*. sp. cf. *P. rugosa* (Moore, 1905) and another unnamed species (*P*. sp. 1).

Notwithstanding the fact that Capa & Murray (2015b) examined many specimens, including some types, they were unable to attribute all to a previously described or an evident new species. The taxonomic study of *Parasabella* species is not simple because many of the original descriptions are brief, incomplete, or not illustrated; further, records are doubtful because unique morphological diagnostic features are lacking. Moreover, there are cases of translocation of species out of their natural distribution range (Capa & Murray, 2015b).

A full revision of the genus, based on redescriptions of types, designation of neotypes, and supported by genetic data is needed. In the Indo Pacific Region, the identity of lineages as *P*. cf. *aulaconota*, *P. japonica* or *P*. cf. *rugosa* should be elucidated. In Japan, three species of *Parasabella* were described: *P. japonica*, *P. fullo* (Grube, 1878) and *P. albicans*. Parasabella fullo was redescribed by Keppel, Ruiz & Tovar-Hernández (2020), whereas the the status of the other two species demands further study. Two more nominal species should be taken into account: *P. rufovittata* (Grube, 1881), described as *Sabella* from Singapore (type material in the Zoological Museum of Berlin, No. 870) and *P. oculea* (Pillai, 1965) from the Philippines (type material in the University of Sri Lanka, RTS 25) since these taxa might have a larger distribution in the region.

***Parasabella crassichaetae* complex Capa & Murray, 2015b**

*Parasabella crassichaetae* complex Capa & Murray, 2015b: 787, 789–791, figs 4E, 5E, R–T, 12, 13.

**Material examined.** Indonesian-Dutch Snellius II Expedition, Sta. 4068, Indonesia, NE coast of Sumba, 09°57′S, 120°48′E, Agassiz trawl, 50 m depth, sandy bottom with sponges and gorgonians, on a whitish ascidian mat, legit R.W.M. van Soest, September 16, 1984, 13 specimens [RMNH.VER. 19945].

**Description.** Trunk 4.5 mm long, 0.7–0.8 mm wide. Radiolar crown 2.5 times longer than thorax, with seven pairs of radioles. Radiolar eyes absent. Thorax with 6–10 chaetigers. Thoracic ventral shields contact neuropodial tori. Inferior thoracic notochaetae Type A, with hoods three times wider than shaft, and up to three times as long as its maximum width. Thoracic uncini with medium length handles, and neck half of breast length. Abdomen with 15 chaetigers.

**Remarks.** *Parasabella crassichaetae* was originally described from Shellharbour (New South Wales, Australia) on an orange sponge from 22.4 m depth (Capa & Murray, 2015b). Its apparent translocation to Queensland, Western Australia and Hawaii, where the species was found in dead coral rubble, sponges, algae and artificial surfaces in ports and harbours, is remarkable. Our specimens have inferior thoracic chaeta type A: broadly hooded with short, abruptly tapering tips, and as such match this complex.

Tubes from the NE coast of Sumba were found below the surface of ascidian mat, with anterior tubes openings directed toward the surface. One specimen was found with three copepods attached to its radiolar tips.

Capa & Murray (2015b) reported regeneration of the posterior end in the holotype and presence of eggs in additional specimens. Specimens reviewed in our study include many buds produced by asexual reproduction. These buds were found within tubes of parents, with transverse fission and regeneration. Some buds have vestigial radiolar crowns (0.2 mm long), with developing radioles without pinnules, or presenting only an anterior blastema. Other buds consist only of trunks formed by 18–23 abdominal segments (no thoracic segments). Transverse fission and regeneration were also reported in *Parasabella columbi* (Kinberg, 1867) from Argentina (Tovar-Hernández, de León-González & Bybee, 2017).

Detailed illustrations of *Parasabella crassichaetae* complex were given in the original description by Capa & Murray (2015b).

**Genus *Perkinsiana*Knight-Jones, 1983** (p. 273–274)

*Perkinsiana*.— Fitzhugh, 1989: 78; Capa, 2007: 549; Tovar-Hernández et al., 2012: 57; Capa et al., 2019: 199.

**Type species**: *Sabella rubra* Langerhans, 1880, by original designation.

**Number of species**: 18, after Tovar-Hernández et al. (2012).

**Remarks.** *Perkinsiana* was proposed by Knight-Jones (1983) to accommodate species previously included in *Demonax* Kinberg, 1867 (= *Parasabella fide* Tovar-Hernández & Harris, 2010), *Potamilla* Malmgren, 1866 and *Potamethus* Chamberlin, 1919. Fitzhugh (1989) modified the generic diagnosis by Knight-Jones (1983), and pointed out that *Perkinsiana* could not be defined by any synapomorphy. Rouse (1996) emended the generic diagnosis to incorporate features found in other species in his opinion belonging to the taxon. Capa (2007) emended the genus again in order to include changes needed after the exclusion of *P. riwo* Rouse, 1996 (included in *Kirkia* Nogueira, López & Rossi, 2004 and transferred to *Aracia* Nogueira, Fitzhugh & Rossi, 2010, when *Kirkia* was recognized as a homonym) and the addition of two new species (*Pekinsiana* (sic) *longa* Capa, 2007, and *P. anodina* Capa, 2007). Tovar-Hernández et al. (2012) provided a new emendation to *Perkinsiana* and defined three types (A, B, C) of abdominal chaetae in the genus. Type A for chaetae with a broad hood and progressively tapered. Type B for chaetae having a basal broad knee and tips abruptly narrowed. Type C for elongate abdominal chaetae with narrow hoods.

***Perkinsiana anodina*Capa, 2007**

(Fig. 17)

**Figure 17:**
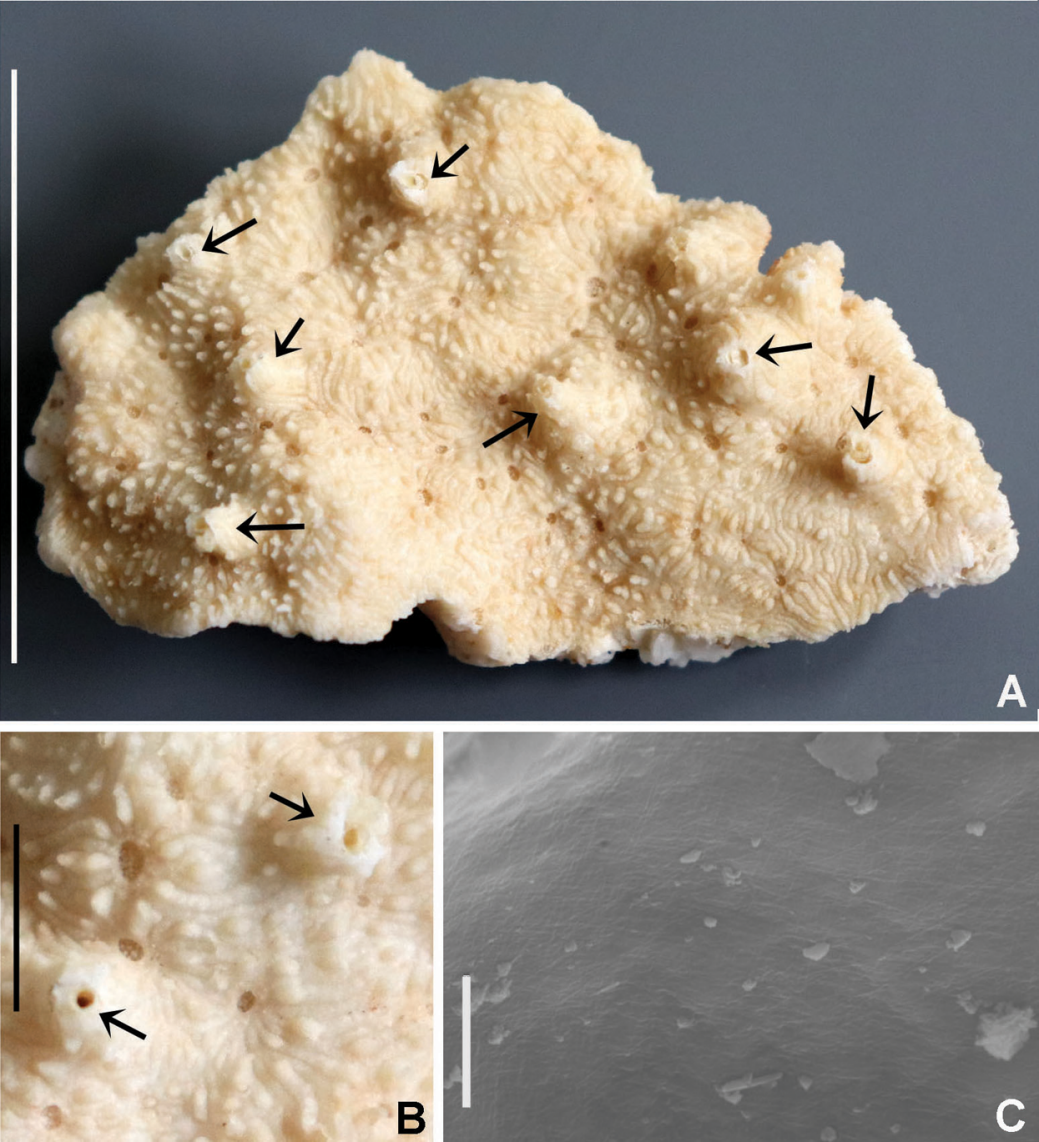
Tubes and microstructure of *Perkinsiana anodina*. (A and B) Coral *Cycloseris wellsi* showing tubes of *P. anodina*, (C) tube microstructure: surface of lumen showing fibers of three different orientations. Scale bars: (A) 3.5 cm, (B) 0.6 cm, (C) 5 μm. Specimens: (A and B), RMNH.VER. 19946.

*Perkinsiana anodina* Capa, 2007: 549, 551, figs 4J, K, 6, 7A–G; Capa & Murray, 2015a: 147–148, fig. 20G–J.

**Material examined.** Indonesian-Dutch Snellius II Expedition, Sta. 4133. NE Takabonerate (Tiger Island), E coast of Tarupa Kecil, 06°29′S 121°8′E, littoral zone, sandy beach, beach rock, seagrass, in living coral, 8–11 m depth, 26 September, 1984, 5 specimens [RMNH.VER. 19946]. Sta. 4114, Indonesia, N of Sumbawa, Bay of Sanggar, 08°19.2′S 118°14.4′E, 20 m depth, lagoon side of reef barrier, 21–22 September 1984, 2 specimens and 4 embedded in coral mass [RMNH.VER. 19947].

**Description.** Radiolar flanges and palmate membrane absent. Radioles with rectangular outer margin and digitiform tips. Anterior peristomial ring large, as long as two thoracic segments, exposed dorso-laterally. Posterior peristomial ring collar with dorsal margins separated by a wide gap. Ventral shield of collar quadrangular, broader than the following shields, with straight anterior margin. Ventral shield of chaetiger 2 rectangular with triangular anterior margin. Other shields rectangular with straight anterior margins and of similar length along thorax, separated from uncinial tori by wide gap. Abdominal chaetigers with chaetae Type B. Tubes embedded in live coral, anterior parts emerging as small cones above coral surface (Figs. 17A and 17B).

**Tube microstructure.** Lumen relatively smooth, showing faint micro-relief caused by sparsely spaced fibers. Three different sets of fibers oriented at about 45° or 90° to each other. Fibers in single sets are moderately-developed, long (>13 μm), usually straight and parallel to each other. Interspaces of fibers larger than fiber diameter. Interspaces of adjacent fibers filled with homogeneous organics with smooth surface. Fibers with constant width (0.10 to 0.20 μm). Tube wall dense, non-porous (Fig. 17C).

**Remarks.** *Perkinsiana anodina* was described from Western Australia with specimens living in dead coral, large granite boulders with small colonies of live and dead corals, on sand with scattered bommies, and on mussel clumps from pilings of a jetty. It was also reported from the Northern Territory (Capa, 2007) and on coral ruble in Queensland (Capa & Murray, 2015a).

In our study, *P. anodina* was found surrounded by tissue of the mushroom coral *Cycloseris wellsi* (Veron & Pichon, 1980). Tubes of *P. anodina* form straight protuberances on the scleractinian coral surface (Figs. 17A and 17B). These modifications on the living coral suggest a case of symbiosis, as occurs with some spionid (Liu & Hsieh, 2000; Wielgus, Glassom & Chadwick-Furman, 2002; Wielgus, Glassom & Chadwick-Furman, 2006) and chaetoperid polychaetes (Bergsma, 2009), that have been found associated with species of *Montipora*. As is the case for *P. anodina*, spionids and chaetopterids induce formation of finger-like branchlets in the host, and other skeletal aberrations (Molodtsova, Britayev & Martin, 2016) but ecological studies are needed to clarify which type of association is involved. Among Sabellinae three associations have been reported: *Potamilla symbiotica* Uschakov, 1950, is an obligate commensal associated with the hydroid *Cryptospongia enigmatica* Burton, 1928 (Martin & Britayev, 1998); however, according to WoRMS, this association deserves clarification because *C. enigmatica* is a *taxon inquirendum* (Schuchert, 2020); *Terebrasabella heterouncinata* Fitzhugh & Rouse, 1999, is a parasite of cultured abalones (Martin & Britayev, 2018, and references therein); finally, *Amphicorina schlenzae* Nogueira & Amaral, 2000 came from dead parts of the coral *Mussismilia hispida* (Verrill, 1902), which also had algae and sponge, consequently cannot be symbiotic (Nogueira, 2020, personal communication). In the Serpulidae, sister group of Sabellidae, many cases of commensalism with corals have been documented including species of *Spirobranchus* de Blainville, 1818, *Circeis* de Saint-Joseph, 1894, *Spirorbis* Daudin, 1800, and *Vermiliopsis* de Saint-Joseph, 1894, and fossils of *Propomatoceros* Ware, 1975, and *Josephella* Caullery & Mesnil, 1896 (Martin & Britayev, 1998; Martin & Britayev, 2018 and references therein). Hoeksema et al. (2019); however, the question is whether the association worm/coral is commensalistic or rather amensalistic.

The tube wall microstructure of *P. anodina* is not in any way special as compared to the other studied species. This indicates that symbiosis with coral has had no effect on the tube microstructure of the species, possibly because *Perkinsiana anodina* has no interaction with the host coral through its tube wall.

Regarding morphology, the presence of a palmate membrane in *P. anodina* is doubtful. Capa (2007: fig. 6B) reported and illustrated the presence of a low membrane between two lateral radioles, but they resemble pinnular tissue instead of a proper membrane. In her figure 6C, dorsal radioles are not fused basally by a membrane. Our specimens do not have a palmate membrane joining radioles.

Type B abdominal chaetae in *Perkinsiana* were referred to as *with a bulbous knee* in Knight-Jones (1983) or *elongate, broadly-hooded* in Fitzhugh (1989) and Capa (2007). Type B abdominal chaetae are present in the type species, *P. rubra* (Langerhans, 1880), and in *P. socialis* (Langerhans, 1884), *P. fonticula* (Hoagland, 1919), *P. ceylonica* (Augener, 1926), *P. linguicollaris* (Day, 1961), *P. anodina* and *P. longa*. A comparison between *P. anodina* and *P. longa* was made by Capa (2007). *Perkinsiana socialis*, *P. fonticula* and *P. linguicollaris* have long, triangular ventral lappets, whereas in *P. anodina*, *P. ceylonica* and *P. rubra* ventral lappets are short.

**Genus *Pseudopotamilla*Bush, 1905** (pp 203–204)

*Pseudopotamilla.—* Knight-Jones, 1983: 253–254; Fitzhugh, 1989: 79–80; Capa, 2007: 555; Knight-Jones et al., 2017: 203; Tovar-Hernández, de León-González & Bybee, 2017: 47–48; Capa et al., 2019: 201.

**Type species**: *Amphitrite reniformis* Bruguière, 1789, by original designation.

**Number of species**: 19–23, under revision, see remarks below.

**Remarks.** Diagnoses to genus level are available in Knight-Jones (1983), Fitzhugh (1989), Capa (2007) and Knight-Jones et al. (2017). The number of validly described species in *Pseudopotamilla* is not clear at all since there is no worldwide revision of the genus. WoRMS lists 19 species (Read & Fauchald, 2020b) but 23 species are included in Table 6. Note that we regard the status of some nominal species to be questionable. Discrepancies in numbers between WoRMS and Table 6 are due to recombinations, synonymizations, homonyms, revalidations of some species, and transfer to other sabellid genera such as *Acromegalomma*.

**Table 6:** Species of *Pseudopotamilla* from the world.

Species name	Nomenclatural citation	Country, type locality	Comment
*Pseudopotamilla alba* (Knox, 1951)	*Potamilla alba* Knox, 1951: 76–79, figs 19–23	New Zealand, Banks Peninsula, 44°15′S, 173°31′E	Capa (2007: 559) briefly mentioned this species, she did not compare her new species from Australia with *P. alba*
*Pseudopotamilla aspersa* (Krøyer, 1856)	*Sabella aspersa* Krøyer, 1856: 19–20	Greenland	Reinstated by Knight-Jones et al. (2017)
*Pseudopotamilla cerasina* (Grube, 1871)	*Sabella* (*Potamilla*) *cerasina* Grube, 1871: 67	Croatia, Lussin Piccolo	In many papers the year of publication was 1870 but it is not correct. The volume (1871) per se, is an annual report of the society, which includes a meeting report from 1870
*Pseudopotamilla debilis* Bush, 1905	*Pseudopotamilla debilis* Bush, 1905: 204, pt. 36, figs 23–24, 26	USA, California, Pacific Grove	
*Pseudopotamilla elegans* (Johansson, 1922)	*Potamilla elegans* Johansson, 1922: 7–8, pt. 1, fig. 5	Japan	Questionable status in *Pseudopotamilla*. Revision of types is needed. It appears in *Pseudopotamilla* according to WoRMS (Read & Fauchald, 2020b), but, original description states that radiolar eyes are missing
*Pseudopotamilla fitzhughi* Tovar-Hernández & Salazar Vallejo, 2006	*Pseudopotamilla fitzhughi* Tovar-Hernández & Salazar Vallejo, 2006: 58–60, figs 19–21	Mexico, Caribbean, Contoy Island	
*Pseudopotamilla intermedia* Moore, 1905	*Pseudopotamilla intermedia* Moore, 1905: 562–564, pt. 37, figs 15–22	Alaska, off Cape Edgecumbe, Sitka Sound, 922 fathoms, on soft gray mud	
*Pseudopotamilla knightjonesae* Tovar-Hernández, de León-González & Bybee, 2017	*Pseudopotamilla knightjonesae* Tovar-Hernández, de León-González & Bybee, 2017: 50, 59, figs 28–30, 33C	Argentina, Santa Clara del Mar, 37°50′30″S, 57°29′58″W, intertidal	
*Pseudopotamilla laciniosa* (Ehlers, 1904)	*Potamilla laciniosa* Ehlers, 1904: 66–67, pt. 9, figs 7–10	New Zealand	It was synonymised with *P. oligophthalmos* by Augener (1914, 1926) and then followed by Johansson (1927). Capa (2007) mentioned that recent papers have been considered *P. laciniosa* as valid species, but sources to these papers were not mentioned
*Pseudopotamilla latisetosa* (Grube, 1840)	*Sabella latisetosa* Grube, 1840: 61–62, fig 11	Italy, Palermo	According to Knight-Jones et al. (2017), type ZMB 5166 lacks radiolar crown and type ZMB 5242 lacks radiolar eyes
*Pseudopotamilla monoculata* Capa, 2007	*Pseudopotamilla monoculata* Capa, 2007: 556, figs. 10, 11A–G, 12A–F	Australia, Tasman Sea, 15 Km E of Maria Island, 42°37′S, 148°20′E	
*Pseudopotamilla myriops* (von Marenzeller, 1884 (1885))	*Potamilla myriops* von Marenzeller, 1884 (1885): 211, pt. 3, fig. 2	Japan	It was synonymised with *P. oligophthalmos* by Augener (1914, 1926) and then followed by Imajima & Hartman (1964) and Banse & Hobson (1968)
*Pseudopotamilla occelata* Moore, 1905	*Pseudopotamilla occelata* Moore, 1905: 559–562, pt. 37, figs 8–14	Canada, Vancouver Island, off Fort Rupert, 14–19 fathoms, gray sand with rocks	
*Pseudopotamilla oculifera* (Leidy, 1855)	*Sabella oculifera* Leidy, 1855: 145, pt. 11, figs 55–61	USA, Rhode Island	According to Bush (1905), it is a synonym of *P. reniformis*. Knight-Jones et al. (2017) states that type was not found, but possibly belongs to *P. reniformis*
*Pseudopotamilla oligophthalmos* (Grube, 1878)	*Sabella* (*Potamilla*) *oligophthalmos* Grube, 1878: 248−249	Singapore	New combination (present study)
*Pseudopotamilla platensis* (Hartman, 1953)	*Potamilla platensis* Hartman, 1953: 53–54, fig. 19a–f	Argentina, north Argentina, 37°15′S, 56°8′W, 100 m depth	
*Pseudopotamilla polyophthalmos* (Grube, 1878)	*Sabella* (*Potamilla*) *polyophthalmos* Grube, 1878: 247–248, pl. 15, fig. 2	Philippines	New combination (present study). The specific name is remarkable: the presence of two homonyms with *Pseudopotamilla polyophthalma* Hartmann-Schröder, 1965, a species described from Punta Lavapie (central Chile), and *Sabella* (*Potamilla*) *reniformis* var. *polyophthalmos* Langerhans, 1884
*Pseudopotamilla polyophthalmos* (Langerhans, 1884)	*Sabella* (*Potamilla*) *reniformis* var. *polyophthalmos* Langerhans, 1884: 266–267	Madeira	Augener (1914: 255) emphasized the need of a review of the homonyms involved (*polyophthalmos* Grube and *polyophtalmos* Langerhans). Examination of type is needed, but Madeiran species might be *P. saxicava* (see Knight-Jones et al., 2017)
*Pseudopotamilla polyophthalma* Hartmann-Schröder, 1965	*Pseudopotamilla polyophthalma* Hartmann-Schröder, 1965: 271–273, figs 273–275	Chile, Punta Lavapié	The name is homonym of *P. polyophtalmos* Grube and *P. polyophtalmos* Langerhans. See Remarks on *Pseudopotamilla polyophthalma* Hartmann-Schröder, 1965 in the text
*Pseudopotamilla reniformis* (Bruguière, 1789)	*Amphitrite reniformis* Bruguière, 1789: 57–58	Iceland	Redescribed by Knight-Jones et al. (2017)
*Pseudopotamilla saxicava* (de Quatrefages, 1866)	*Sabella saxicava* de Quatrefages, 1866: 437–438	France, Guettary	Reinstated by Knight-Jones et al. (2017)
*Pseudopotamilla socialis* Hartman, 1944	*Pseudopotamilla socialis* Hartman, 1944: 282–283, pt. 24, figs 53–58	USA, California, Tomales Point, Marin County, ocean side, in sponge, among rocks, intertidal	
*Pseudopotamilla tortuosa* (Webster, 1879)	*Potamilla tortuosa* Webster, 1879: 65–66, pt. 10, figs 149–153	USA, Virginia	

Regarding *Pseudopotamilla* from the Indonesian archipelago, Philippine Seas, Australia and New Zealand, five nominal species have been described. Grube (1878) described *Sabella* (*Potamilla*) *oligophthalmos* from Singapore, and *Sabella* (*Potamilla*) *polyophthalmos* from the Philippines. In our study both species are transferred to *Pseudopotamilla* (Table 6). *Pseudopotamilla alba* (Knox, 1951) and *P. laciniosa* (Ehlers, 1904) were described from New Zealand and appear to be currently valid (Table 6). In addition, Capa (2007) described *P. monoculata* from the Tasman Sea and recorded two additional, unnamed species as *Pseudopotamilla* sp. A (from Sydney) and *Pseudopotamilla* sp. B (from New South Wales, Queensland and Western Australia). Later, Capa & Murray (2015a), placed these two taxa in the preliminary synonymy of *Pseudopotamilla* sp. cf. *P. reniformis* from Lizard Island.

***Pseudopotamilla*** ***oligophthalmos* (Grube, 1878), new combination**

(Figs. 18–20)

**Figure 18:**
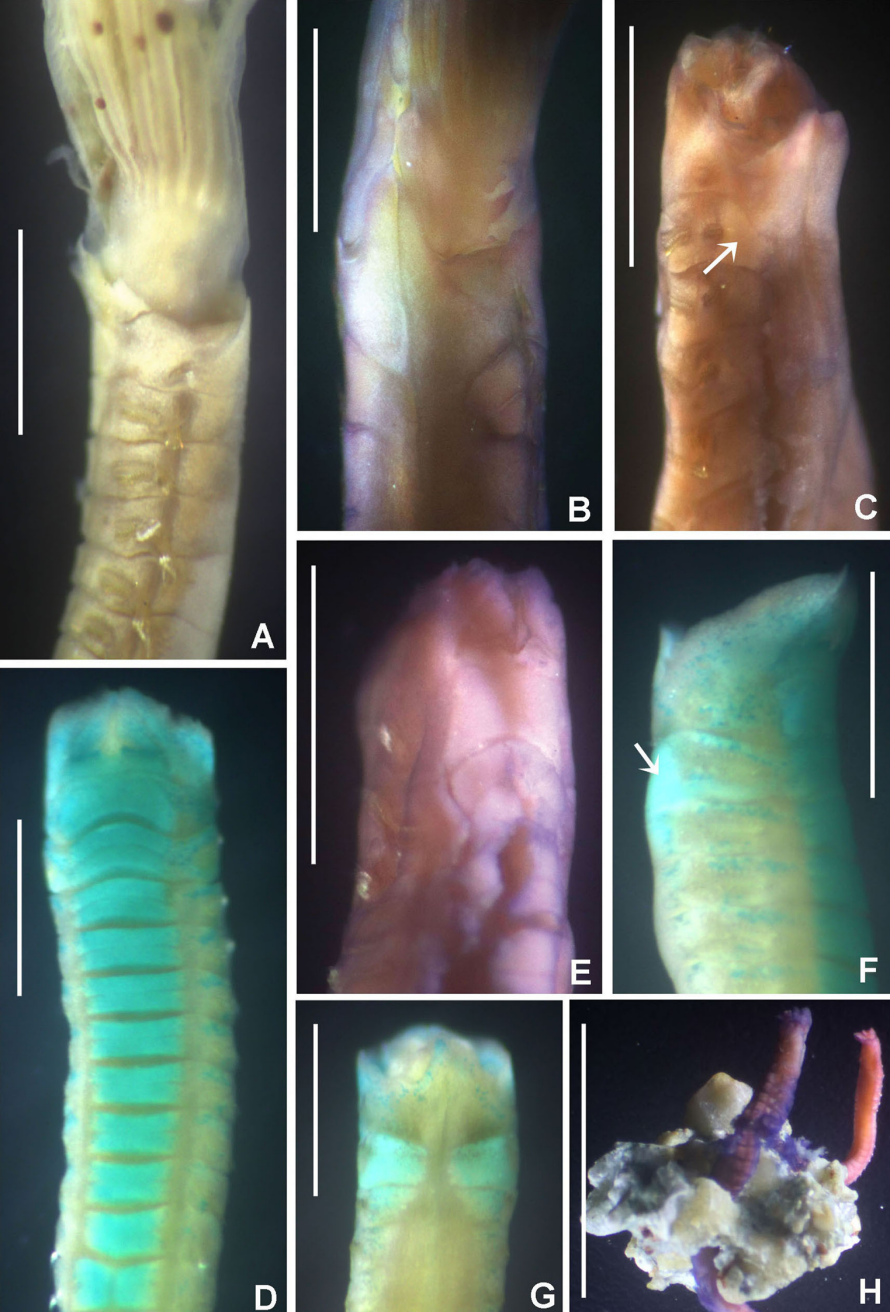
*Pseudopotamilla oligophthalmos*. (A) Base of crown and anterior thoracic segments, left lateral view, (B) collar, dorsal view, (C) collar and anterior thoracic segments, dorsolateral view, (D) thorax, ventral view, (E) collar and anterior thoracic segments, ventral view, (F) same, lateral view, (G) same, dorsal view, (H) calcareous mass with projecting sabellid bodies. Arrows in (C) and (F) indicate dorsal glandular shields. Scale bars: (A and B) 0.8 mm, (C and E) 1 mm, (D, F–G) 0.5 mm, (H) 6 mm. Stain: (B–C, E, H) shirla, (D, F–G) methyl green. Specimens: (A–H) RMNH.VER. 19948.

**Figure 19:**
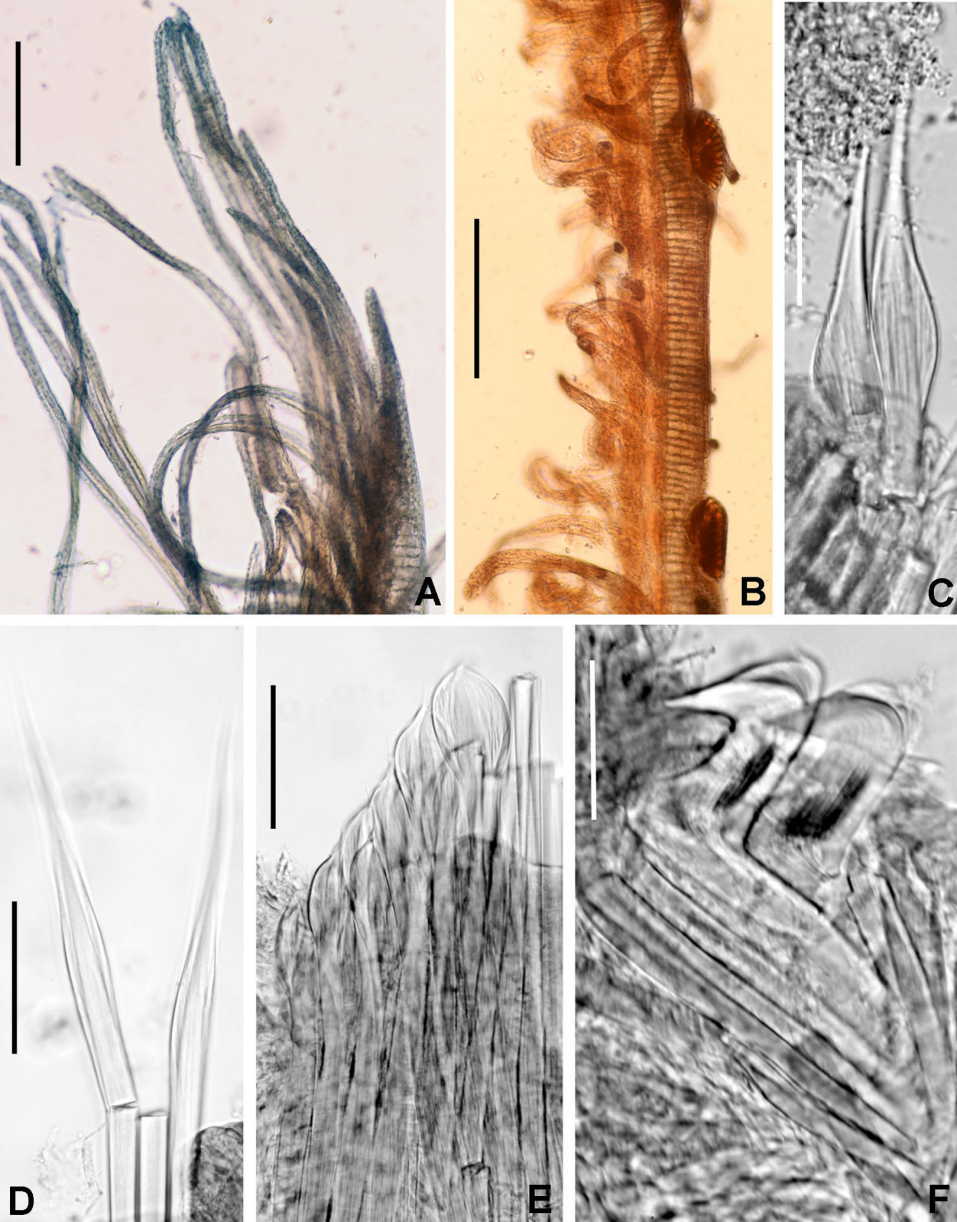
*Pseudopotamilla oligophthalmos*. (A) Radiolar tip and distalmost pinnules, (B) radiolar eyes, (C) broadly hooded chaetae from collar, (D) superior thoracic notochaetae elongate narrowly-hooded, (E) inferior thoracic notochaeta paleate, (F) thoracic uncini and manubria of companion chaetae. Scale bars: (A and B) 0.2 mm, (C–E) 60 μm, (F) 20 μm. Single specimen, RMNH.VER. 19948.

**Figure 20:**
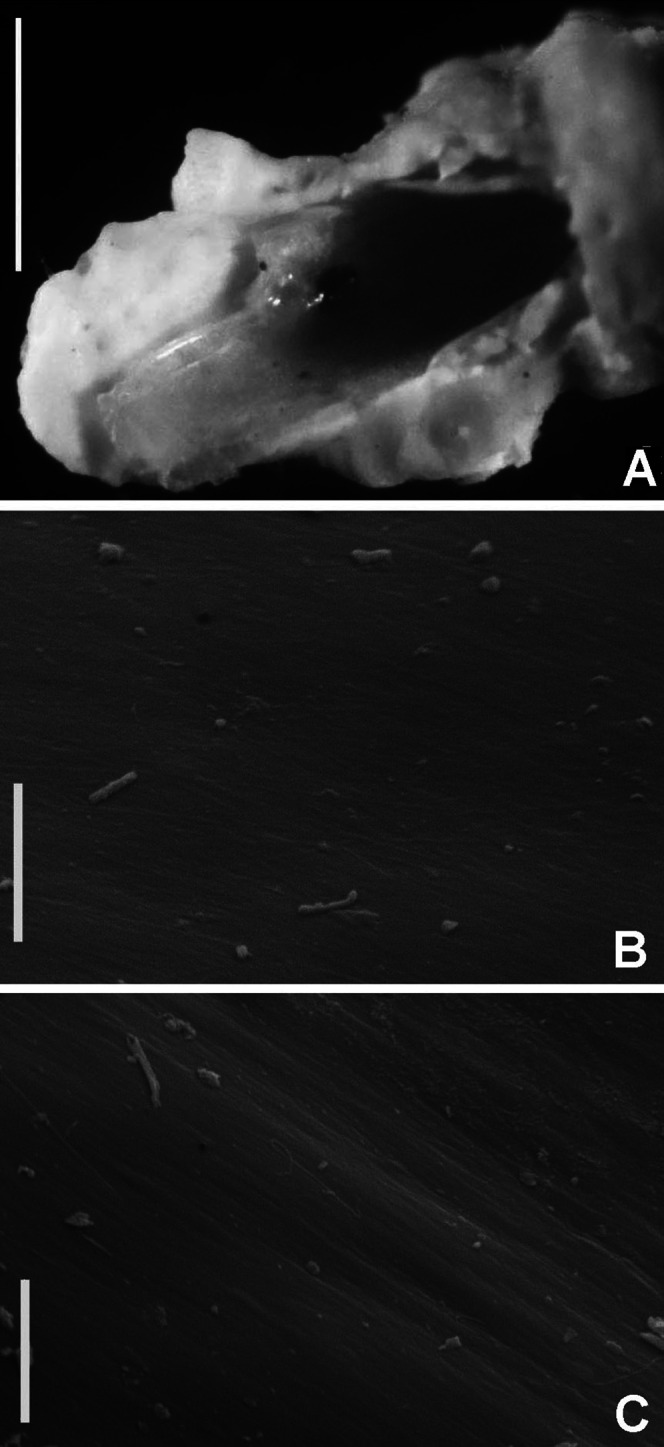
Tubes and microstructure of *Pseudopotamilla oligophthalmos*. (A) Hyaline tube covered by calcareous matter, (B) surface of lumen showing fibers of various orientations, (C) lumen with longitudinal microrelief. Scale bars: (A) 1 mm, (B and C) 5 μm. Specimens: RMNH.VER. 19948.

*Sabella* (*Potamilla*) *oligophthalmos* Grube, 1878: 248−249; Wiktor, 1980: 280, two syntypes Museum of Natural History, Wrocław University, MPW 368.

*Potamilla laciniosa* Ehlers, 1904 *fide* Augener, 1914: 253; Augener, 1926: 109; Johansson, 1927: 151, but see remarks.

*Potamilla oligophthalmos*.— Augener, 1914: 109–113.

*Potamilla polyophthalmos* Grube, 1878 *fide* Augener, 1914: 255.

*Potamilla* (*Pseudopotamilla*) *oligophthalmos*.— Augener, 1926: 253–255.

*Potamilla oligophthalma*.— Glasby, Lee & Hsueh, 2016: 213.

**Material examined.** Indonesian-Dutch Snellius II Expedition, Sta. 4.051, NE coast of Sumba, E of Melolo, 9°53.5′S, 120°42.7′E, calcareous stones, rich epifauna dominated by soft corals, in coral rocks, rectangular dredge, 75–90 m depth, September 13, 1984, 4 specimens [RMNH.VER. 19948]. Sta.4.062, NE coast of Sumba, E of Melolo, 09°53.5′S, 120°44.5′E, rectangular dredge, some calcareous stones, sponges dominant, 125 m depth, September 15, 1984, 3 specimens sharing rocks with *Notaulax* sp. [RMNH.VER. 19949]. Sta. 4.067, Indonesia, NE coast of Sumba, 09°57′S 120°49′E, rectangular dredge, 45 m depth, sandy bottom virtually without stones, fauna dominated by sponges, September 16, 1984, 1 specimen [RMNH.VER. 19950].

**Description.** *Colour, body shape, and size*. Body pale. Second and third thoracic segments with dorsal whitish glandular areas; anterior one is better defined than posterior one (Figs. 18C, 18F, and 18G). Body depressed. Trunk 4–25 mm long, 0.4–1.2 mm wide.

*Radiolar crown*. Length 2.2–6.5 mm. Six to 10 pairs of radioles. Radiolar lobes short, with dorsal and ventral flanges: dorsal pair triangular, ventral pair rounded. Radiolar flanges and palmate membrane absent. Pinnules long, arranged in two alternating rows, progressively longer (Fig. 19A). Radiolar tips short, as long as space of 2–3 pinnules (Fig. 19A). Compound eyes proximal in dorsal radioles (basal half of crown), absent in dorsal-most pair; circular or oval (Figs. 18A and 19B), unequal in size and number: R=x 3 3 2 2 1 1 1 1 x (specimen 1); R=x 2 2 1 2 1 1 x x (specimen 2); R=x 2 2 2 2 2 1 1 x x (specimen 3).

*Peristomium*. Anterior peristomial ring partially exposed dorsally. Posterior peristomial ring collar with dorsal margins fused to faecal groove; mid-dorsal margins long, triangular (Figs. 18B and 18C), extending to base of radioles, covering 3/4 of branchial lobe dorsal flanges; lateral margins U-shaped (Fig. 18A). Ventral lappets triangular (Fig. 18E), divided mid-ventrally by a deep incision (1/3 collar length). Dorsal lips triangular, erect, with long radiolar appendages; ventral lips short, rounded. Ventral sacs present.

*Thorax*. Chaetiger 1: two rows of broadly-hooded notochaetae (Fig. 18C), ventral shield entire, rectangular (Figs. 18D and 18E). Chaetigers 2–10: superior notochaetae elongate narrowly hooded (Fig. 18D); inferior ones paleate, arranged in two rows, with pointed mucro (Fig. 19E). Neuropodial avicular uncini with several rows of small, similar sized teeth above main fang; breast well developed, handles long, three times longer than main fang (Fig. 19F). Companion chaetae with asymmetrical membranes and long handles, slightly longer than handles of uncini (Fig. 19F). Ventral shields rectangular, divided transversely (Fig. 18D). Tori not contacting ventral shields (Fig. 18B).

*Abdomen*. Segments: 78. Neurochaetae elongate broadly-hooded. Notopodial uncini with several rows of teeth above main fang; the latter extending along 2/3 of its length, breast well developed, handles short. Pygidium unknown.

*Tubes*: The tube is embedded in calcareous material secreted by the coral (Fig. 18H), its organic wall is translucent (Fig. 20A).

*Sex and gametes*. Unknown.

**Tube microstructure.** Tube lumen relatively smooth with some faint longitudinal relief at some places. Fibers of at least four different orientations visible on the lumen surface; however, fibers are poorly developed and difficult to see. Interspaces of fibers much wider than fiber diameter. Fibers straight, relatively long (>5 μm), thin (0.10–0.15 μm wide). Interspaces of adjacent fibers filled with homogeneous organics with smooth surface. Tube wall dense, non-porous. There is not much similarity in tube microstructure with the coral symbiont *Perkinsiana anodina*, nevertheless both species show multiple orientations of fibers (Figs. 20B and 20C).

**Remarks.** The original description for *Sabella* (*Potamilla*) *oligophthalmos* Grube, 1878, stated the presence of a few eyes along the basal half of dorsal radioles, except on the dorsal most one (a diagnostic feature for *Pseudopotamilla*), and the presence of long and narrow dorsal lappets. In his description, Grube (1878) also provided a comparison of his new species with *Pseudopotamilla reniformis* (as *Potamilla*). Augener (1914) reported *Potamilla oligophthalmos* from Western Australia and later, as *Potamilla* (*Pseudopotamilla*) *oligophthalmos* from New Zealand (Augener, 1926). In our study, *Pseudopotamilla oligophthalmos* is redescribed using specimens from the North East coast of Sumba, that were found from 45 to 125 m depth, in dead coral blocks which agrees with the original description.

*Pseudopotamilla oligophthalmos* differs from *P. monoculata* in features as number and distribution of radiolar eyes and collar morphology (Table 7). The presence of glandular areas like those visible in *P. oligophthalmos* have only been reported for *Pseudopotamilla platensis* (Hartman, 1953), a species from Argentina, reported up to a depth of 200 m (Tovar-Hernández, de León-González & Bybee, 2017).

**Table 7:** Comparison of *Pseudopotamilla monoculata* and *P. oligophthalma*.

Feature	*Pseudopotamilla monoculata* Capa, 2007	*Pseudopotamilla oligophthalmos* (Grube, 1878), new combination
Eyes	One big, elongated compound eye proximally on each radiole, except for dorsalmost pair and some ventral radioles; eyes diminishing progressively in size dorsally to ventrally	1–2 eyes in most radioles, except dorsalmost (but can be up to 3), circular to oval-shaped, variable in size
Dorsal margin of collar	*V*-shaped	*U*-shaped
Dorsal lappets	Present, low, rounded	Present, high, triangular
Ventral lappets	Divided by a short incision	Divided by a long incision
Dorsal glandular shields	Not reported	Present on 2nd and 3rd thoracic chaetigers
Type locality	Tasmania, Tasman Sea, E of Maria Island	Singapore

Augener (1914) proposed two changes in the status of other taxa: he referred *Potamilla laciniosa* Ehlers, 1904 to *Potamilla oligophthalmos* (Grube, 1878); and synonymized *Sabella* (*Potamilla*) *polyophthalmos* Grube, 1878 (from the Philippines) with *Sabella* (*Potamilla*) *oligophthalmos* Grube, 1878 (from Singapore), giving the latter priority. It is remarkable that Capa (2007: 559) mentioned some papers to have considered *P. laciniosa* as valid species, but she did not provide sources for this opinion, these were probably Hartmann-Schröder (1989) and Glasby et al. (2009), both listed in WoRMS (Read & Fauchald, 2020c). Although types of *P. polyophthalmos* and *P. oligophthalmos* were not examined by Augener (1914) to support this synonymy, Augener (1926, p. 111) explained that apart from differences in the number of radiolar eyes, *Potamilla polyophthalmos* and *P. oligophthalmos* belong to a single species. He did not explain why *oligophthalmos* should have priority over *polyophthalmos*, perhaps it was merely based on alphabetic order: in the original descriptions *P. polyophthalmos* was described and figured first (Grube, 1878: 247−248, pl. 15, fig. 2), *P. oligophthalmos* came later in the text and was not figured (Grube, 1878: 248−249), one might expect Augener’s priority proposal to have been the other way around. *Pseudopotamilla polyophthalmos* was described with 5−6 eyes per radiole, with a maximum of 10 (many = *poly-*), whereas *P*. *oligophthalmos* only with 1−2 eyes on most radioles (exceptionally up to 3; few = *oligo-*), except for dorsalmost pair. In our study, we prefer to keep both species separate, until a detailed revision of the supposed synonymy based on the examination of Grube’s type material and comparison with Augener’s specimens will prove otherwise.

The prominent eyespots in (some) species of the genus *Pseudopotamilla* resulted in three homonyms for two specific epithets: “*oligophthalmos*” and “*polyophthalmos*”. *Pseudopotamilla oligophthalmos* originally described as *Sabella* (*Potamilla*) *oligophthalmos* was referred to *Pseudopotamilla* by Hartman (1959: 557), making *Pseudopotamilla oligophthalma* (Iroso, 1921) homonymous. The latter was described from Naples as *Potamilla oligophthalma* and considered to be a synonym of *Pseudopotamilla reniformis* by Fauvel (1927) and Hartman (1959). Recently, Knight-Jones et al. (2017) attributed *P. oligophthalma* (Iroso) to *Pseudotamilla saxicava* (de Quatrefages, 1866), making action on the homonymy of *P. oligophthalma* (Iroso) not necessary.

*Pseudopotamilla polyophthalmos* is dealt with below.

***Pseudopotamilla*** ***polyophthalmos* (Grube, 1878), new combination**

*Sabella* (*Potamilla*) *polyophthalmos* Grube, 1878: 247−248, pl. 15, fig. 2.

**Remarks.** Grube (1878) described *Sabella* (*Potamilla*) *polyophthalmos* from the Philippines. His description, emphasizing and illustrating the presence of compound eyes in most radioles, except the dorsalmost, matches *Pseudopotamilla*. Its species have 5−6 eyes per radiole, with a maximum of 10. In our opinion it is a valid species. As indicated with the diagnosis of the genus *Pseudopotamilla*, above, a worldwide revision is desirable, based on examination of types and topotypical material.

By transferring *Sabella* (*Potamilla*) *polyophthalmos* Grube to the genus *Pseudopotamilla* as we propose, the new combination has two secondary homonyms: *P. polyophthalmos* (Langerhans, 1884) (from Madeira) and *P. polyophthalma* Hartmann-Schröder, 1965 (from Punta Lavapié, central Chile). Examination of types is certainly needed, but the Madeiran species seems to be a junior synonym of *P. saxicava* (see Knight-Jones et al., 2017), in which case no further action is needed for that species-group name.

The recombined species group name *Pseudopotamilla polyophthalmos* (Grube) and *P. polyophthalma* Hartmann-Schröder belong to two different genera making action on the homonym of *P. polyophthalma* Hartmann-Schröder not necessary (see below).

***Pseudopotamilla*** ***polyophthalma*Hartmann-Schröder, 1965, homonym, probably *Potaspina*Hartman, 1969**

*Pseudopotamilla polyophthalma* Hartmann-Schröder, 1965: 271–273, figs 273–275.

**Remarks.** Hartmann-Schröder (1965) described *Pseudopotamilla polyophthalma* from Punta Lavapié, central Chile, based on the presence of compound eyes in dorsal and lateral radioles (except in dorsalmost and 3 ventralmost pairs): L=x 1 2 3 2 1 x x x, R=x 1 2 2 1 1 x x x. Eyes are largest in the third and fourth radioles, and smallest in radioles second and sixth (Hartmann-Schröder, 1965). However, she also described and illustrated the presence of modified “hooks” in thoracic chaetigers 7 and 8, replacing uncini; this feature is not present in *Pseudopotamilla* species (Knight-Jones et al., 2017). Acicular spines or “hooks” replacing uncini in last thoracic segments is an autapomorphy for *Potaspina* Hartman, 1969 (Fitzhugh, 1989; Capa, 2007), and consequently *Pseudopotamilla polyophtalma* might need to be moved to that genus.

However, *Potaspina* includes two species: *P. pacifica* Hartman, 1969 (with acicular spines in thoracic chaetigers 5–7), and *P. australiensis* Capa, 2007 (with acicular spines in thoracic chaetigers 7–9); each species was described on a single specimen, and eyes were not reported for any of them. In addition, Capa’s specimen has some radioles with tips missing and under histolysis. Thus, the Hartmann-Schröder’ species has an uncertain taxonomic position. It cannot be a member of *Pseudopotamilla* but its placement in *Potaspina* is not fully supported due the presence of compound eyes on its radioles. Revision of type material of *P. polyophthalma*, deposited in the Invertebrates II Zoological Collection of the University of Hamburg, as well as additional material from California and Australia, in order to corroborate the presence of radiolar eyes in *Potaspina pacifica* and *P. australiensis*, and to assign *P. polyophthalma* to *Potaspina*, or to propose a new genus for it.

**Genus *Sabellastarte*Krøyer, 1856** (p. 13)

*Sabellastarte*.— Fitzhugh, 1989: 72–73; Knight-Jones & Mackie, 2003: 2272; Capa et al., 2019: 201–202.

**Type species**: *Sabella indica* Savigny, 1822, designated by Bush (1905).

**Number of species**: 8, after Knight-Jones & Mackie (2003).

**Remarks.** Diagnoses to genus level have been provided by Fitzhugh (1989), Tovar-Hernández & Salazar Vallejo (2006), Knight-Jones & Mackie (2003) and Capa et al. (2019). Capa, Bybee & Bybee (2010), combining morphological and molecular data of species of *Sabellastarte*, revealed that at least six lineages are present within the genus and two were potentially new cryptic species.

***Sabellastarte spectabilis* (Grube, 1878)**

(Fig. 21)

**Figure 21:**
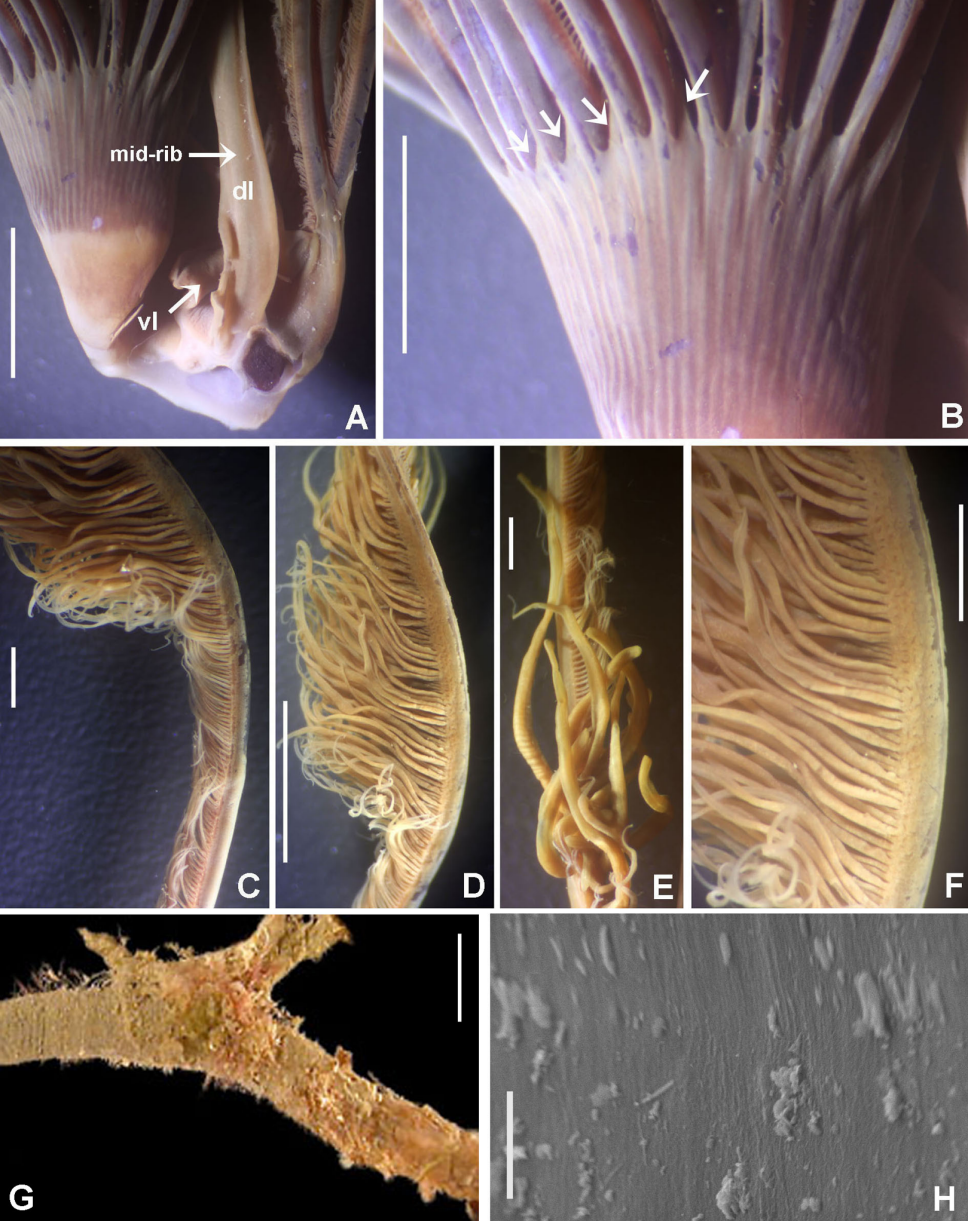
*Sabellastarte spectabilis* and tube microstructure. (A) Right branchial lobe, radioles almost in complete circle, (B) arrows indicating interdigitating radioles, (C–F) enlarged, wide and flattened pinnules, (G) tube, (H) surface of lumen showing fibers of single orientation. Scale bars: (A and B) 0.5 mm, (C) 1 mm, (D) 3.5 mm, (E and F) 1 mm, G (8 mm), (H) 5 μm. Abbreviations: dl, dorsal lip; vl, ventral lip. Specimen, RMNH.VER. 19951.

*Sabella indica* Savigny, 1822: 77.

*Sabella spectabilis* Grube, 1878: 253, pl. 14, fig. 4; Augener, 1914: 115; Treadwell, 1920: 600–601; Ehlers, 1920: 69; Wiktor, 1980: 281; 8 syntypes in Museum of Natural History, Wrocław University, MPW 374; Hartwich, 1993: 138; Grube’s material in Berlin lost.

*Sabella notata* Grube, 1878: 256; Wiktor, 1980: 280, holotype Museum of Natural History, Wrocław University, MPW 367; Hartwich, 1993: 121, holotype Zoologisches Museum, Berlin, ZMB Ver. 1554 (the existence of two holotypes, one in Wrocław, one in Berlin, of course is impossible. Probably the marking as holotype in Berlin was executed by a conservator or visiting polychaetologist long before Hartwich compiled his catalogue, and the latter overlooked the record in Wiktor).

*Sabellastarte indica*.— Augener, 1933: 199; Augener, 1934: 111 (not *S. magnifica* Shaw, 1800); Fauvel, 1939: 360.

*Sabellastarte spectabilis*.— Knight-Jones & Mackie, 2003: 2274–2278, fig. 2 (indication of lectotype; synonymy of *Sabella notata*; however, their record of ‘*Sabella indica* Abildgaard, 1789*’* is mistaken, see discussion on nomenclature, below). Glasby, Lee & Hsueh, 2016: 214.

**Material examined.** Indonesian-Dutch Snellius II Expedition, Sta. 4079A, E of Komodo, Selat Linta, 08°35′S, 119°34.2′E, edge of coastal reef flat, 3–6 m depth, September 18, 1984, 1 specimen [RMNH.VER. 19951]. Sta. 4.069B, E of Komodo, Teluk Slawi, northern cape of entrance, 08°36′S, 119°31.2′E, coral heads and isolated corals on sandy bottom, 9–16 m depth, September 17, 1984, 5 specimens [RMNH.VER. 19952]. Sta. 4151. NE of Takabonerate (Tiger Island), middle of reef flat, Taka Garlarang atoll, 06°27′S, 121°14′E, September 27, 1984, 1 specimen [RMNH.VER. 19953].

**Description.** Radioles inrolled ventrally, almost forming a complete circle on each side (Fig. 21A). Dorsal basal flanges D-shaped. Palmate membrane short, as long as width of radiolar rachis. Interdigitated radioles alternatingly: directed more or less outwards, respectively inwards (arrows in Fig. 21B). Six radioles (lateral, dorsal and ventral radioles) with peculiar pinnules located at mid-radiolar length: pinnules wide (2–3 times wider than proximal pinnules, flat and long) (Figs. 21C–21F). Radiolar tips long, free for the width of 6–7 pinnules. Dorsal lips long with mid-rib or radiolar appendage (Fig. 21A). Posterior peristomial ring collar with dorsal margins fused to faecal groove, with deep notches above dorsal pockets; lateral margins entire, transverse to body axis. Ventral lappets sub-triangular, not overlapping. Ventral shield of collar longer than those of thorax, rectangular, with anterior margin M-shaped. Ventral shields of thorax progressively smaller. Neuropodial tori contacting ventral shields. Abdomen with 163 segments. Pygidial eyespots absent. Pygidium rim-shaped.

*Tubes*: The tube is rigid, composed of fine sand (Fig. 21G).

**Tube microstructure.** Tube lumen relatively smooth; poorly developed fibers with the same general orientation. Fibers short (usually shorter than 5 μm); single fibers 0.10 to 0.20 μm wide. Fibers can be slightly curved. Tube wall dense, non-porous (Fig. 21H).

**Remarks.** Our specimens match the description by Knight-Jones & Mackie (2003) for *S. spectabilis* from Bohol, Philippines. However, the presence of some radioles with hypertrophied pinnules (wide, flat and long at medium length of radioles) has not been recorded in any species of *Sabellastarte*. It is remarkable that these unusual pinnules were only seen in the largest specimen. It is not clear if this modification is functional, or due to disease. Spermatids, coelomocytes or full developed gametes were not found in our specimens [RMNH.VER. 19951]. However, *S. spectabilis* from Hawaii is a protandric hermaphrodite (Bybee, Bailey-Brock & Tamaru, 2006), while the same species is reported to be gonochoric in Micronesia (Rouse & Fitzhugh, 1994). This indicates that more than one species may be included under the same name.

On the other hand, Augener (1933) reported *Sabellastarte magnifica* Shaw, 1800 from Biliton (Belitung) Island, Indonesia. He emphasized that *S. indica* Savigny, 1822 and the species *S. magnifica* from the West Indies are synonyms. This synonymy was not followed by Knight-Jones & Mackie (2003), they regarded Indo-West Pacific and Caribbean taxa as separate species. In addition, Capa, Bybee & Bybee (2010) found different lineages between specimens attributed to *Sabellastarte* from the Caribbean and the Indopacific (Malaysia and Saipan), and concluded they are separate species.

**Nomenclatural discussion on the use of *Sabellastarte spectabilis* (Grube, 1878) over *S. indica* (Savigny, 1822) and their authorship**

Knight-Jones & Mackie (2003: 2269) stated that *Sabella indica* Savigny, 1822 (subsequently designated as type-species of *Sabellastarte* by Bush (1905)) is preoccupied by the pectinariid *Sabella indica* Abildgaard, 1789, making it necessary to find a different type species for the genus *Sabellastarte*, for which they selected *Sabella spectabilis* Grube, 1878. This is incorrect because the type species “remains unchanged even when it is a junior synonym or homonym, or a suppressed name” (ICZN, 1999, Art. 67.1.2). However, while reviewing original data sources in order to track this history, we found some discrepancies and inconsistencies necessitating a separate contribution, which will not complete without examination of the syntypes of *Sabella indica* deposited at the Museum National d’Histoire Naturelle, Paris (MNHN POLY TYPE 608, 609).

Firstly, in Abildgaard (1789) nowhere the Latin name “*indica*” can be found. Abildgaard only compares a large pectinariid from the East Indies with *Amphitrite auricoma* Müller, 1776 and *Sabella granulata* Linnaeus, 1767, both taxa belonging to the family Pectinariidae according to WoRMS (Read & Fauchald, 2020d). Under this scenario, there is no homonymy for *Sabella indica* Savigny, 1822, and the new proposal of *Sabellastarte spectabilis* (Grube) as type would be incorrect.

Second, there is confusion about the authorship of *Sabella indica*. It can be found in the literature and WoRMS as: *Sabella indica* Gmelin in Linnaeus, 1788; *Sabella indica* Savigny in Lamarck, 1818 and *Sabella indica* Savigny, 1822 (Hartman, 1959; Knight-Jones & Mackie, 2003; Read & Fauchald, 2020e). In this contribution we follow Knight-Jones & Mackie (2003) but is it clear that *Sabellastarte indica* and *S. spectabilis* require a thorough revision.

**On the microstructure of organic tube wall in sabellids**

All studied sabellids have a tube wall with a purely organic composition. This organic tube wall has a lamellar microstructure; lamellae are composed of fine, long fibers. The fibers in the single lamellae of some species seem to have a similar general orientation (i.e., *Acromegalomma acrophthalmos*, *Sabellastarte spectabilis*), but in other species fiber orientation varies. Usually lamellae of the tube wall are composed of fibers with alternate orientations. There can be up to four different main orientations of fibers in a single lamella. In case of two alternate orientations, fibers are usually located at angles of 35–40° to each other (i.e., *Bispira porifera*). In case of three different orientations, two of these are at an angle of 45°, respectively 90° as compared with the first (i.e., *Perkinsiana anodina*). In *Pseudopotamilla oligophthalma* the orientation of fibers do not fall into well-defined categories. The diameters of fibers seem to be similar in most species studied, being between 0.10 and 0.20 μm, nevertheless, in *B. porifera* fibers are slightly thicker.

## Conclusions

The Indonesian archipelago, the South China and the Philippine Seas are an important marine biodiversity hotspot with especially rich marine life. Until now, 23 genera and 78 species of Sabellidae were reported from the whole area (Treadwell, 1920; Augener, 1933; Mesnil & Fauvel, 1939; Pillai, 1965; Gallardo, 1968; Nishi, 1998; Fitzhugh, 2002; Al-Hakim & Glasby, 2004; Capa, 2007, Capa, 2008; Capa & Murray, 2009; Salazar-Vallejo et al., 2014; Glasby, Lee & Hsueh, 2016; Nishi et al., 2017; Hadiyanto, 2018; Nishi, Tanaka & Tovar-Hernández, 2019; Pamungkas & Glasby, 2019, and the present study). This represents 15.82% of the 493 species, and 57.5% of the 40 genera of sabellids currently known in the entire world (Pamungkas et al., 2019). Surely these numbers will increase, at least 11 taxa mentioned as “spec.” are now already waiting for formal description (Fitzhugh, 2002; Al-Hakim & Glasby, 2004; this study). Certainly, through exploration of other habitats and depths with the new sampling techniques, and with modern methods for identifying and analyzing biodiversity, we expect a significant raise in numbers. Special attention should be given to molecular analyses that allow for distinction between cryptic “look-alike” taxa, as well as invasive species.

## Additional Information and Declarations

### Data Availability

The following information was supplied regarding data availability: Body measurements are located in each species description. All specimens examined were deposited at Naturalis Biodiversity Center (Leiden, the Netherlands) under the following catalogue numbers: *Acromegalomma acrophthalmos* (Grube, 1878) [RMNH.VER. 19926]. *Acromegalomma interruptum* (Capa & Murray, 2009) [RMNH.VER. 19927]. *Acromegalomma* sp. 1 [RMNH.VER. 19928]. *Acromegalomma sumbense* sp. nov. Tovar-Hernández, ten Hove & de León-González [RMNH.VER. 19929], [RMNH.VER. 19930]. *Bispira manicata* (Grube, 1878) [RMNH.VER. 19931]. *Bispira secusoluta* (Hoagland, 1920) [RMNH.VER. 19933]. *Bispira porifera* (Grube, 1878) [RMNH.VER. 19932]. *Branchiomma boholense* (Grube, 1878) [RMNH.VER. 19934], [RMNH.VER. 19935], [RMNH.VER. 19936], [RMNH.VER. 19937], [RMNH.VER. 19938]. *Claviramus olivager* sp. nov. Tovar-Hernández, ten Hove & García-Garza [RMNH.VER.19939]. *Notaulax pyrrhogaster* (Grube, 1878) [RMNH.VER. 19940]. *Notaulax tenuitorques* (Grube, 1878) [RMNH.VER. 19941]. *Notaulax* sp. 3 [RMNH.VER. 19942]. *Notaulax montiporicola* sp. nov. Tovar-Hernández & ten Hove [RMNH.VER. 19943], [RMNH.VER. 19944]. *Parasabella crassichaetae* Capa & Murray, 2015b [RMNH.VER. 19945]. *Perkinsiana anodina* Capa, 2007 [RMNH.VER. 19946], [RMNH.VER. 19947]. *Pseudopotamilla oligophthalmos* (Grube, 1878) [RMNH.VER. 19948], [RMNH.VER. 19949], [RMNH.VER. 19950]. *Sabellastarte spectabilis* (Grube, 1878) [RMNH.VER. 19951], [RMNH.VER. 19952], [RMNH.VER. 19953].

### New Species Registration

The following information was supplied regarding the registration of a newly described species: Publication LSID: urn:lsid:zoobank.org:pub:382D313F-0138-4194-B3B2-5BA84817374A. *Acromegalomma sumbense* sp. nov. Tovar-Hernández, ten Hove & de León-González, LSID: urn:lsid:zoobank.org:act:A5F4957B-3DC0-49CA-B779-B10DCC80869B. *Claviramus olivager* sp. nov. Tovar-Hernández, ten Hove & García-Garza, LSID: urn:lsid:zoobank.org:act:F8BA6972-4240-4042-A7DF-FC089AC7A2D2. *Notaulax montiporicola* sp. nov. Tovar-Hernández & ten Hove, LSID: urn:lsid:zoobank.org:act:E2790CCF-B27C-44BF-BB96-A0F4D378317B.
